# Chirogenesis and Pfeiffer Effect in Optically Inactive Eu^III^ and Tb^III^ Tris(β-diketonate) Upon Intermolecular Chirality Transfer From Poly- and Monosaccharide Alkyl Esters and α-Pinene: Emerging Circularly Polarized Luminescence (CPL) and Circular Dichroism (CD)

**DOI:** 10.3389/fchem.2020.00685

**Published:** 2020-08-11

**Authors:** Michiya Fujiki, Laibing Wang, Nanami Ogata, Fumio Asanoma, Asuka Okubo, Shun Okazaki, Hiroki Kamite, Abd Jalil Jalilah

**Affiliations:** ^1^Division of Materials Science, Graduate School of Science and Technology, Nara Institute of Science and Technology, Ikoma, Japan; ^2^School of Materials Engineering, Universiti Malaysia Perlis, Jejawi, Malaysia; ^3^Centre of Excellence Frontier Materials Research, Universiti Malaysia Perlis, Kangar, Malaysia

**Keywords:** non-covalent interaction, circularly polarized luminescence, circular dichroism, europium, terbium, cellulose, saccharide, terpene

## Abstract

We report emerging circularly polarized luminescence (CPL) at 4*f-*4*f* transitions when lanthanide (Eu^III^ and Tb^III^) tris(β-diketonate) embedded to cellulose triacetate (**CTA**), cellulose acetate butyrate (**CABu**), *D*-/*L*-glucose pentamethyl esters (***D*-**/***L*-Glu**), and *D*-/*L*-arabinose tetramethyl esters (***D*-**/***L*-Ara**) are in film states. Herein, 6,6,7,7,8,8,8-heptafluoro-2,2-dimethyl-3,5-octanedionate (fod) and 2,2,6,6-tetramethyl-3,5-heptanedione (dpm) were chosen as the β-diketonates. The *g*_lum_ value of Eu(fod)_3_ in **CABu** are +0.0671 at 593 nm (^5^${\text{D}}_{0}\to^{7}$F_1_) and −0.0059 at 613 nm (^5^${\text{D}}_{0}\to^{7}$F_2_), respectively, while those in **CTA** are +0.0463 and −0.0040 at these transitions, respectively. The *g*_lum_ value of Tb(fod)_3_ in **CABu** are −0.0029 at 490 nm (^5^${\text{D}}_{4}\to^{7}$F_6_), +0.0078 at 540 nm (^5^${\text{D}}_{4}\to^{7}$F_5_), and −0.0018 at 552 nm (^5^${\text{D}}_{4}\to^{7}$F_5_), respectively, while those in **CTA** are −0.0053, +0.0037, and −0.0059 at these transitions, respectively. ***D*-**/***L*-Glu** and ***D*-**/***L*-Ara** induced weaker *g*_lum_ values at 4*f-*4*f* transitions of Eu(fod)_3_, Tb(fod)_3_, and Tb(dpm)_3_. For comparison, Tb(dpm)_3_ in α-pinene showed clear CPL characteristics, though Eu(dpm)_3_ did not. A surplus charge neutralization hypothesis was applied to the origin of attractive intermolecular interactions between the ligands and saccharides. This idea was supported from the concomitant opposite tendency in upfield ^19^F-NMR and downfield ^1^H-NMR chemical shifts of Eu(fod)_3_ and the opposite Mulliken charges between *F*-C bonds (fod) and *H*-C bonds (**CTA** and ***D*-**/***L*-Glu**). An analysis of CPL excitation (CPLE) and CPL spectra suggests that (+)- and (–)-sign CPL signals of Eu^III^ and Tb^III^ at different 4*f-*4*f* transitions in the visible region are the same with the (+)-and (–)-sign exhibited by CPLE bands at high energy levels of Eu^III^ and Tb^III^ in the near-UV region.

## Introduction

In recent years, controlled chirogenesis led by several scenarios of intermolecular chirality transfer endowed with natural and human-made resources has become the most popular phenomenon allowing for an efficient generation of the desired optically active substances in the realms of organic chemistry (Bosnich, 1967; Hayward and Totty, 1969; Noack, 1969; Soai et al., 2019), supramolecular chemistry (Kobayashi et al., 1993; Huang et al., 1998; Prince et al., 2000; Borovkov et al., 2004; Hembury et al., 2008; Aida et al., 2012; Borovkov, 2014; Liu et al., 2015; Goto et al., 2017), polymer chemistry (Green et al., 1993; Yashima et al., 1995; Nakashima et al., 2001; Kawagoe et al., 2010; Numata and Shinkai, 2011; Lee et al., 2012; Duan et al., 2014; Fujiki, 2014; Wang et al., 2014; Akagi, 2019), and molecular aggregation/colloidal/gel chemistry (Palmans and Meijer, 2007; Isare et al., 2010; George et al., 2011; Mei et al., 2015; Roose et al., 2016; Sang et al., 2019). Particularly, chirogenesis in metal coordination chemistry by the chirality transfer has long been one of the central subjects in inorganic chemistry (Mason and Norman, 1965; Kirschner and Ahmad, 1968; Kirschner and Bakkar, 1982; Mason, 1982; Brittain, 1983, 1989; Riehl and Richardson, 1986; Tsukube and Shinoda, 2002; Di Bari and Salvadori, 2005; Muller, 2009, 2014; Bünzli, 2010; Carr et al., 2012; Tanner, 2013; Miyake, 2014; Kumar et al., 2015; Zinna and Di Bari, 2015; Kono et al., 2016; Longhi et al., 2016; Lunkley et al., 2018; Wong et al., 2019).

Historically, in 1898, Kipping and Pope investigated the first chirogenesis of *L*-NaClO_3_ crystals (*P*2_1_3 space group) in an enantiomer excess (*ee*) of several % that were preferentially grown in a water solution of *D*-glucose and *D*-mannitol (Kipping and Pope, 1898). In 1919, Perucca found the first chirogenesis of triarylmethane textile dye by observing an anomalous optical rotational dispersion (ORD) in visible region when he dispersed an ORD-silent triarylmethane textile dye in polycrystalline *L*-NaClO_3_ (Perucca, 1919; Kahr and Gurney, 2001; Jacoby, 2008; Bing et al., 2010). In the early 1930s, Pfeiffer and Quehl were aware that optical rotation of amino acids increased and/or decreased in the presence of racemic labile metal (Zn^2+^, Cd^2+^, and Ni^2+^) complexes (Pfeiffer and Quehl, 1931, 1932). This anomaly is called as the *Pfeiffer effect* (Mayer and Brasted, 1973; Mason, 1982; Brittain, 1983, 1989; Lunkley et al., 2018).

In 1965, Mason and Norman reported the first circular dichroism (CD) signals at 3*d-*3*d* transitions of Co^III^(NH_3_)_6_(ClO${}_{4}^{-}$)_3_ in the presence of (+)-diethyl tartrate (Mason and Norman, 1965). In 1977, the first circularly polarized luminescence (CPL) spectra from optically inactive Eu^III^(fod)_3_, Eu^III^(dpm)_3_, Tb^III^(fod)_3_, and other two Eu^II^ complexes dissolved in (*R*)-/(*S*)-α-phenylethylamine are reported by hypothesizing the Pfeiffer effect (fod = 6,6,7,7,8,8,8-heptafluoro-2,2-dimethyl-3,5-octanedionate, dpm: dipivaloylmethane or 2,2,6,6-tetramethyl-3,5-heptanedione) (Brittain and Richardson, 1977a; Brittain, 1980). These pioneering CPL studies further stimulated many researchers to investigate CPL and CD spectroscopic characteristics of optically inactive lanthanide complexes in the presence of amino acids, monosaccharides, malic acid ascorbic acid, cyclic glycols, DNA, and other chiral chemical influences (Luk and Richardson, 1974; Brittain and Richardson, 1977b; Madaras and Brittain, 1980; Brittain, 1981, 1984; Richardson, 1982; Yan et al., 1982; Huskowska and Riehl, 1995; Muller and Riehl, 2005; Muller, 2009; Iwamura et al., 2012, 2016; Miyake et al., 2014; Wu et al., 2016, 2019; Jalilah et al., 2018; Lunkley et al., 2018; Wu and Bouř, 2018; Taniguchi et al., 2019). An outer-sphere intermolecular interaction between the Δ-/Λ-mixture lanthanide^III^ (Ln^III^) complex and the chiral additives is responsible for the equilibrium shift, as detectable by emerging CPL spectra. Richardson et al. evaluated that the barrier heights in Δ*-*Λ stereomutation between charged M^+^[Eu(dpa)_3_]^−^ (M^+^; counter cationic species and dpa; dipicolinate) at the photoexcited state (ES) in achiral solvents are in the range of 12 and 17 kcal mol^−1^; higher viscosity cosolvents of water-ethylene glycol corresponded to higher barrier heights (Glover-Fischer et al., 1998).

**Figure 1:**
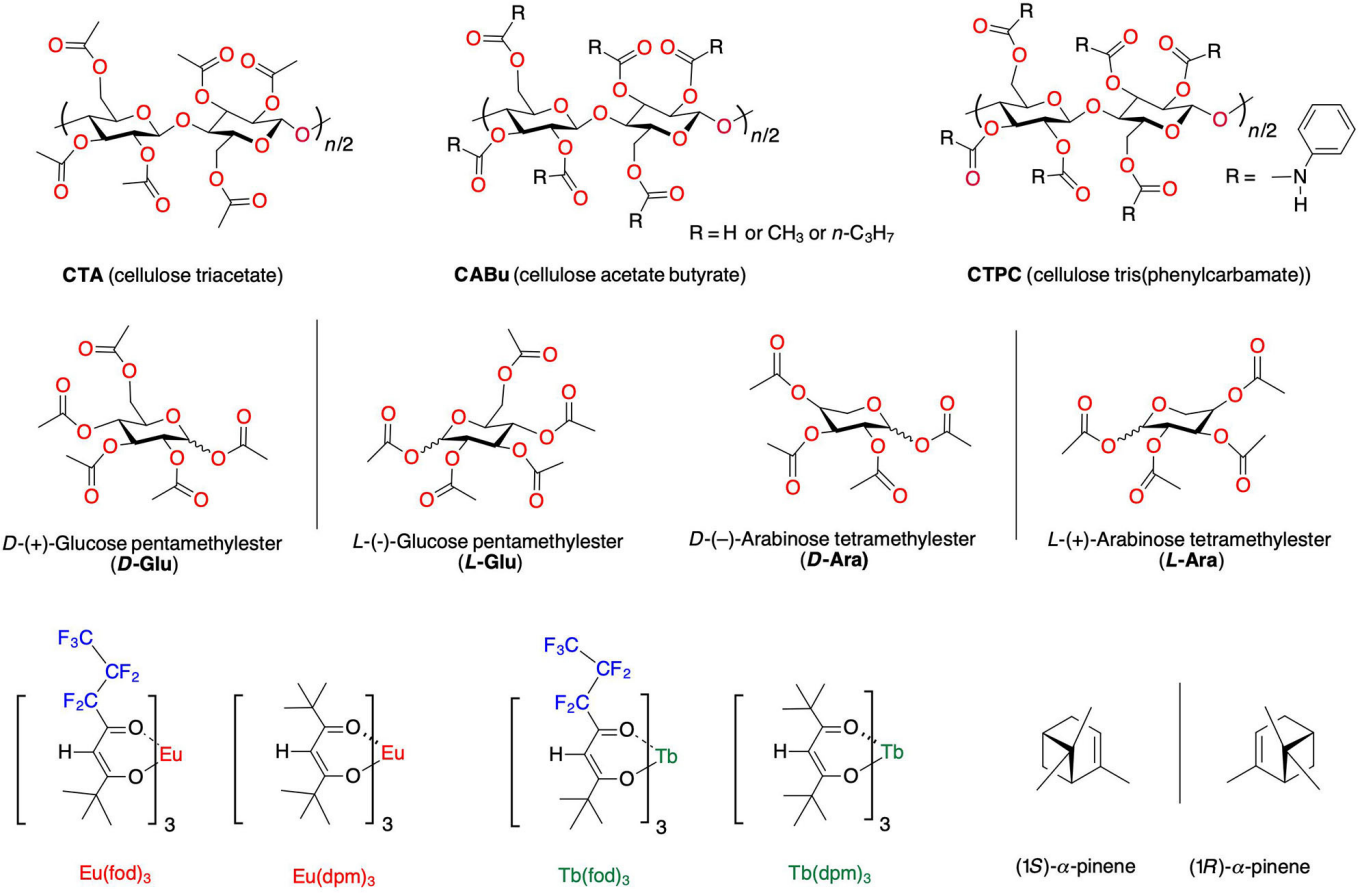
The chemical structures of cellulose alkyl esters (**CABu** and **CTA**), cellulose tris(phenylcarbamate) (**CTPC**), *D*-(+)- and *L*-(–)-glucose pentamethylester (***D*-**/***L*-Glu**), *D*-(–)- and *L-(*+*)-*arabinose tetramethylester (***D*-**/***L*-Ara**), (1*S*)-/(1*R*)-α-pinene, and four optically inactive Eu^III^ and Tb^III^ complexes coordinated with achiral tris(β-diketonate) [Eu(fod)_3_, Tb(fod)_3_, Eu(dpm)_3_, and Tb(dpm)_3_].

New knowledge and understanding of several weak inter- and intramolecular interactions known as C-*H/*π, C-*H*/*O*-C, C-*H*/*F*-C, C-*F*/*H*-O, C-F/Si, and π-π (Nishio et al., 1998; Desiraju and Steiner, 1999; Matsuura et al., 2003; Mele et al., 2003; Tsuzuki et al., 2003; Kim et al., 2004; Mahadevi and Sastry, 2016; Pitts et al., 2017) lead us to hypothesize that multiple point chirality and/or main chain helicity of *non-charged* bioresources impose the capability of non-symmetrical force transfer to *non-charged* achiral or optically inactive chromophores and luminophores. *Non-charged* terpene hydrocarbons, e.g., (*S*)-/(*R*)-limonene, (1*S*)-/(1*R*)-α-pinene, and (1*S*)-/(1*R*)-β-pinene, can efficiently work as chiral liquified scaffolds in the ground state (GS) and the ES of several colloidal π-/σ-conjugated polymers (Kawagoe et al., 2010; Nakano et al., 2010, 2012; Lee et al., 2012; Fujiki, 2014; Wang et al., 2014), colloidal vinyl polymers bearing azobenzene (Jiang et al., 2015), and *non-charged* Eu(fod)_3_ (Figure 1; Jalilah et al., 2018). However, to realize practical photonic applications, solution-processable and/or thermoplastic solid materials are inevitably needed because terpenes are volatile, flammable liquids.

Among solidified chiral bioresources, poly- and monosaccharides are incredible biomaterials that are abundant on earth and have a wide variety of functions (Klemm et al., 2005), e.g., allowing growth and maintenance of homochiral life resulting chiral foods (Coultate, 2016), recognition and sorting of enantiomers (Ikai and Okamoto, 2009), service as a building block of optically active supramolecular complexes (Numata and Shinkai, 2011), and fabrication of liquid crystals, solid films, fibers, sheets, and nano-composites for industrial purposes (Dubois et al., 1998; Henriksson et al., 2007; Wang et al., 2007; Iwatake et al., 2008). More recently, poly- and monosaccharides are excellent chiroptical platforms to generate and boost CD/CPL/circularly polarized reflection bands because of their helicoidal and cholesteric higher-order structures (Wilts et al., 2017; De La Cruz et al., 2018; Yu et al., 2018, 2019; Zheng et al., 2018). We have proved that three cellulose derivatives (cellulose triacetate (**CTA**), cellulose acetate butyrate (**CABu**), and cellulose tris(phenyl carbamate) (**CTPC**) in Figure 1 possess an efficient ambidextrous scaffolding capability toward CD-inactive oligo- and polyfluorenes, leading to the corresponding (+)- or (–)-sign CPL-active/CD-active species as the solid film states (Guo et al., 2017, 2018).

These results encourage us to propose that **CTA**, **CABu**, *D*-/*L*-glucose permethyl esters (***D*-Glu**/***L*-Glu**), and *D*-/*L*-arabinose permethyl esters (***D*-Ara**/***L*-Ara**) should work as chirality transferring solid platforms, enabling several optically inactive and/or racemic Eu^III^ and Tb^III^ complexes to the corresponding CPL-active/CD-active, non-racemic species (Figure 1). For comparison, we tested the chirality transfer capability of (1*S*)-/(1*R*)-α-pinene as chiral liquid molecules to Eu(dpm)_3_ and Tb(dpm)_3_.

Herein, we showcase that Eu(fod)_3_, Tb(fod)_3_, and Tb(dpm)_3_, except Eu(dpm)_3_ commonly exhibited CPL signals at 4*f-*4*f* transitions and CD bands due to *n-*π^*^/π*-*π^*^ transitions of the ligands when **CABu**, **CTA**, ***D*-Glu**/***L*-Glu**, and ***D*-Ara**/***L*-Ara** were employed as embedding films. In α-pinene, although Tb(dpm)_3_ showed clear CPL characteristics, Eu(dpm)_3_ did not. Unresolved inherent nature between Eu^III^ and Tb^III^ causes considerable differences of CPL characteristics as well as emission wavelengths at 4*f-*4*f* transitions. Although the current *g*_lum_ values of our CPL-active lanthanide complex in the films are not very outstanding, the hypothesis of these non-covalent weak chiral intermolecular interactions in the ES and GS led us to freely design solution processible CPL- and/or CD-functioned composite films made of several optically inactive lanthanide complexes coordinated with achiral organic ligands upon chirality/helicity transfer of inexpensive soluble polysaccharide, oligosaccharide, and monosaccharide derivatives as the solidified chiral bioresources, in addition to a conventional multi-step synthesis of optically active lanthanide complexes coordinated with chiral ligands designed strategically.

## Experimental

### Materials

#### CTA and CABu

**CTA** (Wako Pure Chemicals, Osaka, Japan) and **CABu** (Sigma-Aldrich Japan, Tokyo, Japan) were used without further purification (Figures S1, S2, SM). Chloroform, tetrahydrofuran (THF), methanol (MeOH), and other solvents (Dojindo, Kumamoto, Japan) were used as received. CDCl_3_ and hexafluorobenzene (C_6_F_6_) were purchased from Stable Isotope Lab (SIL) (Japanese vendor, Wako Pure Chemicals) and Wako Pure Chemicals, respectively. (1*S*)- and (1*R*)-α-Pinene (Tokyo Chemical Industry (TCI), Tokyo, Japan) were purified by distillation in a reduced pressure.

#### Monosaccharide Permethylesters

***L*-(-)-Glu**. To a mixture of *L*-(-)-glucose (0.5 g) and stoichiometric acetic anhydride (4 mL), freshly dried Cu(OTf)_2_ (0.03 mol % of *L*-(-)-glucose) at 0°C was added under nitrogen [Scheme S1 in Supplementary Materials (SM)]. The mixture was stirred for 1 h in an ice bath and then stirred at room temperature for 12 h. Methanol (5 mL) was slowly added to quench the acylation reaction, and the mixture was stirred for another 0.5 h, followed by evaporation under reduced pressure. Chloroform (25 mL) was added to dissolve the residue, and the mixture was consecutively washed twice with saturated NaHCO_3_ brine and water, respectively. The organic layer was dried over anhydrous Na_2_SO_4_, and the mixture was filtered. The filtrate was concentrated in a vacuum and purified by silica gel column chromatography, and eluted with petroleum ether/ethyl acetate (v/v, 50/1) to yield a white powder (yield: 0.42 g, 70%; Analysis: calculated (%) for C_16_H_22_O_11_: C, 49.23; H, 5.68; found (%): C, 49.53; H, 5.43. ^1^H-NMR (in CDCl_3_), FT-IR (onto CaF_2_) and ESI (positive mode)-MS spectra of ***L*-Glu** and ***D*-Glu** are shown in Figure S3, SM and Figure S4, SM, respectively. Elemental analysis of ***L*-Glu** and ***D*-Glu** are given in Figure S5, SM.

***D*-(+)-Glu**. Donated from Prof. Wei Zhang (Soochow University, China) prepared by a similar method. ^1^H-NMR, FT-IR, and ESI (positive mode) MS spectra are displayed in Figure S4, SM. Solid-state ^13^C-NMR and ^1^H-NMR in CDCl_3_ solution are shown in **Figures 5**, **8A**, respectively. Analysis: Calcd (%) for C_16_H_22_O_11_: C, 49.23; H, 5.68; Found (%): C, 49.51; H, 5.40.

***D*-Ara and** ***L*-Ara**. *D*-(-)-Arabinose tetramethyl ester and *L*-(+)-arabinose tetramethyl ester were synthesized utilizing the same procedure as described for the synthesis of ***L*-Glu**. A viscous liquid was obtained as ***D*-Ara**. Yield: 2.4 g, 32%. Analysis for ***D*-Ara**: Calcd (%) for C_13_H_18_O_9_: C, 49.06; H, 5.70, Found (%): C, 48.97; H, 5.56. ***L*-Ara**. Yield (2.4 g, 32%). Analysis: Calcd (%) for C_13_H_18_O_9_: C, 49.06; H, 5.70, Found (%): C, 49.01; H, 5.60. ^1^H-NMR, FT-IR, and ESI-MS (positive mode) spectra of ***D*-Ara** and ***L*-Ara** are shown in Figures S6, S7, SM, respectively. The results of the elemental analysis of ***L*-Ara** and ***D*-Ara** are shown in Figure S8, SM.

#### Tb(fod)_3_

The starting material, 0.19 g (0.74 mmol) of TbCl_3_ (99% purity, Sigma-Aldrich, now Merck) was dissolved in the minimum amount of methanol (3.0 mL), and 1,1,1,2,2,3,3-heptafluoro-7,7-dimethyl-4,6-octanedione (Hfod, 0.636 g (2.15 mmol, 0.5 mL), TCI) was adjusted to pH 5–6 by adding the required amount of aqueous NaOH solution. The above two solutions were mixed by vigorous stirring with a magnetic stir bar for 10 min, followed by addition of 200 mL distilled water dropwise into the solution. A pale-yellow Tb(fod)_3_ was precipitated under vigorous stirring with a magnetic bar for 12 h. The crude product adhered to the bottom of the reaction vessel was purified by a short-column silica gel chromatography (Wakogel C-200, Chart S1 in SI) as shown below, with chloroform as an eluent to yield a pale yellow oil, followed by drying in a vacuum oven 90°C to obtain a white solid. Yield, 200 mg (40%). The purification of Tb(fod)_3_ using a 5 mL pipette tip made of polypropylene, as a short-column apparatus, is illustrated below. Analysis: Calcd (%) for C_30_H_30_F_21_O_6_Tb: C, 34.50; H, 2.90. Found (%): C, 34.58; H, 2.80. The elemental analysis suggested that Tb(fod)_3_ has no water adducts.

Before we utilized the purification method (see Chart S1, SM) for Tb(fod)_3_, we were aware of some impurity peaks approximately 450 nm in the PL spectrum of Tb(fod)_3_. After the purification by silica gel column chromatography and elution with chloroform, we obtained a pure Tb(fod)_3_ (based on the expected fluorescent emission spectrum). ^1^H-NMR (CDCl_3_), ^19^F-NMR (CDCl_3_), FT-IR (CaF_2_), and ESI-MS (positive mode) spectra and elemental analysis of Tb(fod)_3_ are shown in Figure S9, SM.

#### Other Lanthanide Complexes

Eu(fod)_3_, Eu(dpm)_3_, and Tb(dpm)_3_ were purchased from Sigma-Aldrich-Merck, TCI, and Sigma-Aldrich-Merck, respectively, and were used as received. ^1^H-NMR (CDCl_3_) and FT-IR spectra of Eu(fod)_3_ are shown in Figure S10, SM. ^1^H-NMR and FT-IR spectra of Eu(dpm)_3_ are shown in Figure S11, SM. ^1^H- and ^19^F-NMR (CDCl_3_) and FT-IR (CaF_2_) spectra of Tb(dpm)_3_ are shown in Figure S12, SM.

### Instrumentation

The UV-vis and CD spectra of the solutions were measured with a JASCO J-820 spectropolarimeter (Hachioji-Tokyo, Japan) equipped with Peltier-controlled housing units. Synthetic quartz (SQ) cuvette with a 10-mm path length (scanning rate: 100 nm min^−1^; bandwidth: 1.0 nm; response time: 1.0 s; 0.5-nm interval sampling; single accumulation) at 25°C were used. To avoid second- and third-order stray light due to diffraction grating, CPL and PL spectra were recorded on a JASCO CPL-200, that was designed as a prism-based spectrofluoropolarimeter with a forward scattering of 0° angle equipped with focusing and collecting lenses, and a manually movable film holder onto an optical rail enables to adjust the best focal point to maximize CPL/PL signal amplitudes. Measurement conditions were bandwidths of 10 nm for excitation and emission, a scanning rate of 100 nm min^−1^, and a data sampling of 0.5 nm interval. IR spectra were measured on CaF_2_ plate using a Horiba FT-730 Fourier-transform (FT) infrared (IR) spectrometer (Horiba, Kyoto, Japan) over a wavenumber range between 800 and 4,000 cm^−1^ with a resolution of 2 cm^−1^ and a scanning speed of 5 mm s^−1^ for 128 scans and a Perkin-Elmer Spectrum One/100 FT-IR spectrometer (Winter Street Waltham, MA 02451, USA) over a wavenumber range between 900 and 4,000 cm^−1^ with a resolution of 4 cm^−1^ for 64 scans. Electrospray ionization mass spectrometry (ESI-MS) was conducted with a JEOL (Akishima, Tokyo, Japan) AccuTOF JMS-T100 LC mass spectrometer (accelerating voltage, 10 kV). Electron ionization mass spectrometry with high-resolution (HR-EI-MS) mode was recorded with a JEOL JMS-700 double-focusing mass spectrometer (accelerating voltage, 10 kV). The ionic species were often attached with Na^+^ ion. The hybridized polymers were characterized by a JEOL JNM-ECX400 cross-polarization (CP) magic-angle-spinning (MAS) solid-state (ss)-^13^C{H}-FT-NMR spectrometer (resonance frequency 100.5 MHz, contact time 2.0 ms, 550 scans, relaxation delay 5.0 s, spinning 8.0 kHz, repetition time 5.05 s). Elemental analysis was performed on a Perkin-Elmer 2400II CHNS/O. The solution ^1^H- and ^19^F-FT-NMR spectra were recorded on the JEOL ECP-400 spectrometer. The resonance frequencies of ^19^F- and ^1^H-NMR are 376 MHz and 400 MHz, respectively. Representative measurement conditions for ^19^F-NMR spectra had an acquisition time of 0.432 sec, 64 acquisitions, a relaxation delay of 4.0 sec, at a temperature of ~20°C, a pulse angle of 45° and a pulse width of 7.0 sec were used. Raw NMR data were processed and analyzed by JEOL Delta (Ver. 5) software. Hexafluorobenzene (HFB, −163.0 ppm) and tetramethylsilane (Me_4_Si, 0.0 ppm) were used as internal standards for the ^19^F- and ^1^H-NMR measurements, respectively. Photodynamic decay of six solid films (Eu(fod)_3_ with **CTA** and **CABu** (detected at 610–620 nm), Eu(dpm)_3_ with **CTA** and **CABu** (detected at 610–620 nm), and Tb(dpm)_3_ with **CTA** and **CABu** (detected at 542–551 nm) excited by an N_2_ laser (Usho KEC-160; wavelength 337.1 nm; pulse width 600 ps; 10 Hz) were measured with the help of streak camera (Hamamatsu, picosecond fluorescence measurement system C4780 with Grating 150 lines per mm and slit width 100 μm). The 337.1 nm of N_2_ laser source was used to excite shoulder UV/CD signals of the lanthanide complexes. Photodynamic measurements of other Eu^III^ and Tb^III^ complexes in the ***D*-/*L*-Glu** and ***D*-/*L*-Ara** films excited at 337.1 nm were not successful. For simplicity, the emission lifetime was evaluated by single exponential decay analysis. Quantum yields of the Eu^III^ and Tb^III^ complexes in the solid films were not obtained due to lack of an integrating sphere. The all processed data saved as raw text data were re-organized by KaleidaGraph ver. 4.53 (Synergy software, Reading, PA 19606, USA).

### Preparation of the Hybridized Films

In fabricating the hybridized film, 10 mg of lanthanide complexes and 20 mg of saccharide derivatives (**Glu** and **Ara**) or cellulose derivatives (**CABu** and **CTA**) were completely dissolved in 1.0 mL of the desired solvent (chloroform or tetrahydrofuran (THF)) at ambient temperature. The hybridized film was deposited onto a polished circular quartz plate or borosilicate glass (Tempax Float®, Schott AG. Germany) (25 mm in diameter and 1 mm in thickness) by spin coating using a spin coater (MIKASA, MS-B100, Tokyo, Japan), then, 800 μL of the solution was placed onto the center of the plate and spun at 1,500 rpm for 60 s. The films on the glass were attached on both sides (front and back surfaces) to ensure an *optically symmetrical geometry* with air-sample-(quartz or borosilicate substrate)-sample-air contact by spin coating chloroform or THF solutions that consist of saccharides (chiral host) and lanthanide complex (achiral guest) (Guo et al., 2017, 2018; Yamada et al., 2018). Although the film thicknesses of both sides were not determined, we assumed to be on the order of several μm for each. The hybridized double-side coating films were scattering-free and transparent by the naked eye. To measure CPL/PL/CPLE/PLE/CD/UV-visible spectra, the optical density of the double-side coating specimen was controlled to 0.3–1.0 in the range of 280 and 330 nm. CD, CPL, and CPLE spectra of the hybridized films were measured at ambient temperature (24–26°C). The double-sided coating in the symmetrical optical geometry avoids chiroptical inversion artifacts that could be originated from linear dichroism induced by mechanical stress on anisotropic specimens due to spin coating. Based on our experience, single-side coating in the dissymmetrical optical geometry can often cause artifact inversion in signs of CPL and CD signals. In the case of single-side coating, the probability of the chiroptical sign inversion was approximately 2–3 out of 10, while double-side coating prevented the artifact origin sign inversion.

## Results and Discussion

The chirogenesis characteristics of oligo-/polyfluorenes originate from rotatable C–C bonds between fluorene rings and from C–O/C–C bonds of the cellulose derivatives (Figure 1) (Guo et al., 2017, 2018; Yamada et al., 2018) in the GS and ES because rotational barrier heights of the single bonds are as small as 1.5–2.5 kcal mol^−1^. Eu^III^ and Tb^III^ complexes with three fod ligands should coexist as racemic mixtures of *D*-/*L*-species of *C*_3_-symmetrical facial (*fac*)- and *C*_1_-meridional (*mer*) motifs (Brittain and Richardson, 1977a; Jalilah et al., 2018), while even Eu^III^ and Tb^III^ with three dpm should coexist as a racemic mixture of *D*-/*L*-species of *D*_3_-geometry. Although barrier heights of *D*-*L* stereomutation and/or *fac*-*mer* isomerisms are considerably high on the order of 10–20 kcal mol^−1^ (Glover-Fischer et al., 1998; Carr et al., 2012; Miyake, 2014), multiple intermolecular C-*H*/*O*-C, C-*H*/π, and C-*H*/*F*-C interactions (Murray-Rust et al., 1983; Nishio et al., 1998; Desiraju and Steiner, 1999; Tsuzuki et al., 2003; Yuasa et al., 2011; Koiso et al., 2017; Jalilah et al., 2018) should overcome the barriers when solidified matrices are employed. Note that solidified matrices are regarded as solid-like solvents with a very high viscosity. In this work, we applied a double-side, spin-coating technique (Guo et al., 2017, 2018) to fabricate CPL-/CD-functioned films deposited onto fused quartz and/or borosilicate glass to obtain artifact-free CPL/photoluminescence (PL), CPLE/PL excitation (PLE), and CD/UV-visible spectra. CPL and CPL spectral characterizations of Eu^III^ and Tb^III^ at 4*f-*4*f* transitions were assigned based on the literature (Fulgêncio et al., 2012; Tanner, 2013; Binnemans, 2015; de Queiroz et al., 2015; Xue et al., 2015; Yang et al., 2017). Dimensionless Kuhn's anisotropic ratios in the ES and GS, being popularly known as *g*_lum_ and *g*_abs_, were manually evaluated at a specific extreme wavelength (λ_ext_) of the corresponding CPL and CD spectral profiles in line with the literature (Eliel and Wilen, 1994). All CPL characteristics (*g*_lum_ value at λ_ext_) of Eu^III^ and Tb^III^ complexes are summarized in Table 1.

**Table 1:** CPL characteristics (dissymmetry ratio, *g*_lum_ in 10^−2^ at specific wavelength) of Eu^III^ and Tb^III^ coordinated with three β-diketonates as achiral ligands embedded in two polysaccharide alkyl esters (**CABu** and **CTA**), *D*-/*L*-glucose pentamethyl esters (***D*-**/***L****-***Glu**), and *D*-/*L*-Arabinose tetramethyl esters (***D*-/*L-*Ara**).

**Ln^III^ tris(β- diketonates)**	**CABu *g*_lum_/10^−2^ (nm)**	**CTA *g*_lum_/10^−2^ (nm)**	**Glu *g*_lum_/10^−2^ (nm)**		**Ara *g*_lum_/10^−2^ (nm)**		***α-*pinene *g*_lum_/10^−2^ (nm)**	
			***D*-**	***L*-**	***D*-**	***L*-**	**(1*R*)**	**(1*S*)**
Eu(fod)_3_	+6.71 (593)^a^ −0.59 (613)^b^	+4.63 (593)^a^ −0.40 (613)^b^	+1.05 (594)^a^ −0.19 (612)^b^	−0.81 (596)^a^ +0.08 (613)^b^	+0.19 (593)^a^ −0.02 (607)^b^	−0.30 (591)^a^ +0.06 (611)^b^	−0.49 (593)^f^ +0.05 (613)^f^	+0.41 (593)^f^ −0.04 (613)^f^
Eu(dpm)_3_	n.d. ^g^	n.d. ^g^	n.d. ^g^	n.d. ^g^	n.d. ^g^	n.d. ^g^	n.d. ^g^	n.d. ^g^
Tb(fod)_3_	−0.29 (490)^c^ +0.78 (540)^d^ −0.18 (552)^e^	−0.10 (490)^c^ +0.35 (542)^d^ −0.07 (553)^e^	n.d. ^g^	n.d. ^g^	n.d. ^g^	n.d. ^g^	n.d. ^g^	n.d. ^g^
Tb(dpm)_3_	−0.53 (491)^c^ +0.37 (537)^d^ −0.59 (547)^e^	−0.44 (489)^c^ – +0.80 (547)^e^	n.d. ^g^	n.d. ^g^	n.d. ^g^	n.d. ^g^	n.d^g^ (~490) +0.44^d^ (537) −0.13^e^ (547)	n.d^g^ (~490) −0.49^d^ (537) +0.34^e^ (548)

*All numerical values in bracket mean wavelength extremum for CPL signals*.

a *Eu^III^^5^${\text{D}}_{0}\to^{7}$F_1_ (593 nm)*,

b *Eu^III5^${\text{D}}_{0}\to^{7}$F_2_ (613 nm)*,

c *Tb^III^^5^${\text{D}}_{4}\to^{7}$F_6_ (490 nm)*,

d *Tb^III5^${\text{D}}_{4}\to^{7}$F_5_ (I) (540 nm)*,

e *Tb^III5^${\text{D}}_{4}\to^{7}$F_5_ (II) (552 nm)*,

f *Data were taken from literature (Jalilah et al., 2018)*.

g *Not characterized or no data*.

### Chirality Transfer Capability From Cellulose Alkyl Esters to Eu(fod)_3_

**Figure 2:**
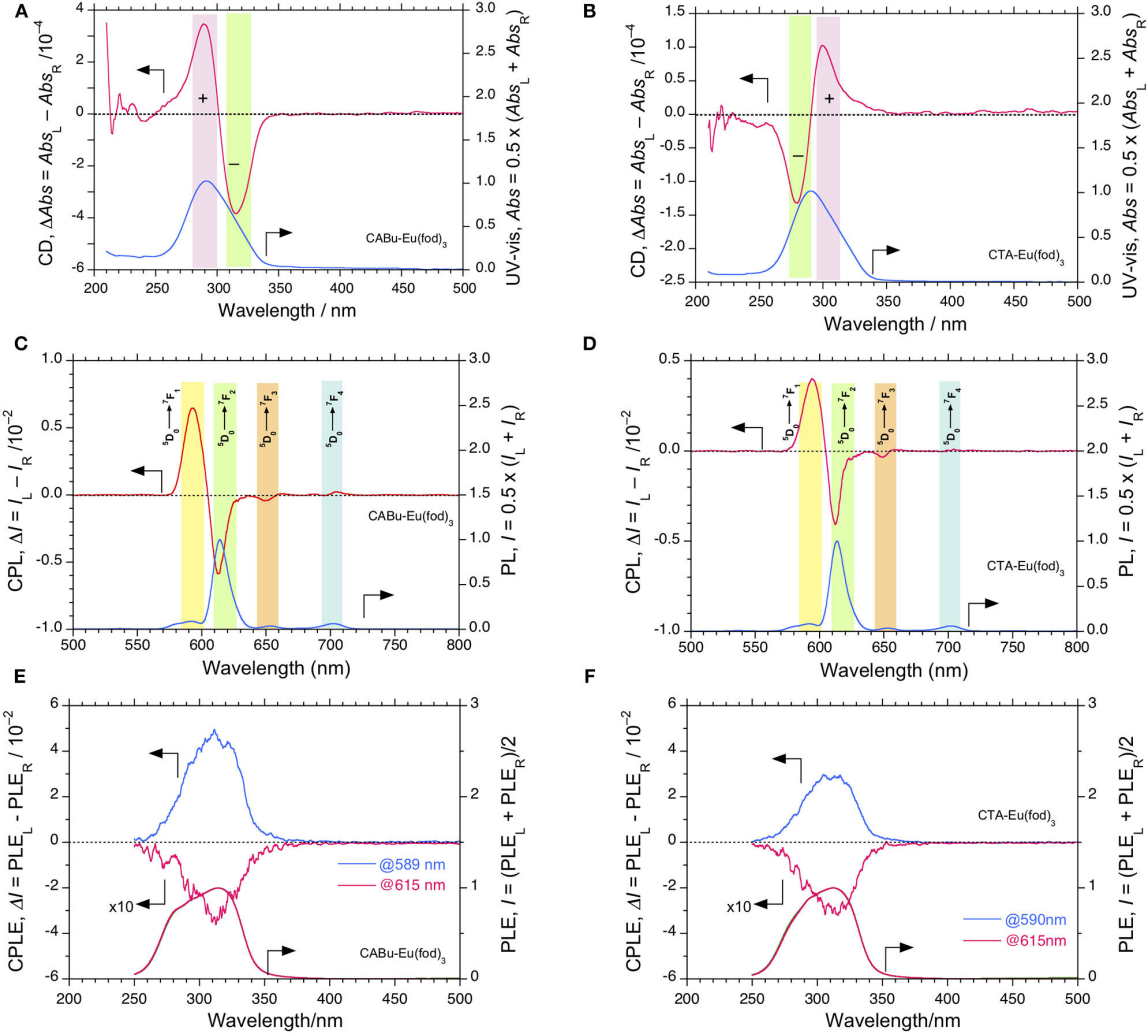
CD and UV-visible spectra of Eu(fod)_3_ in **(A) CABu** film and **(B)** in **CTA** film. CPL and PL spectra (excited at 315 nm) of Eu(fod)_3_ in **(C) CABu** film and **(D)** in **CTA** film (excited at 315 nm). **(E)** CPLE and PLE spectra monitored at 589 and 615 nm of Eu(fod)_3_ in **CABu** film. **(F)** CPLE and PLE spectra of Eu(fod)_3_ monitored at 590 and 615 nm in **CTA** film. The cellulose and Eu(fod)_3_ were hybridized at a weight ratio of 20 mg/10 mg in chloroform solution.

The normalized CD and UV-visible spectra of Eu(fod)_3_ in **CABu** and **CTA** films are shown in Figures 2A,B, respectively. For comparison, the original raw CD and UV-visible spectra of the Eu(fod)_3_-hybridized films were given in Figure S13A, SM. Bisignate profile at Cotton CD bands at 290 and 310 nm between **CABu** and **CTA** films are obviously opposite. These Cotton CD bands at 290 and 310 nm, however, do not originate from **CABu** and **CTA**. Broad monosignate CD bands due to *n*-π^*^ transition from alkyl esters of **CABu** and **CTA** thin films appeared at ~215 nm with (+)-sign and ~205 nm with (–)-sign, respectively (Guo et al., 2018). These (+)-and (–)-sign CD bands at 205 and 215 nm in the solid film reflect from left-handed helicity of **CABu** and right-handed helicity of **CTA** in solutions, respectively (Dubois et al., 1998; Onofrei et al., 2015), though **CABu** and **CTA** are β-(1 → 4) linked polymers made of *D*-glucose framework as a common repeating unit.

Although the alkyl ester itself does not have a stereogenic center, the ester can adopt particular chiral conformational geometry by the direct connection of the *D*-glucose ring. Two lone pairs at ethereal “–*O*–” and two C–H groups at “–C*H*_2_-” are no longer to be equal because of C–*O*–C single bonds in R–C(=O)–*O*–CH_2_- side group act as pseudochiral stereogenic bonds, similar to gauche *n*-butane. The unequal lone pairs at ethereal oxygen and unequal CH_2_ groups may be responsible for the induction of chiral intermolecular C–*O*/*H*–C and C–*H*/*F*–C interactions between the alkyl ester moieties and lanthanide ligands. However, any CD/CPL signals of Eu(fod)_3_ in the presence of **CABu** and **CTA** in dilute chloroform solutions (~10^−3^ M) were not able to detect because the postulated chiral intermolecular C–O/H–C and C–H/F–C interactions are inherently weak in the fluidic solution. The postulated chiral alkyl esters of **CABu** and **CTA** can thus act efficiently and differently in the solidified films only as external chirality inducible scaffoldings toward optically inactive Eu(fod)_3_ and several lanthanide complexes, as discussed in later sections.

The *g*_abs_ values at λ_ext_ of Eu(fod)_3_ at 290 and 316 nm in **CABu** film are +3.5 × 10^−4^ at 280 nm and −3.5 × 10^−4^ at 310 nm, respectively, while those in **CTA** film are −1.6 × 10^−4^ at 290 nm and +1.1 × 10^−4^ at 300 nm, respectively. Note that the λ_max_ values at non-polarized UV-visible spectra of Eu(fod)_3_ in **CABu** and **CTA** films are commonly ~291 nm. Although these CD bands at ~290 nm and ~310 nm are ascribed to *n*-π^*^/^1^π-^3^π^*^ bands of the three fod ligands, their signs appear to be determined solely by preferential helix sense and/or local chirality of multiple alkyl esters of **CABu** and **CTA**.

Contrarily, the normalized bisignate-like CPL spectral profiles between Eu(fod)_3_ in **CABu** and **CTA** films are apparently the same, as shown in Figures 2C,D, respectively. Obviously, bisignate CPL band profiles at ~593 nm and ~613 nm in **CABu** and **CTA** are definitively similar. The *g*_lum_ values of Eu(fod)_3_ in **CABu** film are +6.71 × 10^−2^ at ^5^${\text{D}}_{0}\to^{7}$F_1_ transition (593 nm) and −0.59 × 10^−2^ at ^5^${\text{D}}_{0}\to^{7}$F_2_ transition (613 nm), respectively, while those in **CTA** film are weaker with +4.63 × 10^−2^ at ^5^${\text{D}}_{0}\to^{7}$F_1_ transition (593 nm) and −0.40 × 10^−2^ at ^5^${\text{D}}_{0}\to^{7}$F_2_ transition (613 nm), respectively (Table 1).

It is interesting to note that the absolute magnitude |*g*_lum_| values at ^5^${\text{D}}_{0}\to^{7}$F_1_ band of Eu(fod)_3_, 6.7 × 10^−2^ in **CABu** and 4.6 × 10^−2^ in **CTA** are considerably boosted by 13–16 times and 9–11 times, respectively, relative to the |*g*_lum_| values of Eu(fod)_3_ in neat (*R*)- and (*S*)-α-pinene (Jalilah et al., 2018). Also, those |*g*_lum_| values are enhanced by 2.5–2.7 times and 1.7–1.9 times compared to those of Eu(fod)_3_ in neat (*R*)- and (*S*)-α-phenylethylamine (Jalilah et al., 2018). Although the |*g*_lum_| values of Eu(fod)_3_ in **CABu** and **CTA** are not outstanding compared to those several Eu^III^ complexes coordinated with well-designed chiral ligands reported recently (Petoud et al., 2007; Lunkley et al., 2008; Leonzio et al., 2017; Yeung et al., 2017; Zhou et al., 2019), the **CABu** and **CTA** have a tremendous benefit as chiral solidified platforms to efficiently induce and magnify |*g*_lum_| values to optically inactive Eu(fod)_3_ as a potent CPL emitter regardless of achiral ligands.

To confirm the apparent inconsistency between the retention in CPL bands at 4*f-*4*f* transitions in **CABu** and **CTA** films and between the inversion in CD bands at *n*-π/^1^π-^3^π^*^ transitions in **CABu** and **CTA** films, we applied CPLE and PLE spectroscopy (Duong and Fujiki, 2017) by monitoring at ^5^${\text{D}}_{0}\to^{7}$F_1_ and ^5^${\text{D}}_{0}\to^{7}$F_2_ transitions in **CABu** and **CTA** films, as shown in Figures 2E,F, respectively. Disregard of **CABu** and **CTA**, it is obvious that the CPLE band at 310 nm is commonly (+)-sign monitored at ^5^${\text{D}}_{0}\to^{7}$F_1_ transition and that the CPLE band at 310 nm is commonly (–)-sign monitored at ^5^${\text{D}}_{0}\to^{7}$F_2_ transition. The magnitudes of the CPLE bands in **CABu** film are +4.86 × 10^−2^ monitored at 589 nm and −0.30 × 10^−2^ monitored at 615 nm, respectively. Similarly, the magnitude of the CPLE bands in **CTA** film somewhat weaken, and +2.96 × 10^−2^ monitored at 590 nm and −0.33 x 10^−2^ monitored at 615 nm, respectively.

The origin of the inconsistency between the sign at the first Cotton CD band (310 nm) and the opposite CPLE sign at this wavelength that depends on the wavelengths monitored at ^5^${\text{D}}_{0}\to^{7}$F_1_ and ^5^${\text{D}}_{0}\to^{7}$F_2_ transitions is an unresolved question and obscure. However, the *n*-π/^1^π-^3^π^*^ bands at ~310 nm with the opposite sign originates from the three fod ligands, is obviously degenerative, and is responsible for LMCT (from the ligands to high energy levels of Eu^III^, for example,^5^D_2_ state), leading to ^5^${\text{D}}_{0}\to^{7}$F_1_ and ^5^${\text{D}}_{0}\to^{7}$F_2_ transitions with the opposite CPL sign. The broader ~310 nm transition is likely to be a convolution of two nearly degenerate transitions with an opposite chirality; one (+)-sign band at ~310 nm is responsible for ^5^${\text{D}}_{0}\to^{7}$F_1_ and another (–)-sign band at ~310 nm for ^5^${\text{D}}_{0}\to^{7}$F_2_ bands. The ^5^D_2_ state of Eu^III^ is close to the lowest photoexcited T_1_ states of the ligands. When one excite simultaneously at couplet-like ^1^π-^3^π^*^ transitions (~310 nm) of CD-active Eu(fod)_3_ using monochromated non-polarized light, the photoexcited Eu(fod)_3_ decays into the ^7^F_1_ and ^7^F_2_ states with two different pathways because ^5^${\text{D}}_{0}\to^{7}$F_1_ and ^5^${\text{D}}_{0}\to^{7}$F_2_ states are magnetic dipole (MD) allowed transition with an electric dipole (ED) forbidden transition and MD forbidden transition with a forced induced ED transition, so-called, hypersensitive transition, respectively (Tanner, 2013; Binnemans, 2015).

### Chirality Transfer Capability From Cellulose Alkyl Esters to Tb(fod)_3_

**Figure 3:**
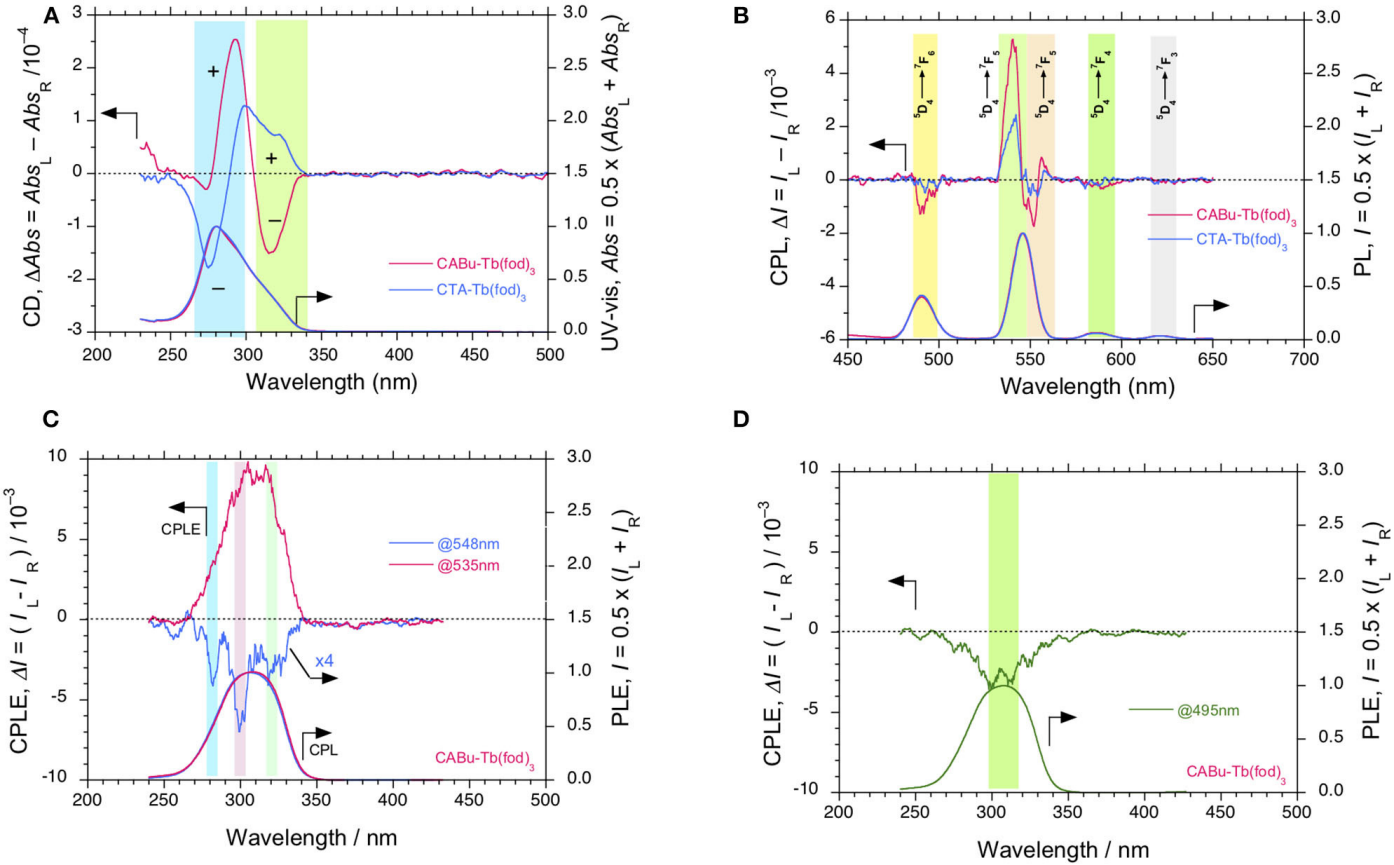
**(A)** Comparisons of CD and UV-visible and **(B)** CPL and PL spectra (excited at 310 nm) of Tb(fod)_3_ in **CTA** and **CABu** films, respectively. The cellulose films and Tb(fod)_3_ were hybridized at a weight ratio of 20 mg/10 mg from chloroform solution. **(C)** CPLE and PLE spectra of Tb(fod)_3_ monitored at 535 nm and 548 nm in **CABu** film. **(D)** CPLE and PLE spectra of Tb(fod)_3_ monitored at 495 nm in **CABu** film. The cellulose and Eu(fod)_3_ were hybridized at a weight ratio of 20 mg/10 mg in chloroform solution.

The normalized CD and UV-visible spectra of Tb(fod)_3_ in **CTA** and **CABu** films are compared in Figure 3A. For comparison, the original CD and UV-visible spectra of the films were given in Figure S13B, SM. Unlikely to the case of Eu(fod)_3_, trisignate profile at CD bands appeared at (–)-sign at 315 nm, (+)-sign at 292 nm and (–)-sign at 273 nm in **CABu** while (+)-sign at 322 nm, (+)-sign at 300 nm and (–)-sign at 275 nm in **CTA**. The apparent *g*_abs_ values at the first Cotton band of Tb(fod)_3_ in **CABu** and **CTA** films are −3.5 × 10^−4^ at 315 nm and +2.0 × 10^−4^ at 324 nm, respectively. **CABu** efficiently induced the Cotton CD band of Tb(fod)_3_ rather than **CTA**, similar to the case of Eu(fod)_3_.

The different CD inducibility of Tb(fod)_3_ between **CABu** and **CTA** reflects the corresponding CPL spectra at 4*f-*4*f* transitions of Tb(fod)_3_. Figure 3B shows the normalized CPL and PL spectra excited at 310 nm of Tb(fod)_3_ in **CTA** and **CABu** films. Obviously, three CPL bands are ascribed to ^5^${\text{D}}_{4}\to^{7}$F_6_ transition (490 nm) and ^5^${\text{D}}_{4}\to^{7}$F_5_ transitions (540 and 552 nm), respectively (Table 1). On the other hand, we did not detect any CPL bands at ^5^${\text{D}}_{4}\to^{7}$F_4_ transition (585 nm) and ^5^${\text{D}}_{4}\to^{7}$F_3_ transition (622 nm) clearly though the corresponding PL band was obvious. The *g*_lum_ values of Tb(fod)_3_ in **CABu** are −2.91 × 10^−3^ at ^5^${\text{D}}_{4}\to^{7}$F_6_ transition (490 nm), +7.82 × 10^−3^ at ^5^${\text{D}}_{4}\to^{7}$F_5_ transition (540 nm) and −1.75 × 10^−3^ at ^5^${\text{D}}_{4}\to^{7}$F_5_ transition (552 nm), respectively (Table 1). Those values in **CTA** weaken with −1.01 × 10^−3^ at ^5^${\text{D}}_{4}\to^{7}$F_6_ transition (490 nm), +3.53 × 10^−3^ at ^5^${\text{D}}_{4}\to^{7}$F_5_ transition (542 nm) and −0.68 × 10^−3^ at ^5^${\text{D}}_{4}\to^{7}$F_5_ transition (553 nm), respectively (Table 1). Although the origin of the differences in the *g*_abs_ at the first Cotton CD bands and *g*_lum_ values of CPL bands at 4*f-*4*f* transitions between Eu(fod)_3_ and Tb(fod)_3_ remains, **CABu** and **CTA** commonly induced clear CD and CPL bands.

Although the |*g*_lum_| values (0.35–0.78 × 10^−2^ at 540 nm, Table 1) at ^5^${\text{D}}_{4}\to^{7}$F_5_ transition of Tb(fod)_3_ in **CABu** and **CTA** films are comparable to those (0.83 × 10^−2^ at 542 nm) of Tb^III^(hfa)_3_ (hfa:hexafluoroacetonate) with chiral 4,12-bis(diphenylphosphino)-[2.2]-paracyclophane) (Taniguchi et al., 2019) and weaken by one order of magnitude compared to those (4–8 × 10^−2^ at ~540 nm) of Tb^III^ complexes coordinated with chiral bis(oxazolinyl)pyridine (Yuasa et al., 2011). The well-designed chiral ligands induce CPL-functionality to Tb^III^ complexes in solution more efficiently than solidified polysaccharide alkyl esters.

### Chirality Transfer Capability From Cellulose Alkyl Esters to Eu(dpm)_3_ and Tb(dpm)_3_

The dpm ligand is a symmetrical β-diketonate, in which two methyl groups of acetylacetonate are replaced by two electron-donating (ED) *tert*-butyl groups. Therefore, Tb(dpm)_3_ and Eu(dpm)_3_ can adopt a single *D*_3_-symmetrical configuration (Brittain and Richardson, 1977a; Brittain, 1980). In a recent paper (Jalilah et al., 2018) and the present work, we confirmed that Eu(fod)_3_ in α-pinene has shown clear CPL signals due to the presence of electron-withdrawing (EW) fluoroalkyl groups. Eu(dpm)_3_ in α-pinene shows no detectable CPL signals due to the lack of EW fluoroalkyl groups (Figure S14B, SM).

**Figure 4:**
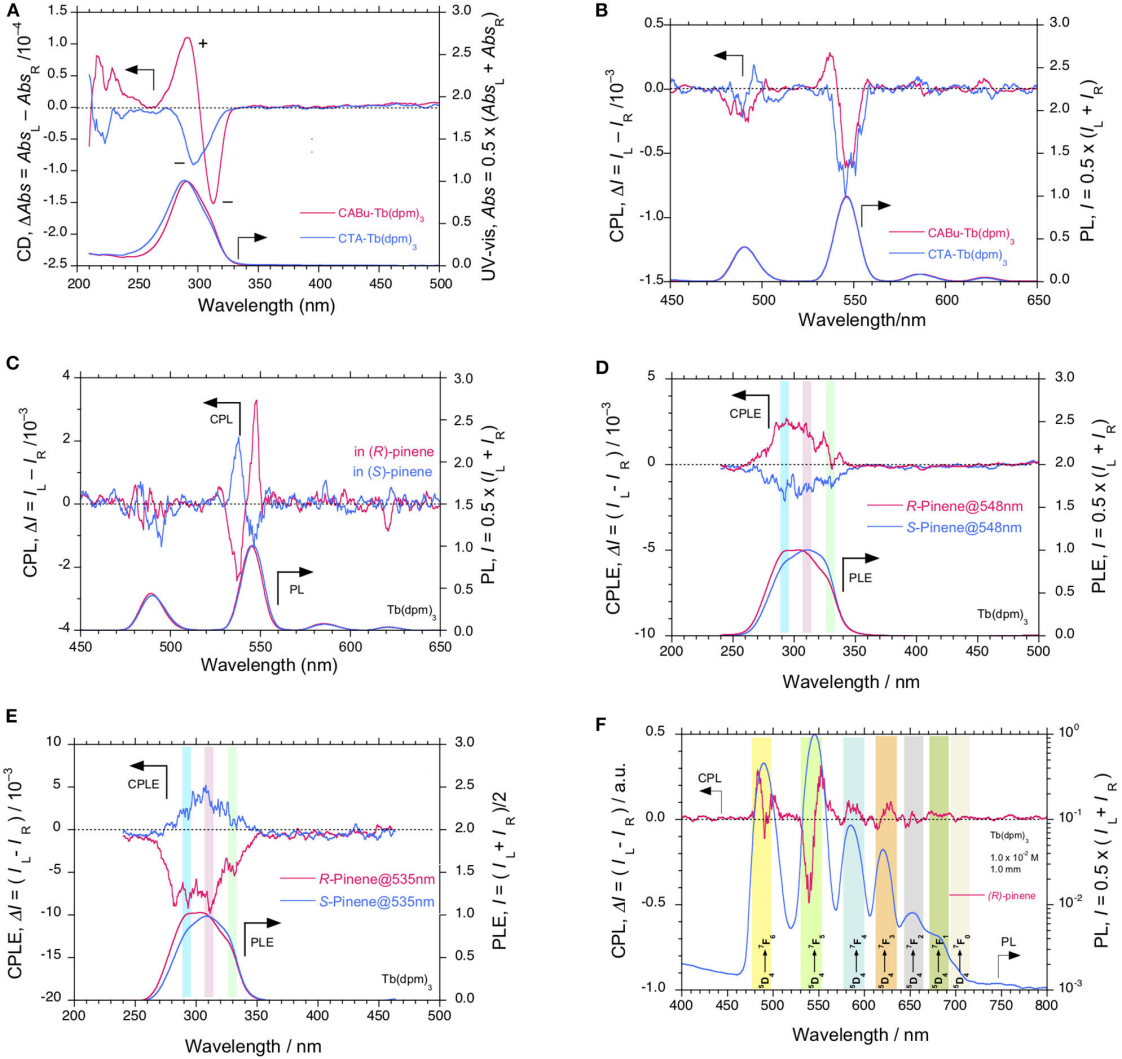
**(A)** Comparisons of CD and UV-visible and **(B)** CPL and PL spectra (excited at 310 nm) of Tb(dpm)_3_ in **CTA** and **CABu** films, respectively. **(C)** CPL and PL spectra (excited at 315 nm) of Tb(dpm)_3_ in α-pinene ([conc]_0_ = 10^−2^ M, pathlength 1.0 mm) and their CPLE and PLE spectra monitored at **(D)** 548 nm and **(E)** 535 nm, respectively. **(F)** PL (in log scale) with CPL spectra (excited at 320 nm) associated with 4*f-*4*f* transitions of Tb(dpm)_3_ in (*R*)-α-pinene. The cellulose films and Tb(dpm)_3_ were hybridized at a weight ratio of 20 mg/10 mg in THF solution.

Tb(dpm)_3_ in **CABu** and **CTA** films shows clear CD and UV-visible spectra in the range of 200 nm and 340 nm, as shown in Figure 4A. For comparison, the original CD and UV-visible spectra of the films are given in Figure S13C, SM. Tb(dpm)_3_ in **CABu** and **CTA** films, however, showed detectable but weak CPL spectra at 4*f-*4*f* transitions, as shown in Figure 4B. The *g*_lum_ values in **CABu** film are −0.53 × 10^−3^ at ^5^${\text{D}}_{4}\to^{7}$F_6_ (491 nm), +0.37 × 10^−3^ at ^5^${\text{D}}_{4}\to^{7}$F_5_ (537 nm), −0.59 × 10^−3^ at ^5^${\text{D}}_{4}\to^{7}$F_5_ (547 nm), while the *g*_lum_ values in **CTA** are −0.44 × 10^−3^ at ^5^${\text{D}}_{4}\to^{7}$F_6_ (489 nm) and −0.80 × 10^−3^ at ^5^${\text{D}}_{4}\to^{7}$F_5_ (547 nm) (Table 1). The absolute magnitudes of *g*_lum_ values with dpm ligands greatly diminished compared to Tb^III^ complexes with fod ligands. Tb(dpm)_3_ and Eu(dpm)_3_ showed different behaviors toward external chiral chemical perturbations regardless of the same fod and dpm as the ligands. Eu(dpm)_3_ in **CABu** and **CTA** did not demonstrate obvious CPL spectra although the corresponding PL spectra are evident (Figures S14A,B, SM).

We ascertained many times that there were no detectable CPL signals of Eu(dpm)_3_ in **CABu** films. This could be because Eu(dpm)_3_ is lack of EW-fluoroalkyl groups that can cause efficient chiral C–F/H–C interactions. Chiral *C*–*O*/*H*–*C* interactions seem not efficient to induce the chiral perturbation.

### Chirality Transfer Capability From Monosaccharide Permethyl Esters to Eu(fod)_3_ and Tb(fod)_3_

**Figure 5:**
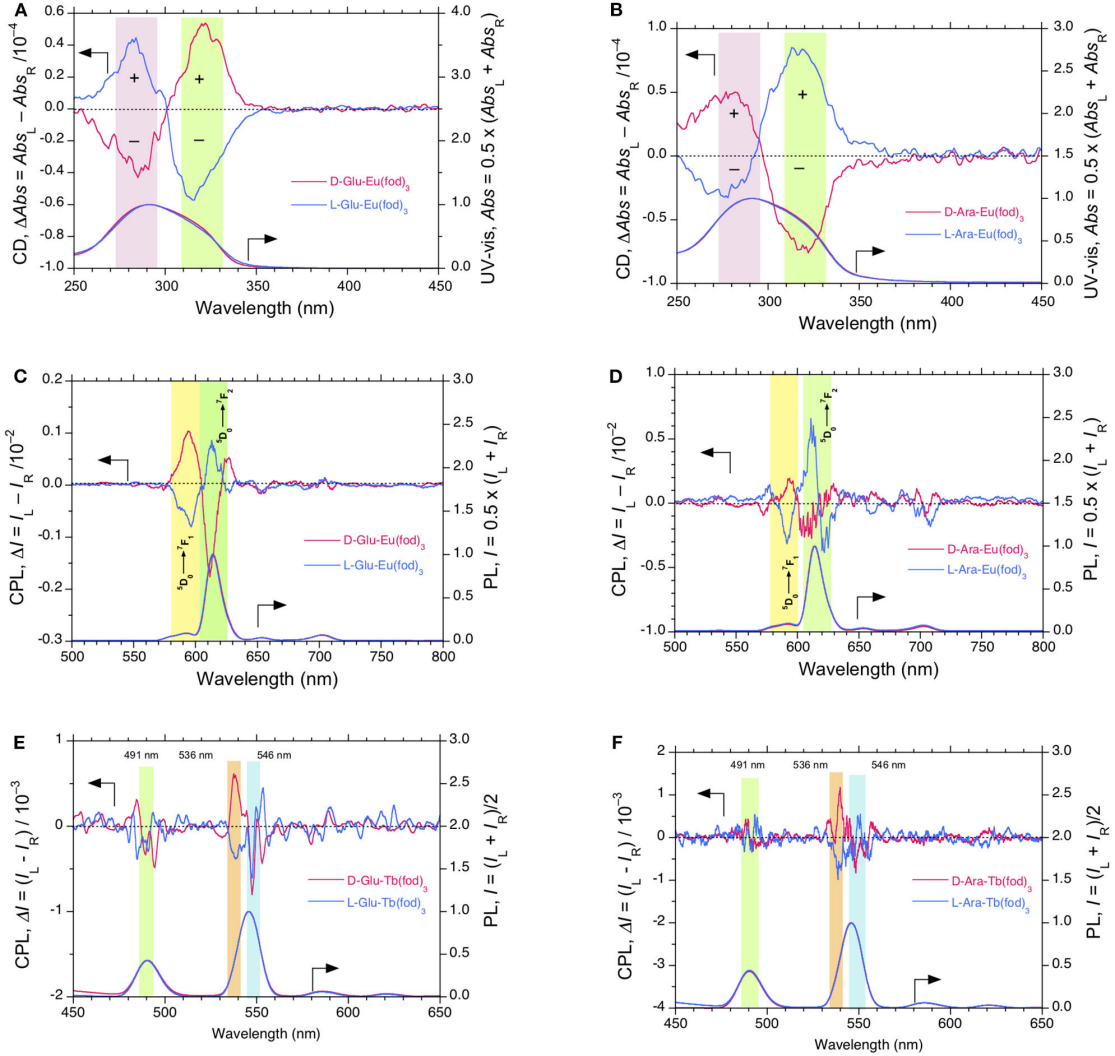
**(A)** CD and UV-visible spectra and **(C)** CPL and PL spectra (excited at 315 nm) of Eu(fod)_3_ in ***D*-**/***L*-Glu** films. **(B)** CD and UV-visible spectra and **(D)** CPL and PL spectra (excited at 315 nm) of Eu(fod)_3_ in ***D*-**/***L*-Ara** films. **(E)** CPL and PL spectra (excited at 315 nm) of Tb(fod)_3_ in ***D*-**/***L*-Glu** films. **(F)** CPL and PL spectra (excited at 310 nm) of Tb(fod)_3_ in ***D*-**/***L*-Ara** films. The saccharide ester and Eu(fod)_3_ and Tb(fod)_3_ were hybridized at a weight ratio of 20 mg/10 mg in chloroform solution.

Kipping and Pope found that preferential crystallization of *L-*NaClO_3_ in the presence of naturally occurring *D*-glucose and *D*-mannitol (Kipping and Pope, 1898). Currently, non-naturally occurring *L*-glucose is available commercially, although it is costly. *L*-(+)- and *D*-(–)-arabinose are also available, but the *L*-form is more abundant in nature than the *D*-form due to unknown reasons. To verify whether chirogenesis of Eu(fod)_3_ is solely determined by point chirality of monosaccharides, we measured CD and CPL spectra of Eu(fod)_3_ embedded in ***D*-**/***L*-Glu** and ***D*-**/***L*-Ara** films, as displayed in Figures 5A–D.

Firstly, CD and UV-visible spectra between Eu(fod)_3_ in ***D*-** and ***L*-Glu** films are compared in Figure 5A. The original CD and UV-visible spectra of the films are given in Figure S13D, SM. Eu(fod)_3_ showed nearly mirror-image bisignate CD bands, though the value of λ_ext_ at the first and second Cotton bands are considerably different from each other. The *g*_abs_ values of Eu(fod)_3_ in ***D-*Glu** film are +0.89 × 10^−4^ at 321 nm and −0.42 × 10^−4^ at 284 nm, while in ***L-*Glu** film, these values are −0.80 × 10^−4^ at 315 nm and +0.44 × 10^−4^ at 282 nm (Table 1).

CD and UV-visible spectra between Eu(fod)_3_ in ***D-***and ***L*-Ara** films are compared in Figure 5B. The original CD and UV-visible spectra of the films are given in Figure S13E, SM. Similarly, Eu(fod)_3_ shows nearly mirror-image bisignate CD bands, though the value of λ_ext_ at the first and second Cotton bands are subtly different. The *g*_abs_ values of Eu(fod)_3_ in ***D-*Ara** are −0.96 × 10^−4^ at 321 nm and +0.55 × 10^−4^ at 281 nm, respectively, while in ***L-*Glu** film, these values are +1.08 × 10^−4^ at 316 nm and −0.39 × 10^−4^ at 275 nm, respectively (Table 1).

Next, we compared CPL and PL spectra excited at 315 nm of Eu(fod)_3_ in ***D-***and ***L*-Glu** films, shown in Figure 5C. Eu(fod)_3_ displays nearly mirror-image trisignate CPL bands at 4*f-*4*f* transitions associated with emission wavelengths, though the absolute *g*_lum_ values at these transitions are somewhat different. The *g*_lum_ values in ***D-*Glu** are +1.05 × 10^−2^ at ^5^${\text{D}}_{0}\to^{7}$F_1_ (594 nm), −0.19 × 10^−2^ at ^5^${\text{D}}_{0}\to^{7}$F_2_ (612 nm) and +0.19 × 10^−2^ at ^5^${\text{D}}_{0}\to^{7}$F_2_ (626 nm), while in ***L-*Glu**, these values are −0.81 × 10^−2^ at ^5^${\text{D}}_{0}\to^{7}$F_1_ (596 nm), +0.08 × 10^−2^ at ^5^${\text{D}}_{0}\to^{7}$F_2_ (613 nm), and −0.04 × 10^−2^ at ^5^${\text{D}}_{0}\to^{7}$F_2_ (627 nm) (Table 1).

For comparison, CPL and PL spectra of Eu(fod)_3_ in ***D-***and ***L*-Ara** films were excited at 315 nm, and the results are presented in Figure 5D. Eu(fod)_3_ exhibits no longer mirror-image trisignate CPL bands at 4*f-*4*f* transitions, though the absolute *g*_lum_ values at these transitions weaken several times. The *g*_lum_ values in ***D-*Ara** are +0.19 x 10^−2^ at ^5^${\text{D}}_{0}\to^{7}$F_1_ (593 nm), −0.02 × 10^−2^ at ^5^${\text{D}}_{0}\to^{7}$F_2_ (607 nm), and +0.42 × 10^−2^ at ^5^${\text{D}}_{0}\to^{7}$F_2_ (630 nm), while in ***L-*Ara**, these values are −0.30 × 10^−2^ at ^5^${\text{D}}_{0}\to^{7}$F_1_ (591 nm), +0.06 × 10^−2^ at ^5^${\text{D}}_{0}\to^{7}$F_2_ (611 nm), and −0.72 × 10^−2^ at ^5^${\text{D}}_{0}\to^{7}$F_2_ (622 nm) (Table 1).

CPL and PL spectra of Tb(fod)_3_ excited at 315 nm in ***D-***/***L*-Glu** and ***D-***/***L*-Ara** films are given in Figures 5E,F, respectively. Regardless of **Glu** and **Ara**, although Tb(fod)_3_ shows very weak CPL bands at 4*f-*4*f* transitions, CPL signs are likely to depend on the chirality of **Glu** and **Ara**. Because the absolute *g*_lum_ values at these 4*f-*4*f* transitions considerably weaken, the *g*_lum_ values cannot be precisely evaluated.

Although *L*-cellulose is not available on earth, we can conclude that *D*-chirality of **CTA**, **CABu**, **Glu**, and **Ara** determines the (+)- and (–)-sign CPL characteristics of Eu(fod)_3_ at ^5^${\text{D}}_{0}\to^{7}$F_1_ and ^5^${\text{D}}_{0}\to^{7}$F_2_ transitions. Conversely, (–)- and (+)-signs at these transitions are from the *L*-chirality of **Glu** and **Ara**, although the inversion in the bisignate CD bands of Eu(fod)_3_ at 280–290 nm and 300–310 nm are considerably dependent on the nature of the alkyl groups of **CTA** and **CABu**. Similarly, *D*-chirality of **CTA**, **CABu**, **Glu**, and **Ara** determines the (+)- and (–)-sign CPL characteristics of Tb(fod)_3_ at two ^5^${\text{D}}_{4}\to^{7}$F_5_ transitions. Conversely, *L*-chirality of **Glu** and **Ara** determines the (–)- and (+)-signs at the transitions.

### Chirality Transfer Capability From α-Pinene to Eu(fod)_3_, Tb(fod)_3_, Eu(dpm)_3_, and Tb(dpm)_3_

Bicyclic terpenes, (1*S*)-/(1*R*)-α-pinene and (1*S*)-/(1*R*)-β-pinene, are chiral rigid hydrocarbons and are transparent in the near-UV and visible region. This feature is beneficial to gain an insight into higher energy states responsible for CPL transitions by measuring CPLE spectra monitoring at a specific CPL wavelength. Actually, the chirality of α*-*/β-pinenes is transferred to achiral or racemic Eu(fod)_3_ with considerably large *g*_lum_ values at 4*f-*4*f* transitions, that were verified by CPLE spectra (Jalilah et al., 2018). This result prompted us to test whether Tb(fod)_3_ in α-pinene reveals CPL signals, although Tb(fod)_3_ did not show clear CPL signals because of an unknown reason.

On the other hand, Tb(dpm)_3_ dissolved in α-pinene shows clear CPL signals, in which signs are determined solely by the chirality of the α-pinene (Figure 4C). The *g*_lum_ values in (1*S*)- α-pinene are +4.35 × 10^−3^ at ^5^${\text{D}}_{4}\to^{7}$F_5_ (537 nm) and −1.28 × 10^−3^ at ^5^${\text{D}}_{4}\to^{7}$F_5_ (547 nm), while in (1*R*)-α-pinene are −4.94 × 10^−3^ at ^5^${\text{D}}_{4}\to^{7}$F_5_ (537 nm) and +3.44 × 10^−3^ at ^5^${\text{D}}_{4}\to^{7}$F_5_ (548 nm), though the CPL bands at ^5^${\text{D}}_{4}\to^{7}$F_6_ (490 nm) are not obvious (Table 1). Evidently, (+)- and (–)-sign CPL characteristics of Eu(fod)_3_ at ^5^${\text{D}}_{0}\to^{7}$F_1_/^5^${\text{D}}_{0}\to^{7}$F_2_ transitions and Tb(dpm)_3_ at two ^5^${\text{D}}_{4}\to^{7}$F_5_ transitions are determined by *S-* and *R-*chirality of α-pinene, respectively. In comparison to Tb(dpm)_3_, Eu(dpm)_3_ in α-pinene has shown no detectable CPL signals (Figure S14C, SM). From PL spectrum of Tb(dpm)_3_ in (*R*)-α-pinene excited at 320 nm associated with the corresponding CPL signals ranging of 500 and 800 nm (Figure 4F), seven characteristic 4*f*-4*f* transitions were assigned to ^5^${\text{D}}_{4}\to^{7}$F_6_ (490.0 nm, 20408 cm^−1^), ^5^${\text{D}}_{4}\to^{7}$F_5_ (545.5 nm, 18330 cm^−1^), ^5^${\text{D}}_{4}\to^{7}$F_4_ (584.5 nm, 17,109 cm^−1^), 5${\text{D}}_{4}\to^{7}$F_3_ (620.0 nm, 16129 cm^−1^), ^5^${\text{D}}_{4}\to^{7}$F_2_ (652.5 nm, 15,326 cm^−1^), ^5^${\text{D}}_{4}\to^{7}$F_1_ (682.0 nm, 14,663 cm^−1^), and ^5^${\text{D}}_{4}\to^{7}$F_0_ (702.5 nm, 14,235 cm^−1^) (Fulgêncio et al., 2012; de Queiroz et al., 2015; Xue et al., 2015; Yang et al., 2017).

We further verified these CPL signals of Tb(dpm)_3_ by the broad CPLE spectra centered at ~300 nm associated with the corresponding PLE spectra with several shoulders, as marked by blue, pink, and green bars in Figures 4D,E. The signs of the CPLE spectra depend on the signs at monitor wavelengths (535 and 548 nm) and α-pinene chirality; (+)-sign in CPLE spectrum is identical to (+)-sign CPL signal at 546 nm in (*R*)-pinene, while (+)-sign in CPLE spectrum is the same of (+)-sign CPL signal at 535 nm in (*S*)-pinene. The broad CPLE/PLE spectra may arise from at least three different origins of *n*-π^*^/^1^π-^3^π^*^ transitions of three dpm ligands associated with high energy levels (e.g., ^5^D_1_ and ^5^D_2_) of Tb^III^ (Fulgêncio et al., 2012; de Queiroz et al., 2015; Xue et al., 2015). A similar tendency can be seen in the broad CPLE/PLE spectra of Tb(fod)_3_ in **CABu**, as marked in blue, pink, and green bars in Figures 3C,D.

Unresolved factors between Tb^III^ and Eu^III^ associated with ligands (fod and dpm) and chiral matrices (α-pinene and monosaccharide alkyl esters) are other critical parameters to generate CPL signals and boost *g*_lum_ characteristics. Although the inherent nature of the differences between Tb^III^ and Eu^III^ is unresolved, we assume that the interactions of C-*H*/π between C(δ–)-H(δ+) bonds at dpm of Tb(dpm)_3_ and C(δ+)-C(δ–) double bond of α-pinene are more crucial while the C-*F*/*H*-C interactions between C(δ+)-F(δ–) bonds of fod ligands and H(δ+)-C(δ–) bonds of α-pinene are crucial (Jalilah et al., 2018).

### Intermolecular Interactions Between Eu(fod)_3_ and CTA/Glu by Solid-State ^13^C{^1^H}-NMR and Solution ^1^H/^19^F-NMR Spectra

We do not yet know what kinds of intermolecular noncovalent interactions exist between the lanthanide tris(β-diketonate) and the poly-/monosaccharide alkyl esters. Among lanthanide tris(β-diketonate)s, we chose Eu(fod)_3_ for simplicity and excellent solubility in CDCl_3_. The three fod ligands have ^1^H, ^19^F, and ^13^C-NMR active elements to discuss the possible interactions. Additionally, we chose **CTA** and ***D*-/*L*-Glu** for simplicity in these solid-state (ss)-^13^C-NMR and solution ^1^H-/^19^F-NMR spectra. According to the hard-soft-acid-base theory proposed by Pearson (Pearson, 1963), the lone pairs of the hard base O atom(s) of ester groups can coordinate with the hard acid Eu^III^ of Eu(fod)_3_. A marked chemical shift in ^13^C-NMR spectra of ester (*C*=O and O) and ethereal *C*–O–*C* atoms was expected.

**Figure 6:**
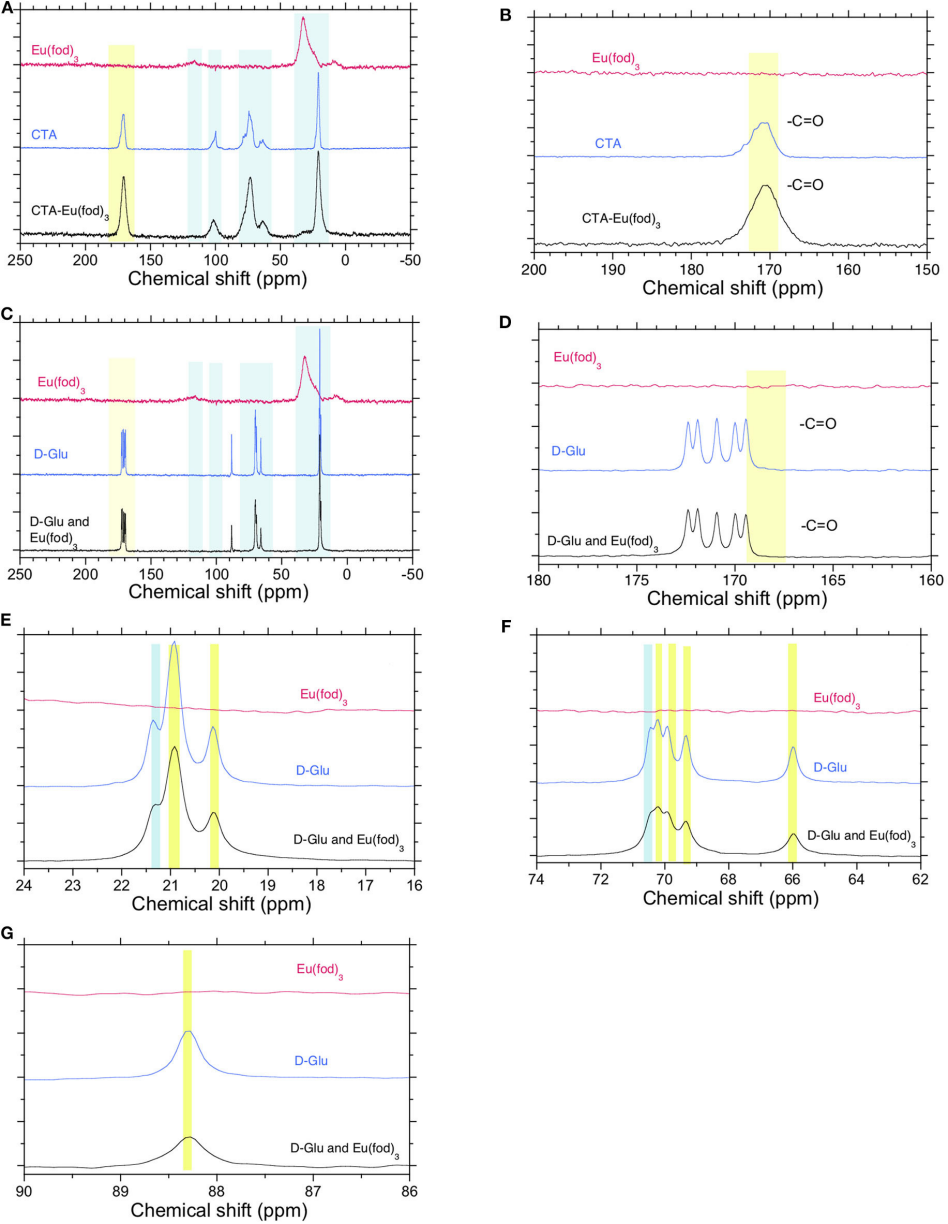
**(A)** Solid-state (ss)-CP/MAS-^13^C{^1^H}-NMR spectra of Eu(fod)_3_, **CTA**, and a mixture of Eu(fod)_3_ and **CTA** in 1/1 (w/w). **(B)** Its magnified spectrum in the range of 150–200 ppm. **(C)** ss-^13^C{^1^H}-NMR spectra of Eu(fod)_3_, ***D*-Glu**, and a mixture of Eu(fod)_3_ and ***D*-Glu** in 1/1 (w/w) **(D,E)**. Their magnified spectra of methyl groups in ***D*-Glu** in the range of 150–200 ppm and 16–24 ppm **(F,G)**. Their magnified spectra of ***D*-**glucose ring in the range of 62–90 ppm. A broad ^13^C-NMR signal at 130 ppm (Figures 5A,C) was attributable to β-diketonate.

The ss-^13^C{^1^H}-NMR spectra of Eu(fod)_3_, **CTA**, and a mixture of Eu(fod)_3_ and **CTA** in 1/1 (w/w) are compared in Figures 6A,B. The ss-^13^C-NMR spectra of the Eu(fod)_3_-**CTA** mixture are merely a convolution of those of **CTA** and Eu(fod)_3_. Any noticeable chemical shift in the ^13^C-NMR spectra was not seen. ^13^C-NMR chemical shift at ~170 ppm due to O=*C*-O of **CTA** was unchanged after mixing with Eu(fod)_3_.

The ss-^13^C{^1^H}-NMR spectra of Eu(fod)_3_, ***D*-Glu**, and a mixture of Eu(fod)_3_ and ***D*-Glu** in 1/1 (w/w) are compared in Figures 6C–G. Similarly, the ss-^13^C-NMR spectra of the Eu(fod)_3_-***D*-Glu** mixture are merely a convolution of those of ***D*-Glu** and Eu(fod)_3_. Any remarkable chemical shift in the ^13^C-NMR spectra was not seen. Even five well-resolved O=*C*-O peaks of ***D*-Glu** pentamethyl ester ranging of ~169 ppm and ~173 ppm showed no detectable chemical shifts (Figure 6D). Similarly, no noticeable chemical shifts of three well-resolved methyl groups ranging from ~19 ppm to ~22 ppm were observed (Figure 6E). Among five well-resolved ^13^C peaks assignable to O = *C*–O of five esters, *C*–O–*C* pyranose ring ranging from ~64 to ~72 ppm and at ~83 ppm does not show remarkable chemical shifts of *D*-glucose ring. These ss-^13^C{^1^H}-NMR data led us to the conclusion that any O atom(s) of glucose ring and ester moieties do not coordinate directly to Eu^III^ ions.

**Figure 7:**
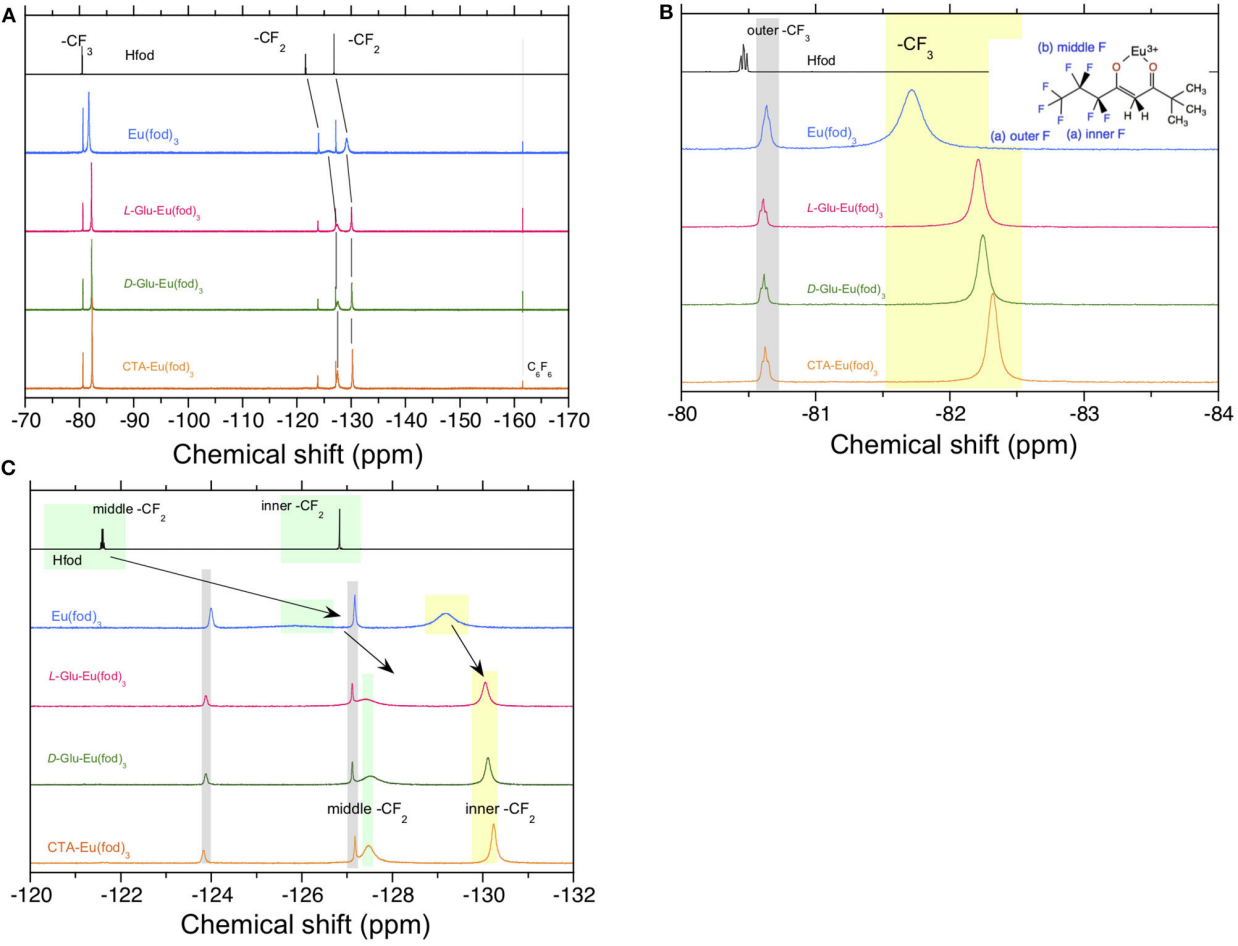
^19^F-NMR spectra in CDCl_3_ at room temperature of Hfod, Eu(fod)_3_, Eu(fod)_3_ with ***L*-Glu**, Eu(fod)_3_ with ***D*-Glu**, and Eu(fod)_3_ with **CTA** in the range of **(A)** −170 ppm and −70 ppm, **(B)** −84 ppm and −80 ppm, and **(C)** −132 ppm and −120 ppm. Because of the limited solubility of **CTA** (20 mg) in 0.6 mL of CDCl_3_, 10 mg of **CTA** and 10 mg of Eu(fod)_3_ were co-dissolved in 0.6 mL of CDCl_3_.

On the other hand, alterations in the chemical shifts of the solution ^19^F-NMR spectra of Eu(fod)_3_ in the absence and presence of **CTA**, ***D*-Glu**, and ***L*-Glu** in CDCl_3_ are apparent, as shown in Figures 7A–C. The outer CF_3_ signal of Eu(fod)_3_ in CDCl_3_ resonates at −81.73 ppm as a single peak, indicating C_3_-symmetrical geometry (Figure 7B). The single peak resonates at −82.21 ppm and −82.26 ppm in the absence and presence of ***L*-Glu** and ***D*-Glu**, respectively, and shifts upfield by 0.48 ppm and 0.53 ppm, respectively (Figure 7B). In the presence of **CTA**, the CF_3_ signal resonates at −82.31 ppm and shifts upfield by 0.58 ppm (Figure 7B). Although the middle CF_2_ signal of fod ligand resonates broadly at −126 ppm, in the presence of ***L*-Glu** and ***D*-Glu**, this signal resonates at −127.4 ppm and −127.5 ppm, that is shifted upfield by 1.4 ppm and 1.5 ppm, respectively (Figure 7C). Similarly, the middle CF_2_ peak at −127.5 ppm shifts upfield by 1.5 ppm in the presence of **CTA** (Figure 7C). The inner CF_2_ of fod shows a broad resonance at −129.2 ppm, but this signal appears at −130.1 ppm in the presence of ***L*-** and ***D*-Glu** and shifts upfield by 0.9 ppm (Figure 7C). Similarly, the signal appears at −130.3 ppm in the presence of **CTA** and shifts upfield by 1.1 ppm. These upfield shifts in the ^19^F-NMR spectra indicate intermolecular interactions between the F atoms and **CTA**, ***L*-Glu**, and ***D*-Glu**, possibly, C-*F*(δ–) (of the three fod ligands)/*H*(δ+)-C (of **CTA** and **Glu**) interactions.

It is thus evident that there were no marked alterations in ss-^13^C-NMR spectra between CPL-inactive and CPL-active Eu(fod)_3_ in **CTA**, ***L*-Glu**, and ***D*-Glu**, while the remarkable upfield chemical shifts in ^19^F-NMR of fod ligands in dilute CDCl_3_ solution was distinct. These characteristics should arise from outer or second-sphere effects perturbed by chiral chemicals that are non-coordinating to Eu^III^, the so-called 'Pfeiffer effect'. The multiple H(δ+)-C(δ–) bonds of chiral chemical species (**CTA**, ***D*-/*L*-Glu**, possibly, ***D*-/*L*-Ara** and **CABu**) interact with multiple F(δ–)-C(δ+) bonds of the three fod ligands but do not directly coordinate with Eu^III^.

**Figure 8:**
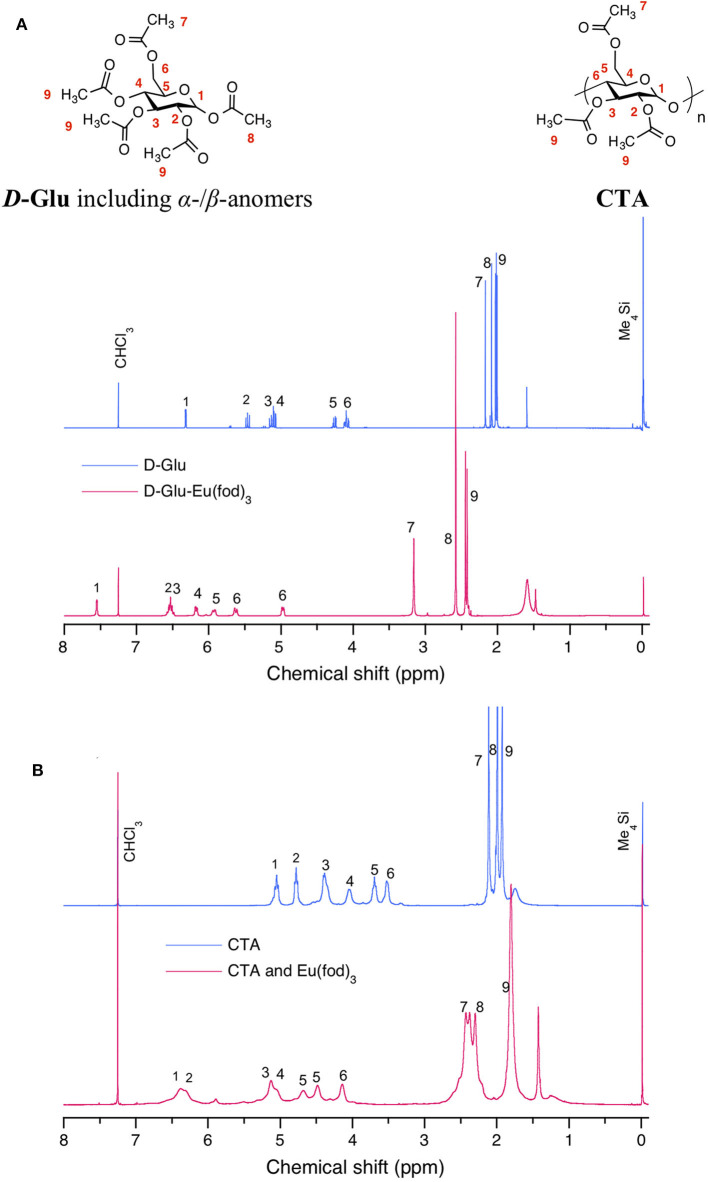
Comparison of ^1^H-NMR spectra in CDCl_3_ at room temperature between **(A)** ***D*-Glu** (blue) and Eu(fod)_3_ with ***D*-Glu** (red) and between **(B) CTA** (blue) and Eu(fod)_3_ with **CTA** (red). Sharper and broader ^1^H-NMR peaks at ~1.5 ppm are assumed to be free water in CDCl_3_ and bounded waters at Eu(fod)_3_, respectively. Although 20 mg of ***D*-Glu** and 10 mg of Eu(fod)_3_ were able to co-dissolve in 0.6 mL of CDCl_3_, 10 mg of **CTA** and 10 mg of Eu(fod)_3_ were co-dissolved in 0.6 mL of CDCl_3_ because of the limited solubility of 20 mg **CTA** in 0.6 ml of CDCl_3_.

The noticeable downfield chemical shifts in ^1^H-NMR spectra of ***D*-Glu** and **CTA** support the postulated C(δ+)-*F*(δ–) (of fod) and *H*(δ+)-C(δ–) (of **CTA** and ***D*-/L-Glu**) interaction. Figure 8A displays the changes in ^1^H-NMR spectra of ***D*-Glu** in the absence and presence of Eu(fod)_3_. All protons are assigned in the inset of the Figure. The degree of the downfield shifts is summarized in Table 2. Notably, protons H1, H3, H5, H6 shift downfield significantly and protons H2, H4, H6 (one of two), H7 also considerably shift downfield. We noticed that H1, H3, H5 are the glucose ring protons and H6 is the CH_2_ attached to C5 carbon. Possibly, Eu(fod)_3_ molecule is placed on top of the *D*-glucose ring. All H1–H7 protons are close to Eu(fod)_3_ molecule with the help of multiple C(δ+)-*F*(δ–)/*H*(δ+)-C(δ–) and C(δ–)-*H*(δ+)/*O*(δ–)-C(δ+) interactions.

**Table 2:** Comparison of chemical shifts in the ^1^H-NMR spectra in CDCl_3_ at room temperature between (a) *D*-Glu without and with Eu(fod)_3_ and between (b) CTA without and with Eu(fod)_3_, whereas (+)-sign stands for the downfield shift.

	**1**	**2**	**3**	**4**	**5**	**6**	**7**	**8**	**9**
*D*-Glu	6.31	5.45	5.14	5.09	4.26	4.09	2.17	2.08	2.01
Eu(fod)_3_ in *D*-Glu	7.55	6.53	6.53	6.17	5.93	5.61, 4.99	3.16	2.58	2.44, 2.42
Relative shifts of ^1^H-NMR	+1.24	+1.08	+1.39	+1.08	+1.67	+1.52, +0.90	+0.99	+0.50	+0.41, +0.43
CTA	5.05	4.78	4.37	4.04	3.70	3.52	2.10	1.99	1.92
Eu(fod)_3_ in CTA	6.38	6.31	5.12	5.05	4.67, 4.49	4.13	2.40	2.31	1.81
Relative shifts of ^1^H-NMR	+1.33	+1.53	+0.75	+1.01	+0.93, +0.79	+0.61	+0.30	+0.32	−0.11

Figure 8B exhibits similar changes in ^1^H-NMR spectra of **CTA** in the absence and presence of Eu(fod)_3_. Protons are assigned in the insets of the figure. The degree of downfield shifts is summarized in Table 2. Notably, protons H1 and H2 shift downfield greatly and protons H3, H4, H5 also shift considerably downfield. H1, H2, H3, and H4 are the glucose ring protons and H5 is the CH_2_ attached to C4 carbon. Similarly, Eu(fod)_3_ molecule is placed on top of the *D*-glucose ring of **CTA**. All H1–H5 protons are close to Eu(fod)_3_ molecule with the help of multiple C(δ+)–*F*(δ–)/*H*(δ+)-C(δ–) and C(δ–)-*H*(δ+)/*O*(δ–)-C(δ+) interactions.

For comparison, FT-IR spectra between Eu(fod)_3_, ***D*-Glu**, and Eu(fod)_3_ mixed with ***D*-Glu** in the ranges of 2,700 and 3,700 cm^−1^, 2,800 and 3,100 cm^−1^, and 1,000 and 2,000 cm^−1^ are shown in Figures S15A–C, SM. We observed no noticeable frequency shifts in ν(C-H) at 2,850–3,000 cm^−1^ and ν(C-F) at 1,250–1,050 cm^−1^. Since the postulated C-*F*/*H*-C and C-*H*/*O*-C interactions are very weak, the resulting frequency shifts might be minimal, possibly, within 10 cm^−1^. One ν^as^(C-H) at 2973 cm^−1^ and ν^s^(C-H) at 2873 cm^−1^ due to methyl groups of fod in the absence of ***D*-Glu** shift to lower frequencies at 2967 cm^−1^ by 7 cm^−1^ and 2871 by 2 cm^−1^, respectively (Figure S15B, SM). These small shifts may be the consequence of the C-*H*/*O*-C interactions. On the other hand, ν(C=O) at ~1,750 cm^−1^ characteristic of five ester group of ***D*-Glu** does not coordinate to Eu^III^ directly because there are no noticeable frequency shifts (Figure S15C, SM). Although, in the absence of ***D*-Glu**, Eu(fod)_3_ has one broad and one shoulder ν(C=O) band at 1621 cm^−1^ and 1594 cm^−1^ due to the β-diketonate, in the presence of ***D*-Glu**, the shoulder ν(C=O) may disappear and merge to 1621 cm^−1^ or shift to 1642 cm^−1^ due to specific alteration of β-diketonates. Other frequency shifts such as ν(C–O–C) at ~1200 cm^−1^ and 1150 cm^−1^ of the ester groups and glucose rings of ***D*-Glu** are not apparent because of significant overlapping with other intense ν(C–F) bands. The C-*F*/*H*-C interactions are not obvious due to the significant overlapping.

Figures S16A–C, SM compare the FT-IR spectra between Eu(fod)_3_, **CABu**, and Eu(fod)_3_ with **CABu** in the ranges of 2,700 and 3,700 cm^−1^, 2,800 and 3,100 cm^−1^, and 1,000 and 2,000 cm^−1^. Similarly, minimal frequency shifts in ν(C-H) at 2,850–3,000 cm^−1^ can be seen due to the postulated C-*F*/*H*-C and C-*H*/*O*-C interactions (Figure S16B, SM). The ν^as^(C-H) band at 2,973 cm^−1^ and ν^s^(C-H) at 2,873 cm^−1^ of fod methyl groups in the absence of **CABu** shift to lower frequencies at 2,967 cm^−1^ by 7 cm^−1^ and conversely higher frequency of 2,878 cm^−1^ by 5 cm^−1^, respectively (Figure S16B, SM). These small shifts may arise from the C-*H*/*O*-C interactions. On the other hand, a broad ν(C=O) band at ~1,630 cm^−1^ characteristic of the β-diketonate split into two ν(C=O) bands at 1,643 cm^−1^ and 1,623 cm^−1^, suggesting specific structural alterations of the β-diketonates by the ester groups and/or ethers of **CABu**. However, no noticeable frequency shifts of ν(C–O–C) at ~1,200 cm^−1^ and 1,150 cm^−1^ of the ester groups of **CABu** are not seen because of the spectral overlapping with the intense ν(C–F) bands (Figure S16C, SM).

### Photodynamics of Eu(fod)_3_, Eu(dpm)_3_, and Tb(dpm)_3_ in CTA and CABu Films

Lifetimes of Eu(fod)_3_, Eu(dpm)_3_, and Tb(dpm)_3_ species embedded to **CTA** and **CABu** films excited at an N_2_ pulsed laser 337.1 nm are summarized in Table 3 based on decay curves (semilog and linear plots) of these emitters (Figures S17A–K, SM). The decay times (τ) of these emitters in **CABu** are somewhat long by the magnitude of 15–56 % compared to those in **CTA**. Possibly, these emitters have differently interacted with **CTA** and **CABu**, that depends on the nature of alkyl esters. Alternatively, regardless of **CTA** and **CABu**, the values of τ belong to in the order of Eu(fod)_3_, Eu(dpm)_3_, and Tb(dpm)_3_, depending on the nature of lanthanides and ligands.

**Table 3:** Lifetimes of photoexcited Eu(fod)_3_, Eu(dpm)_3_, and Tb(dpm)_3_ embedded to CTA and CABu films^a^.

**Lanthanide complexes**	**Eu(dpm)_**3**_**	**Eu(dpm)_**3**_**	**Eu(fod)_**3**_**	**Eu(fod)_**3**_**	**Tb(dpm)_**3**_**	**Tb(dpm)_**3**_**
Poly(*D*-saccharide)s	**CTA**	**CABu**	**CTA**	**CABu**	**CTA**	**CABu**
τ in msec	0.24^b^	0.29^b^	0.44^b^	0.50^b^	0.59^c^	0.92^c^

a *Pulsed N_2_ laser, 337.1 nm, 10 Hz repetition, Grating 150 lines per mm, slit width 100 μm*.

a *At room temperature, detected at 615 nm (collected from 610 to 620 nm)*,

c *detected at 546 nm (collected from 542 to 551 nm)*.

### Mulliken Charges of Sc^III^ Tris(β-diketonate) as Models of Eu^III^/Tb^III^ Tris(β-diketonate), *D*-Glu and *D*-Glu Dimer as a Model of CTA Obtained With MP2 (6-311G) Calculation

**Figure 9:**
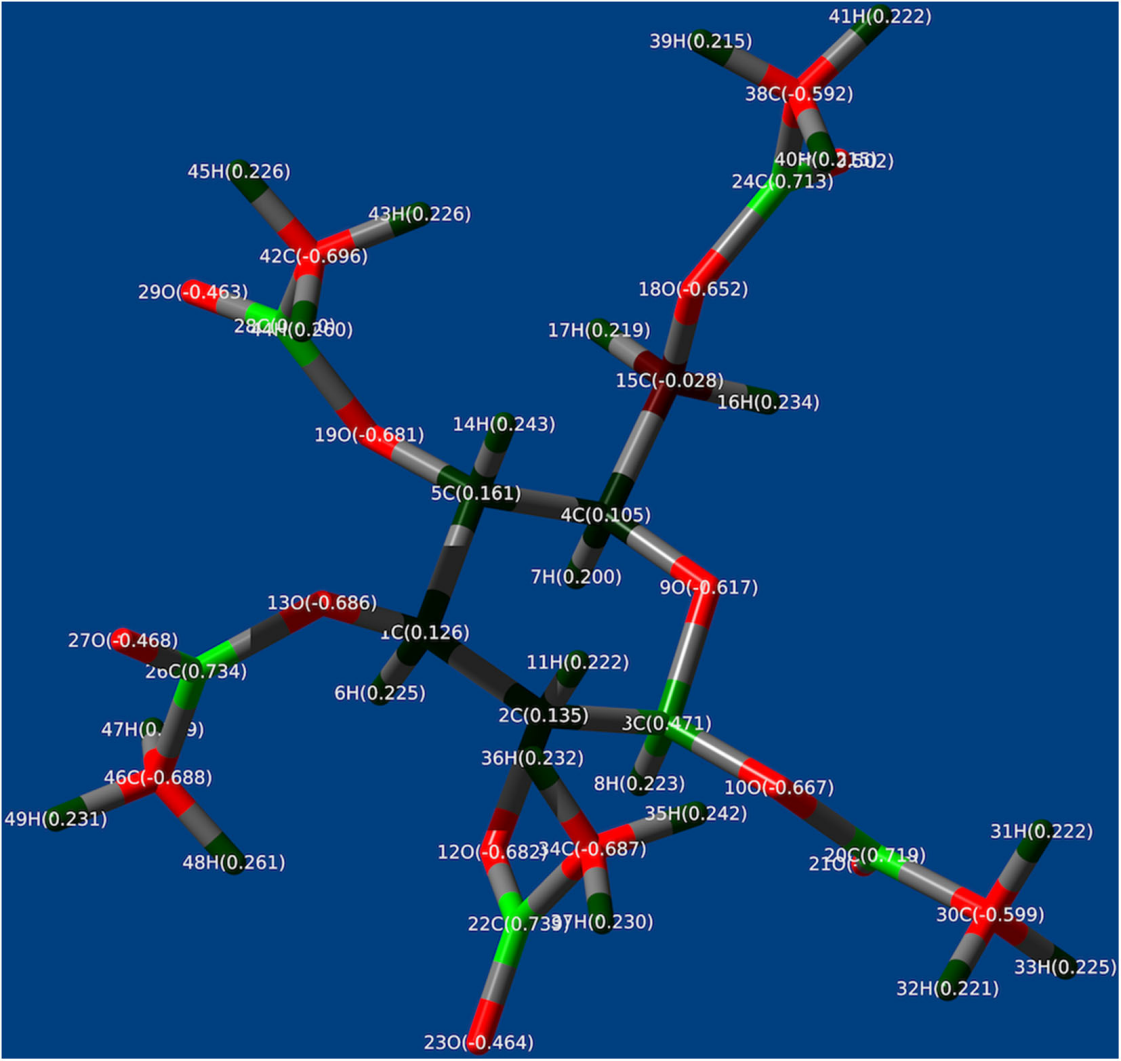
The Mulliken charges of ***D*-Glu** obtained with MP2 (6-311 G basis set).

To theoretically discuss possible intermolecular interactions, we calculated Mulliken charges (Mulliken, 1955) by the Møller–Plesset second-order perturbation theory (MP2) (Møller and Plesset, 1934; Head-Gordon et al., 1988) (6-311G basis set) method of the model compounds optimized by MM (UFF force filed), followed by DFT [6-31G(d)] methods (Frisch et al., 2013). Time-consuming MP2 calculation allows for reliable Mulliken charges compared to DFT calculation. Figure 9 and Figure S18, SM display the numbering for all the atoms in the ***D*-Glu** and ***D*-Glu dimer** as a model of **CTA**. All the peripheral hydrogen atoms of ***D*-Glu** and ***D*-Glu dimer** show positive Mulliken charges ranging from +0.215 to +0.261. The *O*=C atoms of methyl esters have substantial negative Mulliken charges of −0.464. We observed that the *O*–C atoms of methyl esters and the C–*O*–C atom in pyranose rings and C–*O*–C linkage between two pyranose rings show more substantial negative Mulliken charges ranging from −0.652 to −0.690, respectively. When C(δ–)–*H*(δ+) bond of the ligands (fod and dpm) feels the force of oxygen atoms, the C(δ+)–*O*(δ–)–C(δ+) and C(δ+)–*O*(δ–)–C(δ+)=O(δ–) bonds are more crucial than *O*(δ–)=C(δ+) bond of esters, leading to (ligand, C–*H*)/*O* (***D*-Glu**, possibly, **CTA**, **CABu**, **Glu**, and **Ara**) interactions.

In the previous paper (Jalilah et al., 2018), we proposed that the surplus charge neutralization obtained with Mulliken charges is a driving force of attractive forces between several ligands in the lanthanide complexes and CPL-inducible chiral substances. The fluorine atoms of Sc(fod)_3_ have negative Mulliken charges ranging from −0.341 to −0.373; conversely, the hydrogen atoms of Sc(fod)_3_ and Sc(dpm)_3_ have positive Mulliken charges ranging from +0.153 to +0.193.

The Mulliken charge neutralization between multiple (δ–) fluorines of fod and multiple (δ+) hydrogens of ***D*-Glu** and the saccharides is possible. Total surplus charge between C(δ+)-*F*(δ–) (fod) and *H*(δ+)-C(δ–) (saccharides, in this work) is that 3 × (−0.35) (*F*_3_C) + (4~5) × (+0.20 ~ +0.24) (*H*_3_C or *H*_2_C) = −0.05 ~ −0.09. Additionally, the Mulliken charge neutralization between multiple (δ+) hydrogens of the ligands (fod and dpm) and multiple (δ–) oxygen atoms of the saccharides are possible. Total surplus charge between (fod or dpm) C(δ–)-*H*(δ+)and *O*(δ–)-C(δ+) (saccharides) is that (4) × (+0.17) (*H*-C) + (1) × (−0.62 ~ −0.69) (*O*-C or *O*-C=O) = +0.06 ~ −0.01. Moreover, total surplus charge between (dpm and fod) C(δ–)-*H*(δ+) and *C*(δ–)=C(δ+) (α-pinene) is that (1) × (+0.17) (*H*-C) + (1) × (−0.18) (*C*=C) = −0.01; Tb(dpm)_3_ is a dominant factor, while Eu(dpm)_3_ is unimportant. However, it is reasonably assumed that the same Mulliken charges between multiple (δ+) hydrogen atoms of ligands (fod and dpm) and multiple (δ+) hydrogen atoms of the saccharides should cause repulsive interactions.

### The Degree of Chirogenesis and the Pfeiffer Effects

Since the serendipitous finding by an anomaly in an optical rotation of chiral substances in the presence of optically inactive labile metal ions in aqueous solutions (Pfeiffer and Quehl, 1931, 1932), the chirogenesis in the GS and ES from optically inactive labile metal complexes induced by chiral additives has been often appeared in the titles of several papers in the past and currently: e.g., Pfeiffer effect (Kirschner and Ahmad, 1968; Mayer and Brasted, 1973; Schipper, 1978; Brittain, 1982, 1984; Kirschner and Bakkar, 1982; Lunkley et al., 2018); outer-sphere coordination and complexation (Mason and Norman, 1965; Madaras and Brittain, 1980; Kirschner and Bakkar, 1982); second-sphere coordination (Colquhoun et al., 1986). Pfeiffer effect and/or outer-sphere/second-sphere coordination are mainly investigated in their solution states of the metal complexes.

An equilibrium shift from dynamic racemic mixtures (Δ :Λ = 50/50) of labile metal complexes is responsible for the Pfeiffer effect and chirogenesis by outer-sphere/second-sphere coordination. A barrier height of racemization should be rather small to permit dynamic racemization at ambient temperatures. In 1975, Schipper theoretically discussed chemical discrimination between racemic substances (A' and A”) and chiral substance B surrounded by achiral solvent (Schipper, 1975) as a model of the Pfeiffer effect. In the hypothetical system, an exothermic enthalpic gain Δ_h_/*T* is acquired to compensate an entropic loss Δ_s_. The long-range interactions in dynamically dissociate system is needed to overcome thermal fluctuation *k*_B_*T*.

To quantitatively discuss the degree of chirogenesis in six-coordinate labile lanthanide tris(β-diketonate) led by chiral additives in solution, we compare four solution PL spectra of Eu(fod)_3_ itself (10 mg, 0.8 × 10^−2^ M, red line) and in the presence of ***D*-Glu** (20 mg, 4 × 10^−2^ M, blue line), ***L*-Glu** (20 mg, 4 × 10^−2^ M, green line), and **CTA** (10 mg, 3 × 10^−2^ M, black line) in 1.2 mL of CDCl_3_ (Figure S19, SM). These CDCl_3_ solutions were used prior to measurements of ^19^F- and ^1^H-NMR spectra (Figure 7). Among ^5^D_0_-^5^F_*J*_ (*J* = 0,1,2,3,4) transitions, ^5^D_0_-^5^F_1_ transition at 594 nm is known to be susceptible to alterations in molecular symmetry and geometry coordinated with external ligands. Our previous investigation suggests that Eu(fod)_3_ is likely to adopt a facial *C*_3_-symmetrical structure and exists as a mixture of labile Δ- and Λ-isomers in solutions (Jalilah et al., 2018). Similar Pfeiffer effect was reported for *D*_3_-symmetrical labile TbIII(dpa)_3_ in the absence and presence of *L*-histidine, revealing no significant alteration in PL and CPL spectral profiles at 7F5-5D4 transition (Wu et al., 1989).

From Figure S19A in SM, we can see minimal alterations in magnitudes, wavelengths, and profiles in PL bands of Eu(fod)_3_ (non-chiral additive (red line), ***D*-Glu** (blue line), ***L*-Glu** (green line), and **CTA** (black line) at the five ^5^D_0_-^5^F_*J*_ (*J* = 0,1,2,3,4) transitions. Any apparent change in PL wavelength (593.0 nm) of Eu(fod)_3_ (non-chiral additive, ***D*-Glu**, ***L*-Glu**) is not detectable, while subtle changes in wavelength and magnitude in PL spectra of Eu(fod)_3_ (non-chiral (593.0 nm) and **CTA** (592.0 nm)) is discernable. Clearly, PL intensity at ^5^D_0_-^5^F_0_ transition (580 nm) of non-chiral additive Eu(fod)_3_ increases in the presence of chiral additive in the order of ***L*-Glu** < ***D*-Glu** < **CTA**. Similar alterations in subtle increases and subtle spectral shifts at ^5^D_0_-^5^F_*J*_ (*J* = 2,3,4) transitions between Eu(fod)_3_ (non-chiral additive, ***D*-Glu**, ***L*-Glu**, and **CTA**) is observable (Figures S19C–E), SM). We thus conclude that *C*_3_-symmetrical Eu(fod)_3_ in CDCl_3_ (0.8 x10^−2^ M) maintains in the presence of ***L*-Glu**, ***D*-Glu**, **CTA** (3–4 × 10^−2^ M, 4–5-folds excess relative to Eu(fod)_3_).

To confirm the dynamic equilibrium shift of the Δ-Λ isomers, we compare CPL and PL spectra of (a) Eu(fod)_3_ (0.8 × 10^−2^ M, 10 mg) and **CTA** (3 × 10^−2^ M, 20 mg) in 1.2 mL of CDCl_3_ (red line) and (b) thin solid films of Eu(fod)_3_ (10 mg, ~5 × 10^−1^ M) in **CTA** (20 mg) annealed at 100 °C in a vacuum overnight (blue line).

Previously, a simple thermodynamic analysis confirmed that the binding constant *K*_b_ using *g*_lum_ value at ^5^D_0_-^5^F_1_ transition between Eu(fod)_3_ and rigid chiral hydrocarbon, α-pinene, was on the order of 10^−3^ M^−1^; 0.7 × 10^−3^ M^−1^ for (1*S*)-α-pinene and 1.0 × 10^−3^ M^−1^ for (1*R*)-α-pinene (Jalilah et al., 2018). When a solvent quantity of α-pinene was used, even minimal *K*_b_ value was characterized as *g*_lum_ by CPL spectroscopy.

When a similar analysis was applied to Eu(fod)_3_ and non-rigid chiral **CTA**, we obtained *K*_b_ = 0.09 M^−1^ (Figures S21A,B), SM). This *K*_b_ value is more significant than that of α-pinene by two orders of magnitude and it is reasonable because a chiral repeating unit in **CTA** (though non-rigid and floppy) contains five oxygen atoms with the substantial (–)-Mulliken charges responsible for multiple pseudo chiral O/H–C interactions with achiral ligands (Figure 9 and Figure S18, SM). When solidified film and high concentrations of **CTA** were employed as chirality inducible scaffolds and platforms, the degree of chirogenesis was characterizable as *g*_lum_ values from CPL spectral characteristics. This idea can be extended to **CABu** and other four monosaccharide alkyl esters (Table 1).

## Conclusion

Two polysaccharide alkyl esters (**CTA** and **CABu**) as the films, two enantiopairs of monosaccharide permethyl esters (***D*-**/***L*-Glu** and ***D*-/*L*-Ara**) as the films, and (1*S*)-/(1*R*)-α-pinene in solution were capable of transferring their chirality to several optically inactive Eu^III^ and Tb^III^ tris(β-diketonate) (= fod and dpm), which impart the shining CPL characteristics at 4*f-*4*f* transitions. The greatest *g*_lum_ values at ^5^${\text{D}}_{0}\to^{7}$F_1_ transitions (λ_ex_ = 315 nm) of Eu(fod)_3_ in **CABu** and **CTA** films are +0.067 and +0.046, respectively. Tb(fod)_3_ in **CABu** and **CTA** exhibited moderately large *g*_lum_ values of +0.008 and +0.004 at ^5^${\text{D}}_{4}\to^{7}$F_5_ transitions (λ_ex_ = 315 nm), respectively. Meanwhile, ***D*-**/***L*-Glu** and ***D*-**/***L*-Ara** films induced weaker *g*_lum_ values for Eu(fod)_3_, Tb(fod)_3_, and Tb(dpm)_3_. **CTA** and **CABu** induced CPL signals more efficiently for Eu(fod)_3_ than ***D*-**/***L*-Glu** and ***D*-/*L*-Ara**. Noticeably, the chirality of α-pinene enabled Tb(dpm)_3_ to shine similar CPL characteristics of Tb(fod)_3_ in **CABu**. However, Eu(dpm)_3_ in **CABu** films and α-pinene did not reveal CPL. From the analyses of solution ^1^H-/^19^F-NMR, solid-state ^13^C-NMR with the help of MP2 (6–311G basis set) calculation, we propose that the surplus charge neutralization evaluated by the opposite Mulliken charges between *H*(δ+)-C(δ–) bonds of the poly- and monosaccharides and *F*(δ–)-C(δ+) bonds of the fluorinated ligands are the attractive driving forces to induce the CPL characteristics of Tb(fod)_3_ and Eu(fod)_3_. The present knowledge should enable the fabrication of films, sheets, fibers, and nanocomposites that emit Eu^III^-origin red-color and Tb^III^-origin green-color CPL spectra with narrow spectral bandwidths. As demonstrated, Eu^III^(fod)_3_ and Tb^III^(dpm)_3_ containing transparent **CTA** films deposited on the Tempax substrate displayed clear Eu^III^-origin red-color and Tb^III^-origin green-color emissions upon 365-nm excitation (see, photographs in Figure S22, SM). These materials were obtainable by a chiral ligand-free process at room temperature by co-mixing soluble polysaccharide derivatives (and bacterial cellulose) and several optically inactive Eu^III^/Tb^III^ complexes. The challenging issue remains to boost the rather small *g*_lum_ values [Eu(fod)_3_: 6 × 10^−2^ at 593 nm and Tb(fod)_3_: 0.8 × 10^−2^ at 540 nm] toward an ultimate *g*_lum_ = ±2.0, i.e., obtaining purely left- or right-CPL forms (Eliel and Wilen, 1994). Symmetry-oriented designing of emitters should be considered by precisely controlling topological shape associated with an efficient lens and an optofluidic effect (Wang et al., 2007; Di Pietro and Di Bari, 2012; Kruk et al., 2014; Khorasaninejad et al., 2016; Yeung et al., 2017; Tanaka et al., 2018; Zhou et al., 2019). A deeper understanding of the Pfeiffer effect in the GS and ES, magnetic dipole transitions of Eu^III^ and Tb^III^ complexes, colloidal aggregations, hybridization by other chromophores/luminophores, chain-like polymers, supramolecular motifs and polymers, and nature of oligo- and polysaccharides with conformational freedom are the next challenges (Wormald et al., 2002; Zou et al., 2019) in addition to several approaches to elaborate CPL and CD functions as polymeric colloids, revealing moderately high |*g*_lum_| and |*g*_abs_| values (>10^−2^-10^−1^) in the range of 300 and 800 nm (Nakano and Fujiki, 2011; Duong and Fujiki, 2017; Fujiki and Yoshimoto, 2017; Wang et al., 2017).

However, our approaches of chirogenesis from optically inactive labile Ln^III^ tris(β-diketonate) (Ln: lanthanide) induced by soluble chiral biomaterials is very limited to common organic solvents of Ln^III^ complexes and biomaterials. If water-soluble optically inactive labile Ln^III^ complexes are designed in the future, our approaches are applicable as a thin film state to sense and detect various water-soluble chiral substances including biomaterials, drug, medicine, pesticide, and virus consisting of illness-causing single-strand (*ss*)/double-strand (*ds*) RNA.

## Data Availability Statement

The raw data supporting the conclusions of this article will be made available by the authors, without undue reservation. Requests for the original CPL/CPLE/CD/UV-visible/NMR/IR spectral and photodynamic data sets, followed by the processed data (#.qpc with #.qda and #.txt) using KaleidaGraph (mac, ver 4.53), and the calculation results (#.com, #.log, and #.chk up to 20 GB) of Gaussian09 (mac) to support the conclusion of this article should be sent to MF ( fujikim@ms.naist.jp).

## Author Contributions

All the authors co-designed this work. MF, LW, and AJ co-wrote the paper. LW, NO, AJ, and MF co-measured and co-analyzed the CPL, CPLE, CD, UV-visible, PL, and PLE spectra of Tb(fod)_3_, Eu(fod)_3_, Tb(dpm)_3_, and Eu(dpm)_3_ and other several lanthanide complexes in the presence of chiral additives and chiral solvents. LW and FA co-acquired and co-analyzed ss-^13^C{^1^H}-FT-NMR and solution ^1^H-NMR/^19^F-NMR spectra. FA conducted the elemental analysis of the products. MF performed MP2 and DFT calculations. LW, NO, AJ, AO, SO, HK, and MF contributed to a joint project of emerging CPL spectra from achiral organic, polymeric, and lanthanide luminophores endowed with chiral polymers and chiral solvents. All authors discussed the data and commented on the manuscript. All authors have given approval to the final version of the manuscript. These authors contributed equally. The manuscript was written through contributions of all authors.

## Conflict of Interest

The authors declare that the research was conducted in the absence of any commercial or financial relationships that could be construed as a potential conflict of interest.

## Supplementary Material

The Supplementary Material for this article can be found online at: https://www.frontiersin.org/articles/10.3389/fchem.2020.00685/full#supplementary-material Characterization (^1^H-/^19^F-NMR, CP-MAS-^13^C-FT-NMR, FT-IR, HR-ESI-MS spectra) of **CTA**, **CABu**, ***D*-/*L*-Glu**, ***D*-/*L*-Ara**, Eu(fod)_3_, Tb(dpm)_3_, and Eu(dpm)_3_. Detailed measurement and analytical conditions of all instruments (solution ^1^H-/^19^F-FT-NMR, solid-state CP-MAS-^13^C-FT-NMR). CPL/PL spectra of Eu(dpm)_3_ in **CABu** and Eu(dpm)_3_ in (*S*)-/(*R*)-α-pinene. Mulliken charges of ***D*-Glu dimer** obtained with MP2 (6-311 G basis set) calculation. Photodynamic decay curves of Eu(dpm)_3_ in **CTA**, Eu(dpm)_3_ in **CABu**, Eu(fod)_3_ in **CTA**, Eu(fod)_3_ in **CABu**, Tb(dpm)_3_ in **CTA**, and Tb(dpm)_3_ in **CABu**, raw CD and UV-visible spectra of Eu(fod)_3_, Tb(fod)_3_, and Tb(fod)_3_ in **CTA** and **CABu** films and Eu(fod)_3_ in *D*-/*L*-**Glu** and *D*-/*L*-**Glu** films.
