# Corrigendum: Chirogenesis and Pfeiffer Effect in Optically Inactive Eu^III^ and Tb^III^ Tris(β-diketonate) Upon Intermolecular Chirality Transfer From Poly- and Monosaccharide Alkyl Esters and α-Pinene: Emerging Circularly Polarized Luminescence (CPL) and Circular Dichroism (CD)

**DOI:** 10.3389/fchem.2020.598729

**Published:** 2020-10-02

**Authors:** Michiya Fujiki, Laibing Wang, Nanami Ogata, Fumio Asanoma, Asuka Okubo, Shun Okazaki, Hiroki Kamite, Abd Jalil Jalilah

**Affiliations:** ^1^Division of Materials Science, Graduate School of Science and Technology, Nara Institute of Science and Technology, Ikoma, Japan; ^2^School of Materials Engineering, Universiti Malaysia Perlis, Jejawi, Malaysia; ^3^Centre of Excellence Frontier Materials Research, Universiti Malaysia Perlis, Kangar, Malaysia

**Keywords:** non-covalent interaction, circularly polarized luminescence, circular dichroism, europium, terbium, cellulose, saccharide, terpene

In the original article, there were mistakes in Table 1 as published. Table 1 aimed to compare all *g*_lum_ values at specific wavelengths of four Ln^III^ tris(β-diketonates) (Ln^III^: Eu^III^ and Tb^III^) upon intermolecular chirality transfer from **CABu**, **CTA**, **Glu** (*D*- and *L*-), **Ara** (*D*- and *L*-), and α-pinene for clarity and readability. However, although the *g*_lum_ values described in the main text are correct, most data in the three right-side columns of Table 1 are incorrectly displayed. Also, in Table 1 caption, *D*-/*L*-glucose pentameyhyl esters should be *D*-/*L*-glucose pentamethyl esters. The corrected Table 1 with corrected numerical values in the three right-side columns and corrected caption appears below.

**Table 1:** CPL characteristics (dissymmetry ratio, *g*_lum_ in 10^−2^ at specific wavelength) of Eu^III^ and Tb^III^ coordinated with three β-diketonates as achiral ligands embedded in two polysaccharide alkyl esters (**CABu** and **CTA**), *D*-/*L*-glucose pentamethyl esters (***D*-**/***L****-***Glu**), and *D*-/*L*-Arabinose tetramethyl esters (***D*-/*L-*Ara**).

**Ln^III^ tris(β- diketonates)**	**CABu *g*_lum_/10^−2^ (nm)**	**CTA *g*_lum_/10^−2^ (nm)**	**Glu *g*_lum_/10^−2^ (nm)**		**Ara *g*_lum_/10^−2^ (nm)**		***α-*pinene *g*_lum_/10^−2^ (nm)**	
			***D*-**	***L*-**	***D*-**	***L*-**	**(1*R*)**	**(1*S*)**
Eu(fod)_3_	+6.71 (593)^a^ −0.59 (613)^b^	+4.63 (593)^a^ −0.40 (613)^b^	+1.05 (594)^a^ −0.19 (612)^b^	−0.81 (596)^a^ +0.08 (613)^b^	+0.19 (593)^a^ −0.02 (607)^b^	−0.30 (591)^a^ +0.06 (611)^b^	−0.49 (593)^f^ +0.05 (613)^f^	+0.41 (593)^f^ −0.04 (613)^f^
Eu(dpm)_3_	n.d. ^g^	n.d. ^g^	n.d. ^g^	n.d. ^g^	n.d. ^g^	n.d. ^g^	n.d. ^g^	n.d. ^g^
Tb(fod)_3_	−0.29 (490)^c^ +0.78 (540)^d^ −0.18 (552)^e^	−0.10 (490)^c^ +0.35 (542)^d^ −0.07 (553)^e^	n.d. ^g^	n.d. ^g^	n.d. ^g^	n.d. ^g^	n.d. ^g^	n.d. ^g^
Tb(dpm)_3_	−0.53 (491)^c^ +0.37 (537)^d^ −0.59 (547)^e^	−0.44 (489)^c^ – +0.80 (547)^e^	n.d. ^g^	n.d. ^g^	n.d. ^g^	n.d. ^g^	n.d^g^ (~490) +0.44^d^ (537) −0.13^e^ (547)	n.d^g^ (~490) −0.49^d^ (537) +0.34^e^ (548)

*All numerical values in bracket mean wavelength extremum for CPL signals*.

a *Eu^III^^5^${\text{D}}_{0}\to^{7}$F_1_ (593 nm)*,

b *Eu^III5^${\text{D}}_{0}\to^{7}$F_2_ (613 nm)*,

c *Tb^III^^5^${\text{D}}_{4}\to^{7}$F_6_ (490 nm)*,

d *Tb^III5^${\text{D}}_{4}\to^{7}$F_5_ (I) (540 nm)*,

e *Tb^III5^${\text{D}}_{4}\to^{7}$F_5_ (II) (552 nm)*,

f *Data were taken from literature (Jalilah et al., 2018)*.

g *Not characterized or no data*.

Also, there was an error in Introduction. The sentence starting with “Particularly, chirogenesis in metal coordination chemistry by the chirality transfer has long been one of the central subjects in inorganic chemistry 1 (Mason and Norman, 1965…)” should read as follows: “Particularly, chirogenesis in metal coordination chemistry by the chirality transfer has long been one of the central subjects in inorganic chemistry (Mason and Norman, 1965…)”.

The authors apologize for these errors and state that this does not change the scientific conclusions of the article in any way. The original article has been updated.
