# Supplementary material for: Chirogenesis and Pfeiffer Effect in Optically Inactive EuIII and TbIII Tris(β-diketonate) Upon Intermolecular Chirality Transfer From Poly- and Monosaccharide Alkyl Esters and α-Pinene: Emerging Circularly Polarized Luminescence (CPL) and Circular Dichroism (CD)
Source: Front Chem. 2020 Aug 11;8:685. doi: 10.3389/fchem.2020.00685 (PMC7438854; doi:10.3389/fchem.2020.00685)
Supplement: Supplementary file 1 [file Data_Sheet_1.pdf]

Supplementary Materials for

Chirogenesis and Pfeiffer Effect in Optically Inactive Eu^{III} and Tb^{III} Tris(*β*-diketonate) upon Intermolecular Chirality Transfer from Poly- and Monosaccharide Alkyl Esters and *α*-Pinene: Emerging Circularly Polarized Luminescence (CPL) and Circular Dichroism (CD)

Michiya Fujiki[1,*], Laibing Wang[1], Nanami Ogata[1], Fumio Asanoma[1], Asuka Okubo[1], Shun Okazaki[1], Hiroki Kamite[1], Abd Jalil Jalilah[1,2,3*]

[1] Division of Materials Science, Graduate School of Science and Technology, Nara Institute of Science and Technology, 8916-5 Takayama, Ikoma, Nara, 630-0192, Japan.
[2] School of Materials Engineering, Universiti Malaysia Perlis, Kompleks Pusat Pengajian Jejawi 2, Taman Muhibah, Jejawi, 02600 Arau, Perlis, Malaysia
[3] Centre of Excellence Frontier Materials Research, Universiti Malaysia Perlis (UniMAP), Jalan Kangar-Alor Setar, Kampung Seriab, 01000 Kangar, Perlis, Malaysia

Experimental Section

Materials. The esterification of glucose (TCI, Tokyo, Japan) and arabinose (TCI, Tokyo, Japan) were conducted in line with a previous report.[1] Tb(fod)$_3$ was prepared by modifying a previous report.[2] (1*S*)- and (1*R*)-α-Pinene (TCI) were purified by distillation under reduced pressure.[3]

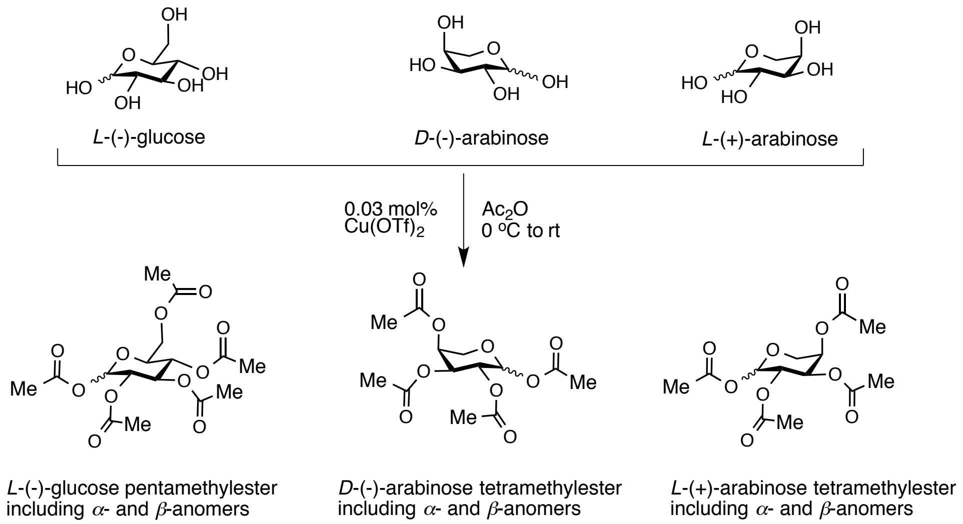

Scheme S1. Synthetic route of ***L*-(-)-Glu**, ***D*-(-)-Ara**, and ***L*-(+)-Ara** by acetylation.

References:

(S1) Tai, C. A., Kulkarni, S. S., Hung, S. C. (2003). Facile Cu(OTf)$_2$-catalyzed preparation of per-*O*-acetylated hexopyranoses with stoichiometric acetic anhydride and sequential one-pot anomeric substitution to thioglycosides under solvent-free conditions. *J. Org. Chem*. 68, 8719-8722. doi:org/10.1021/jo030073b

(S2) Katagiri, S., Hasegawa, Y., Wada, Y., Mitsuo, K., and Yanagida, S. (2006). Temperature-dependent energy transfer in photo-sensitized luminescence of rare earth complexes. *J. Alloy. Compd*. 408, 809-812. doi:org/10.1016/j.jallcom.2005.01.134

(S3) Jalilah, A. J., Asanoma, F., and Fujiki, M. (2018). Unveiling controlled breaking of the mirror symmetry of Eu(fod)$_3$ with α-/β-pinene and BINAP by circularly polarised luminescence (CPL), CPL excitation, and ^{19}F-/^{31}P{1H}-NMR spectra and Mulliken charges. *Inorg. Chem. Front*. 5, 2718–2733. doi:org/10.1039/C8QI00509E

• Conditions of CP-MAS-solid-state ^{13}C-NMR measurement.

• ***D*-Glu-Eu(fod)$_3$**

```
レポートが作成されました:  6-AUG-2018 10:16:52
----------------------------------

Filename               = Glucose-EuFOD_cpmas_13C-1-2.jdf
Author                 = delta
Experiment             = cpmas_toss.jxp
Sample_Id              = S#506671
Solvent                = NONE
Creation_Time          = 26-DEC-2017 14:04:58
Revision_Time          =  6-AUG-2018 10:15:50
Current_Time           =  6-AUG-2018 10:16:52

Comment                = Cross Polarization with TOSS, cogwheel
Data_Format            = 1D COMPLEX
Dim_Size               = 8192
Dim_Title              = Carbon13
Dim_Units              = [ppm]
Dimensions             = X
Site                   = JNM-ECX400
Spectrometer           = DELTA2_NMR

Field_Strength         = 9.38977[T] (400[MHz])
X_Acq_Duration         = 50.91328[ms]
X_Domain               = 13C
X_Freq                 = 100.5253[MHz]
X_Offset               = 100[ppm]
X_Points               = 2048
X_Prescans             = 0
X_Resolution           = 19.64124[Hz]
X_Sweep                = 40.22526[kHz]
X_Sweep_Clipped        = 40.22526[kHz]
Irr_Domain             = Proton
Irr_Freq               = 399.7822[MHz]
Irr_Offset             = 5[ppm]
Clipped                = FALSE
Scans                  = 1100
Total_Scans            = 1100

Relaxation_Delay       = 5[s]
Recvr_Gain             = 68
Temp_Get               = 460.0[dC]
Contact_Time           = 2[ms]
X_Acq_Time             = 50.91328[ms]
X_Dwell                = 24.86[us]
X_Pulse                = 0.1[us]
Dec_Setup              = #Setup Decoupling#
```

• **CTA-Eu(fod)$_3$**

```
レポートが作成されました: 18-SEP-2018 08:33:34
----------------------------------

Filename               = CTA-EuFOD_cpmas-1-2.jdf
Author                 = delta
Experiment             = cpmas_toss.jxp
Sample_Id              = S#652487
Solvent                = NONE
Creation_Time          =  9-AUG-2017 18:08:36
Revision_Time          = 18-SEP-2018 08:33:08
Current_Time           = 18-SEP-2018 08:33:34

Comment                = Cross Polarization with TOSS, cogwheel
Data_Format            = 1D COMPLEX
Dim_Size               = 8192
Dim_Title              = Carbon13
Dim_Units              = [ppm]
Dimensions             = X
Site                   = JNM-ECX400
Spectrometer           = DELTA2_NMR

Field_Strength         = 9.38977[T] (400[MHz])
X_Acq_Duration         = 50.91328[ms]
X_Domain               = 13C
X_Freq                 = 100.5253[MHz]
X_Offset               = 100[ppm]
X_Points               = 2048
X_Prescans             = 0
X_Resolution           = 19.64124[Hz]
X_Sweep                = 40.22526[kHz]
X_Sweep_Clipped        = 40.22526[kHz]
Irr_Domain             = Proton
Irr_Freq               = 399.7822[MHz]
Irr_Offset             = 5[ppm]
Clipped                = FALSE
Scans                  = 1100
Total_Scans            = 1100

Relaxation_Delay       = 5[s]
Recvr_Gain             = 70
Temp_Get               = 460.0[dC]
Contact_Time           = 2[ms]
X_Acq_Time             = 50.91328[ms]
X_Dwell                = 24.86[us]
X_Pulse                = 0.1[us]
Dec_Setup              = #Setup Decoupling#

Irr_Amp_Cp             = 70[%]
Irr_Amp_Dec            = 100[%]
Irr_Amp_Prep           = 100[%]
Irr_Atn                = 0[dB]
Irr_Noise              = TPPM
Irr_Phs_Tppm           = 15[deg]
Irr_Pwidth             = 2.78[us]
Irr_Setup              = #Setup Irradiation#
Irr_Shape_Cp           = constant_cp
Irr_Width_90           = 2.78[us]
Irr_Width_Nominal90    = 2.78[us]
Obs_Amp_Cp             = 63[%]
Obs_Amp_Grad           = 9[%]
Obs_Amp_Toss           = 100[%]
Obs_Atn                = 3.3[dB]
Obs_Shape_Cp           = RAMP_cp
Obs_Width_Toss         = 5.6[us]
A                      = 0.81118
Acq                    = 4
Atn_Setup              = #Experiment Attenuator Settings#
Autoshim_Track         = AUTOSHIM OFF
B                      = 1.76995
C                      = 2.18882
Cp_Setup               = #Setup CP#
D                      = 3.23005
Initial_Wait           = 10[ms]
Mas_Freq               = 8[kHz]
Minimum_Interval       = of timing 10:   C - B = 0.418874
Minimum_Interval_In_Us = 52.35875[us]
Note                   = Recommended for fast spinning: ACQ = 4*Tr
Prep_Pulse             = TRUE
Recycle_Setup          = #Setup Recycle Times#
Repetition_Time        = 5.05091[s]
Req_Scans              = 1100
Toss                   = TRUE
Toss_Interval          = 98.5975[us]
Toss_Interva2          = 0.11425[ms]
Toss_Interva3          = 46.75875[us]
Toss_Interva4          = 0.12455[ms]
Toss_Interva5          = 93.44375[us]
Toss_Timing            = 10
Ttoss                  = # Setup TOSS parameters#
```

- Conditions of solution ^{1}H-NMR measurement.

  - **Hfod**

```
レポートが作成されました:  6-AUG-2018 10:22:11
----------------------------------

Filename         = HFOD-1H-3.jdf
Author           = delta
Experiment       = single_pulse.exp
Sample_Id        = 1
Solvent          = CHLOROFORM-D
Creation_Time    = 26-DEC-2017 14:12:57
Revision_Time    =  6-AUG-2018 10:21:57
Current_Time     =  6-AUG-2018 10:22:11

Comment          = Single Pulse Experiment
Data_Format      = 1D COMPLEX
Dim_Size         = 16384
Dim_Title        = 1H
Dim_Units        = [ppm]
Dimensions       = X
Site             = ECP400
Spectrometer     = DELTA_NMR

Field_Strength   = 9.38977[T] (400[MHz])
X_Acq_Duration   = 2.73121[s]
X_Domain         = 1H
X_Freq           = 399.7822[MHz]
X_Offset         = 5[ppm]
X_Points         = 16384
X_Prescans       = 0
X_Resolution     = 0.36614[Hz]
X_Sweep          = 5.9988[kHz]
Clipped          = FALSE
Scans            = 8
Total_Scans      = 8

Relaxation_Delay = 4[s]
Recvr_Gain       = 23
Temp_Get         = 19.9[dC]
X_90_Width       = 14[us]
X_Acq_Time       = 2.73121[s]
X_Angle          = 45[deg]
X_Pulse          = 7[us]
Initial_Wait     = 1[s]
Phase_Preset     = 3[us]
Unblank_Time     = 2[us]
```

- Conditions for the measurement of ^{19}F-NMR solution.

  - **Eu(fod)$_3$**

```
レポートが作成されました: 18-SEP-2018 08:39:20
----------------------------------

Filename         = 19F_EuFOD_CDCl3_180315-2.jdf
Author           = delta
Experiment       = single_pulse.exp
Sample_Id        = 1
Solvent          = CHLOROFORM-D
Creation_Time    = 15-MAR-2018 10:28:41
Revision_Time    = 18-SEP-2018 08:38:47
Current_Time     = 18-SEP-2018 08:39:20

Comment          = Single Pulse Experiment
Data_Format      = 1D COMPLEX
Dim_Size         = 32768
Dim_Title        = 19F
Dim_Units        = [ppm]
Dimensions       = X
Site             = ECP400
Spectrometer     = DELTA_NMR

Field_Strength   = 9.38977[T] (400[MHz])
X_Acq_Duration   = 0.43254[s]
X_Domain         = 19F
X_Freq           = 376.17105[MHz]
X_Offset         = -100[ppm]
X_Points         = 32768
X_Prescans       = 0
X_Resolution     = 2.31194[Hz]
X_Sweep          = 75.75758[kHz]
Clipped          = FALSE
Scans            = 64
Total_Scans      = 64

Relaxation_Delay = 4[s]
Recvr_Gain       = 17
Temp_Get         = 20.6[dC]
X_90_Width       = 14[us]
X_Acq_Time       = 0.43254[s]
X_Angle          = 45[deg]
X_Pulse          = 7[us]
Initial_Wait     = 1[s]
Phase_Preset     = 3[us]
Unblank_Time     = 2[us]
```

  - **Tb(fod)$_3$**

```
レポートが作成されました: 18-SEP-2018 08:38:12
----------------------------------

Filename         = Tb_fod3_19F-1-2.jdf
Author           = delta
Experiment       = proton.jxp
Sample_Id        = S#506329
Solvent          = CHLOROFORM-D
Creation_Time    = 27-AUG-2018 14:04:06
Revision_Time    = 18-SEP-2018 08:37:56
Current_Time     = 18-SEP-2018 08:38:11

Comment          = single_pulse
Data_Format      = 1D COMPLEX
Dim_Size         = 26214
Dim_Title        = Fluorine19
Dim_Units        = [ppm]
Dimensions       = X
Site             = JNM-ECA600
Spectrometer     = DELTA2_NMR

Field_Strength   = 14.09637[T] (600[MHz])
X_Acq_Duration   = 0.23069[s]
X_Domain         = 19F
X_Freq           = 564.72612[MHz]
X_Offset         = -100[ppm]
X_Points         = 32768
X_Prescans       = 1
X_Resolution     = 4.33488[Hz]
X_Sweep          = 142.04545[kHz]
X_Sweep_Clipped  = 113.63636[kHz]
Irr_Domain       = Fluorine19
Irr_Freq         = 564.72612[MHz]
Irr_Offset       = 5.0[ppm]
Tri_Domain       = Fluorine19
Tri_Freq         = 564.72612[MHz]
Tri_Offset       = 5.0[ppm]
Clipped          = FALSE
Scans            = 1267
Total_Scans      = 1267

Relaxation_Delay = 5[s]
Recvr_Gain       = 50
Temp_Get         = 18.8[dC]
X_90_Width       = 16.25[us]
X_Acq_Time       = 0.23069[s]

X_Angle          = 45[deg]
X_Atn            = 5[dB]
X_Pulse          = 8.125[us]
Irr_Mode         = Off
Tri_Mode         = Off
Dante_Presat     = FALSE
Initial_Wait     = 1[s]
Repetition_Time  = 5.23069[s]
```

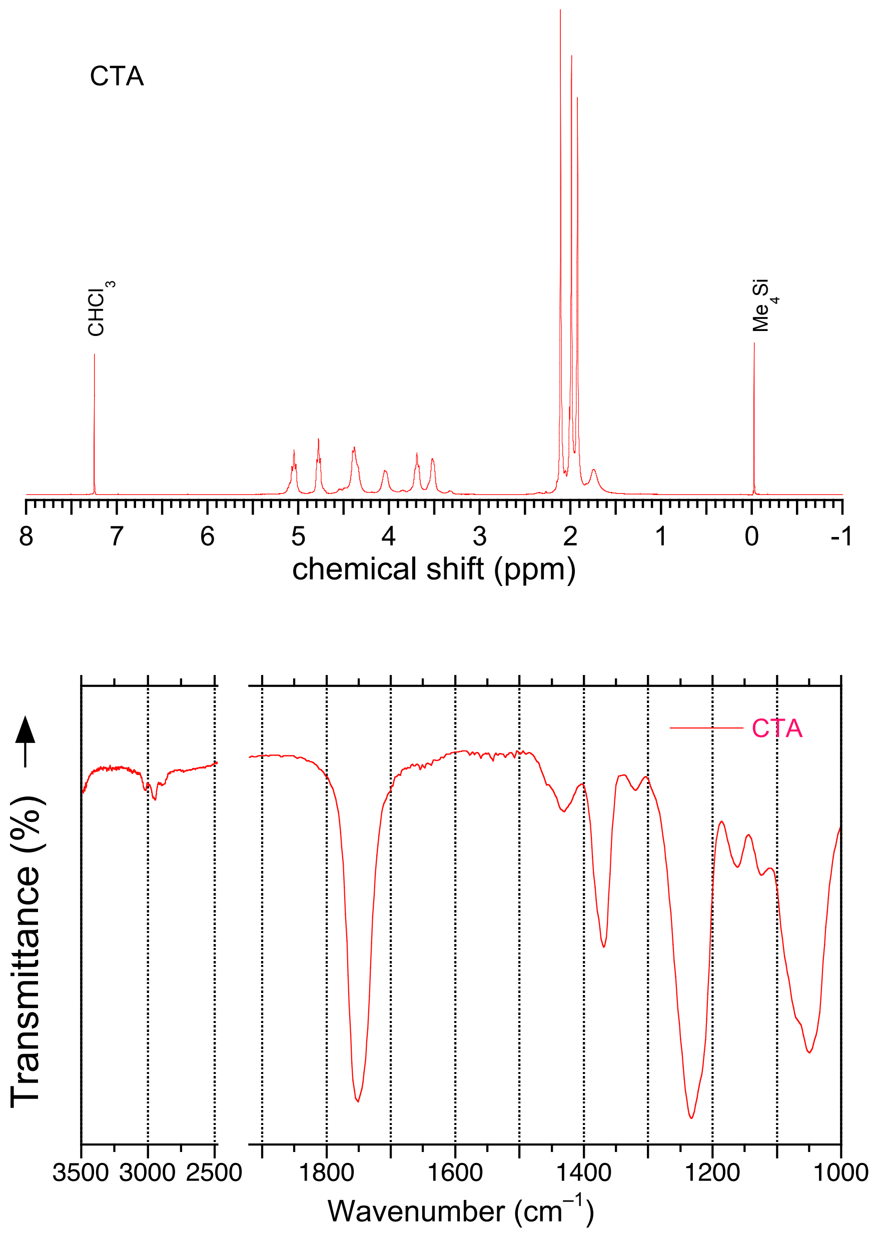

Figure S1. (Top) ^{1}H-NMR (in $CDCl_3$) and (bottom) FT-IR spectra (onto CaF_2) of **CTA** (Wako pure chemicals). ν(ester C=O):1749 cm^{-1}.

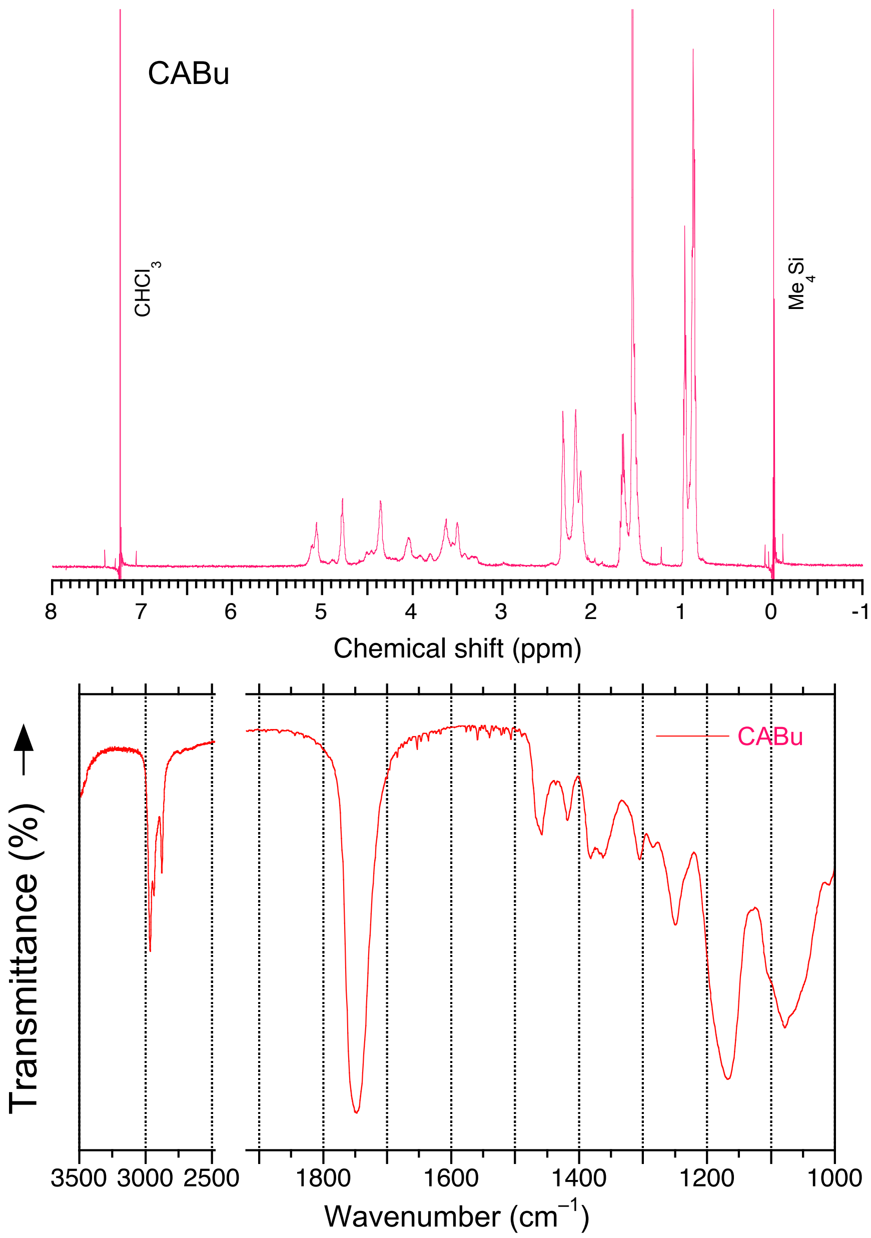

Figure S2. (Top) ^{1}H-NMR (in $CDCl_3$) and (bottom) FT-IR spectra (onto CaF_2) of **CABu** (Sigma-Aldrich). Any free OH groups at ~3300 cm^{-1} are not seen, suggesting that OH groups of non-substituted cellulose are fully replaced by acetyl and butyryl groups. ν(ester C=O):1746 cm^{-1}.

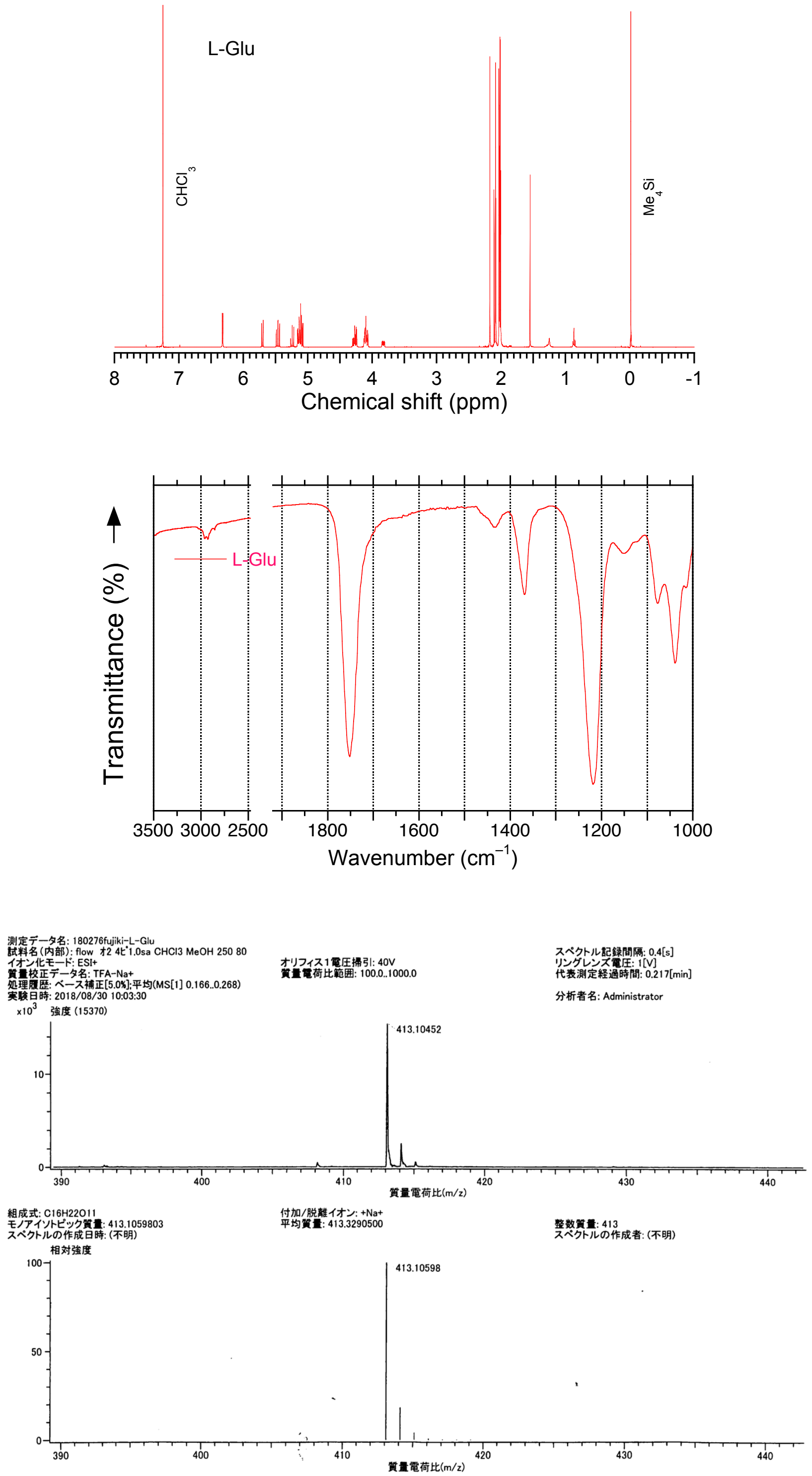

Figure S3. (Top) ^{1}H-NMR ($CDCl_3$), (middle) FT-IR spectra (CaF_2), and (bottom) ESI (positive mode)-MS spectra of ***L*-Glu**. ν(ester C=O):1753 cm^{-1}, *m*/*z* calculated for $C_{46}H_{22}O_{11}$with Na^+([M and Na]$^+$), 413.10598; found, 413.10452.

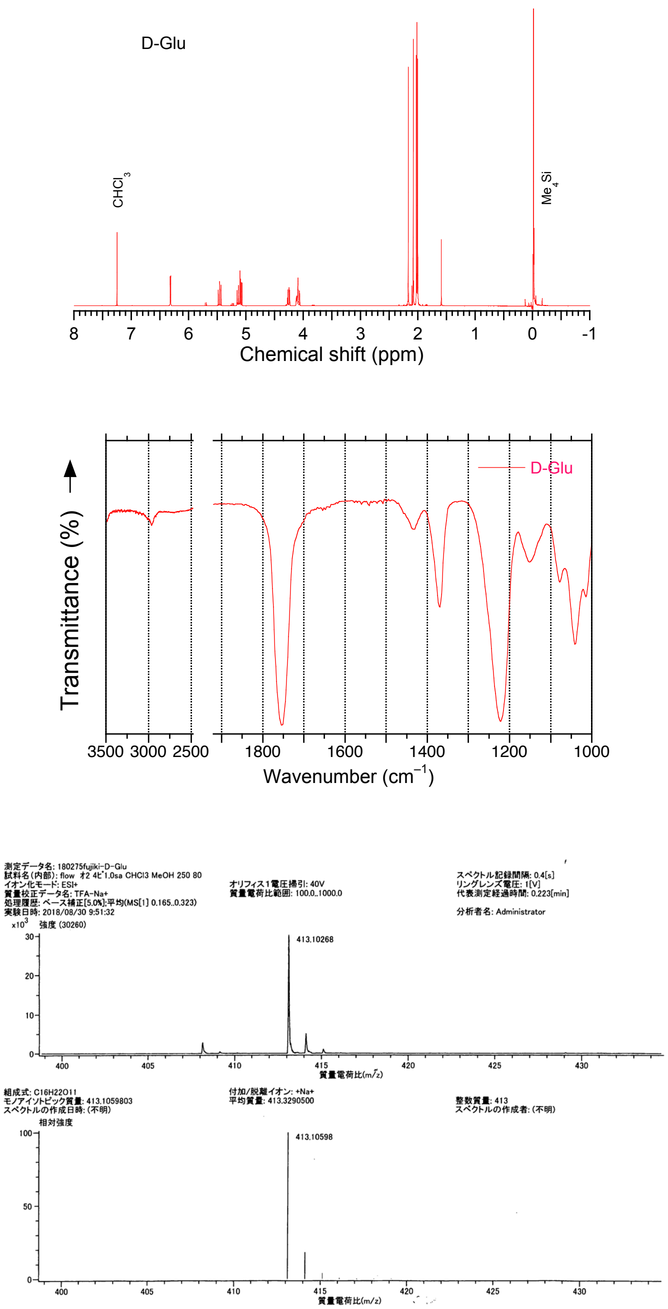

Figure S4. (Top) ^{1}H-NMR ($CDCl_3$), (middle) FT-IR spectra (CaF_2), and (bottom) ESI (positive mode)-MS spectra ***D*-Glu**. ν(ester C=O):1753 cm^{-1}. *m*/*z* calculated for $C_{46}H_{22}O_{11}$with Na^{+} ([M and Na]$^{+}$), 413.10598; found, 413.10268.

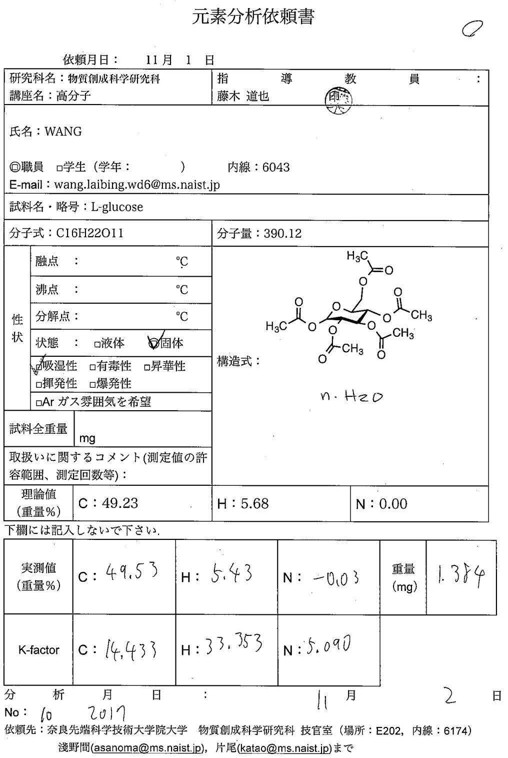

元素分析依頼書

依頼月日： 11月 1日

研究科名：物質創成科学研究科<br>講座名：高分子	指導教員：<br>藤木 道也
氏名：WANG<br>◎職員 □学生（学年： ） 内線：6043<br>E-mail：wang.laibing.wd6@ms.naist.jp	
試料名・略号：L-glucose	
分子式：C16H22O11	分子量：390.12
性状：融点 ： ℃<br>沸点 ： ℃<br>分解点： ℃<br>状態 ： □液体 ✓固体<br>✓吸湿性 □有毒性 □昇華性<br>□揮発性 □爆発性<br>□Ar ガス雰囲気を希望	構造式：<br>n·H2O
試料全重量 mg	
取扱いに関するコメント(測定値の許容範囲、測定回数等)：	

理論値（重量%）	C：49.23	H：5.68	N：0.00

下欄には記入しないで下さい。

実測値（重量%）	C：49.53	H：5.43	N：-0.03	重量 (mg)	1.384
K-factor	C：14.433	H：33.353	N：5.090		

分析月日： 11月 2日

No： 10 2017

依頼先：奈良先端科学技術大学院大学 物質創成科学研究科 技官室（場所：E202, 内線：6174）
淺野間(asanoma@ms.naist.jp), 片尾(katao@ms.naist.jp)まで

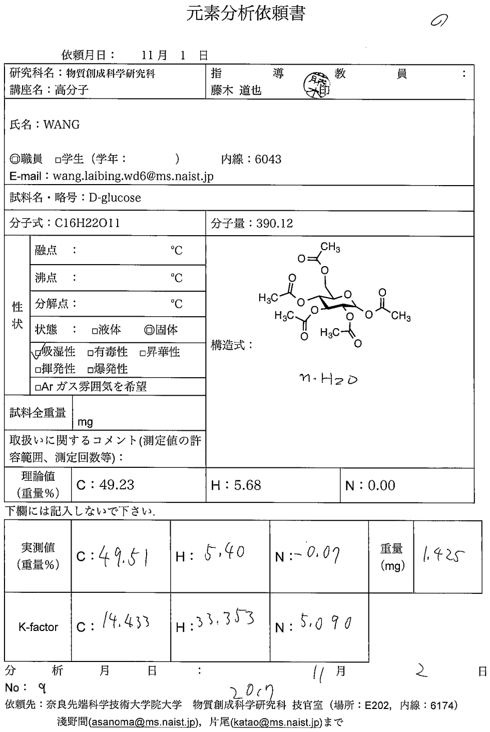

元素分析依頼書

依頼月日： 11月 1日

研究科名：物質創成科学研究科<br>講座名：高分子	指導教員：<br>藤木 道也
氏名：WANG<br>◎職員 □学生（学年： ） 内線：6043<br>E-mail：wang.laibing.wd6@ms.naist.jp	
試料名・略号：D-glucose	
分子式：C16H22O11	分子量：390.12
性状：融点 ： ℃<br>沸点 ： ℃<br>分解点： ℃<br>状態 ： □液体 ◎固体<br>✓吸湿性 □有毒性 □昇華性<br>□揮発性 □爆発性<br>□Ar ガス雰囲気を希望	構造式：<br>n·H2O
試料全重量 mg	
取扱いに関するコメント(測定値の許容範囲、測定回数等)：	

理論値（重量%）	C：49.23	H：5.68	N：0.00

下欄には記入しないで下さい。

実測値（重量%）	C：49.51	H：5.40	N：-0.07	重量 (mg)	1.425
K-factor	C：14.433	H：33.353	N：5.090		

分析月日： 11月 2日

No： 9 2017

依頼先：奈良先端科学技術大学院大学 物質創成科学研究科 技官室（場所：E202, 内線：6174）
淺野間(asanoma@ms.naist.jp), 片尾(katao@ms.naist.jp)まで

Figure S5. Elemental analysis of (left) ***L*-Glu** and (right) ***D*-Glu**.

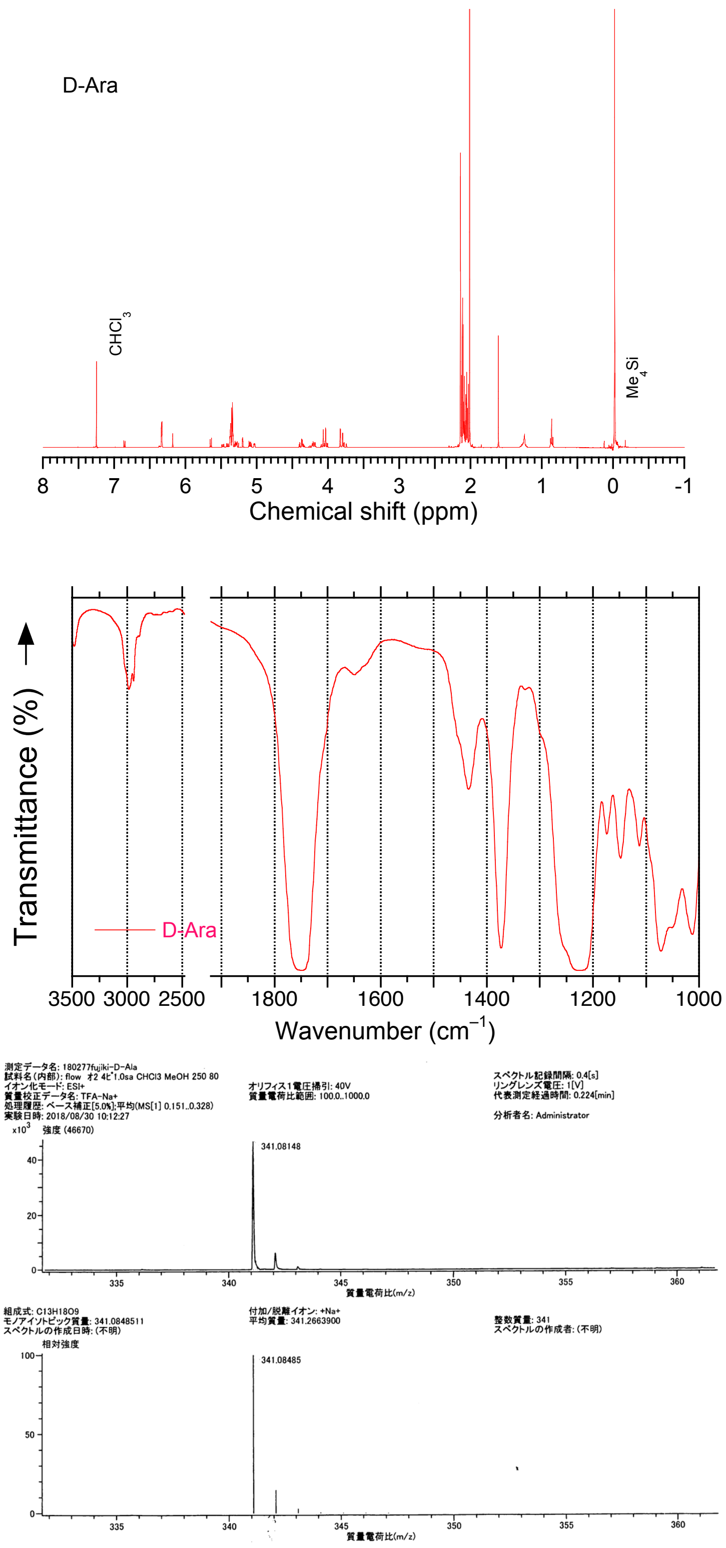

Figure S6. ^{1}H-NMR (in $CDCl_3$) and FT-IR spectra (onto CaF_2) of ***D*-Ara**. ν(ester C=O):1746 cm^{-1}, ESI-MS (positive): *m*/*z* calculated for $C_{13}H_{18}O_9$ with Na^+ ([M and Na]$^+$), 341.08485; found, 341.08148.

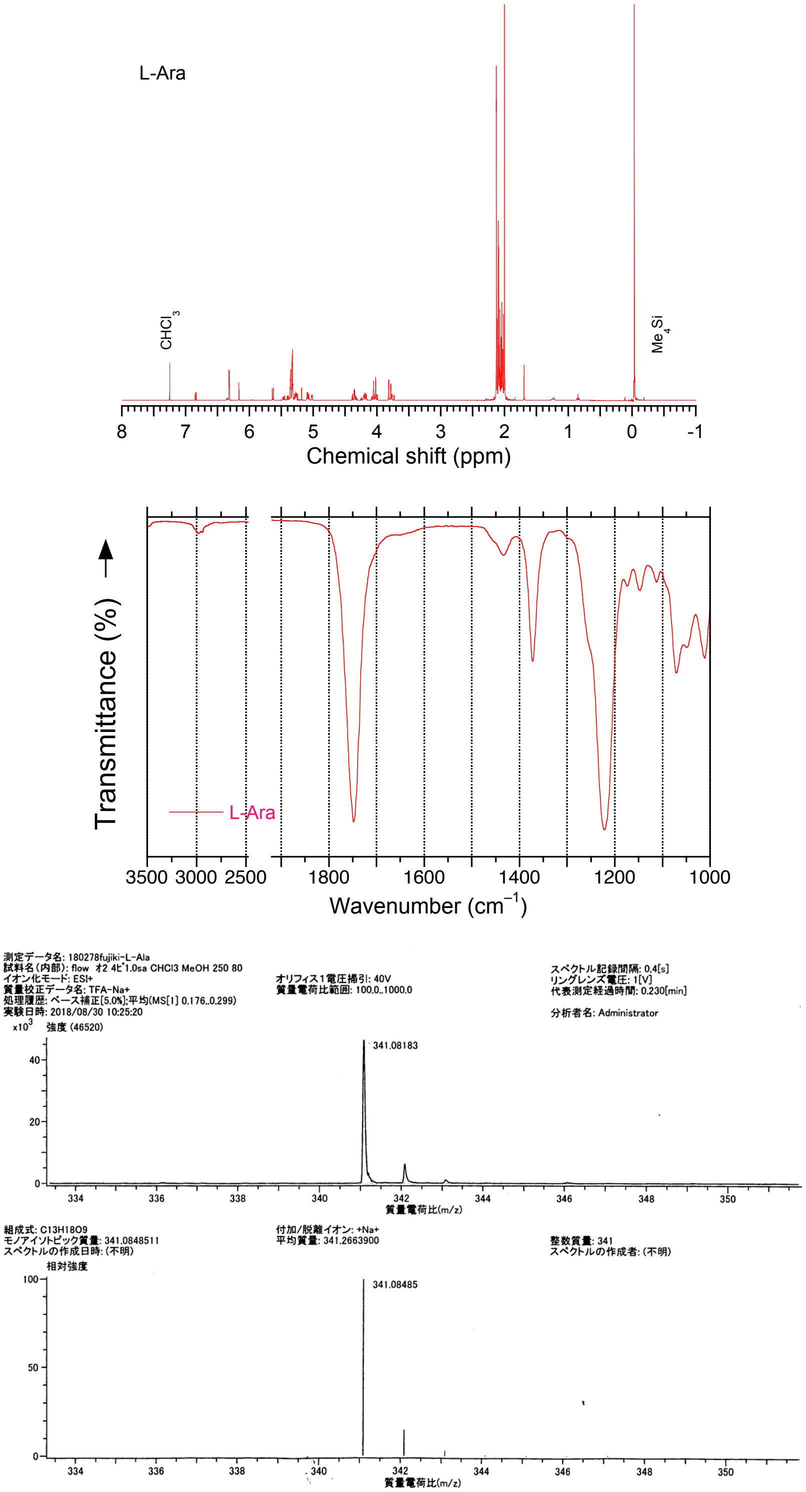

Figure S7. ^{1}H-NMR (in $CDCl_3$) and FT-IR spectra (onto CaF_2) of ***L*-Ara**. ν(ester C=O):1746 cm^{-1}. ESI-MS (positive): *m*/*z* calculated for $C_{13}H_{18}O_9$ with Na^+ ([M and Na]$^+$), 341.08485; found, 341.08183.

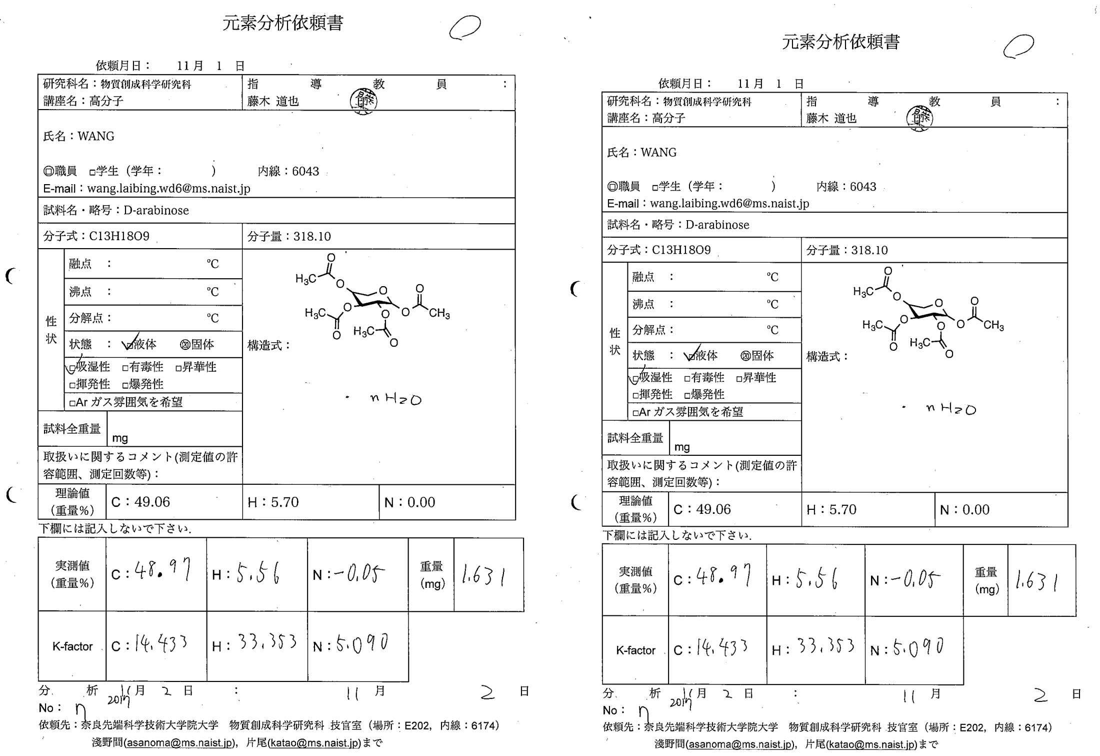

元素分析依頼書

依頼月日： 11月 1 日

研究科名：物質創成科学研究科<br>講座名：高分子	指 導 教 員 ：<br>藤木 道也
氏名：WANG<br>◎職員 □学生（学年： ） 内線：6043<br>E-mail：wang.laibing.wd6@ms.naist.jp	
試料名・略号：D-arabinose	
分子式：C13H18O9	分子量：318.10
性状　融点 ： ℃<br>沸点 ： ℃<br>分解点： ℃<br>状態 ： ✓液体 ◎固体<br>✓吸湿性 □有毒性 □昇華性<br>□揮発性 □爆発性<br>□Ar ガス雰囲気を希望	構造式： · nH2O
試料全重量 mg	
取扱いに関するコメント(測定値の許容範囲、測定回数等)：	

理論値（重量%）	C：49.06	H：5.70	N：0.00

下欄には記入しないで下さい.

実測値（重量%）	C：48.97	H：5.56	N：-0.05	重量 (mg)	1.631
K-factor	C：14.433	H：33.353	N：5.090		

分 析 2017 11月 2 日 ： 11 月 2 日

No：

依頼先：奈良先端科学技術大学院大学 物質創成科学研究科 技官室（場所：E202, 内線：6174）

浅野間(asanoma@ms.naist.jp), 片尾(katao@ms.naist.jp)まで

元素分析依頼書

依頼月日： 11月 1 日

研究科名：物質創成科学研究科 指 導 教 員 ： 講座名：高分子 藤木 道也

氏名：WANG

◎職員 □学生（学年： ） 内線：6043

E-mail：wang.laibing.wd6@ms.naist.jp

試料名・略号：D-arabinose

分子式：C13H18O9 分子量：318.10

理論値（重量%） C：49.06 H：5.70 N：0.00

下欄には記入しないで下さい.

実測値（重量%） C：48.97 H：5.56 N：-0.05 重量 (mg) 1.631

K-factor C：14.433 H：33.353 N：5.090

依頼先：奈良先端科学技術大学院大学 物質創成科学研究科 技官室（場所：E202, 内線：6174）

浅野間(asanoma@ms.naist.jp), 片尾(katao@ms.naist.jp)まで

Figure S8. Elemental analysis of (left) ***L*-Ara** and (right) ***D*-Ara**.

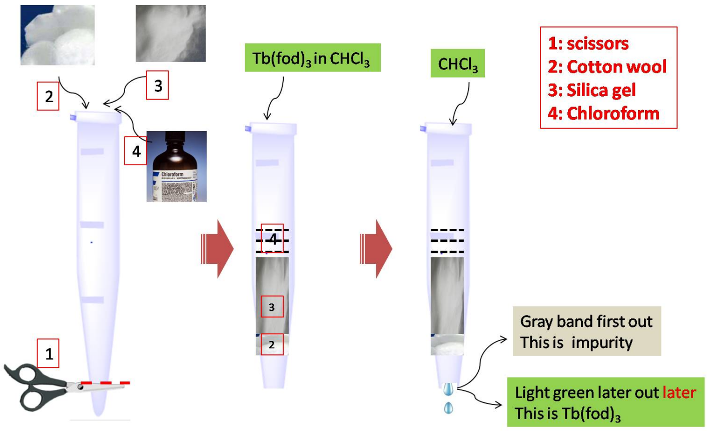

Chart S1. The purification method for $Tb(fod)_3$.

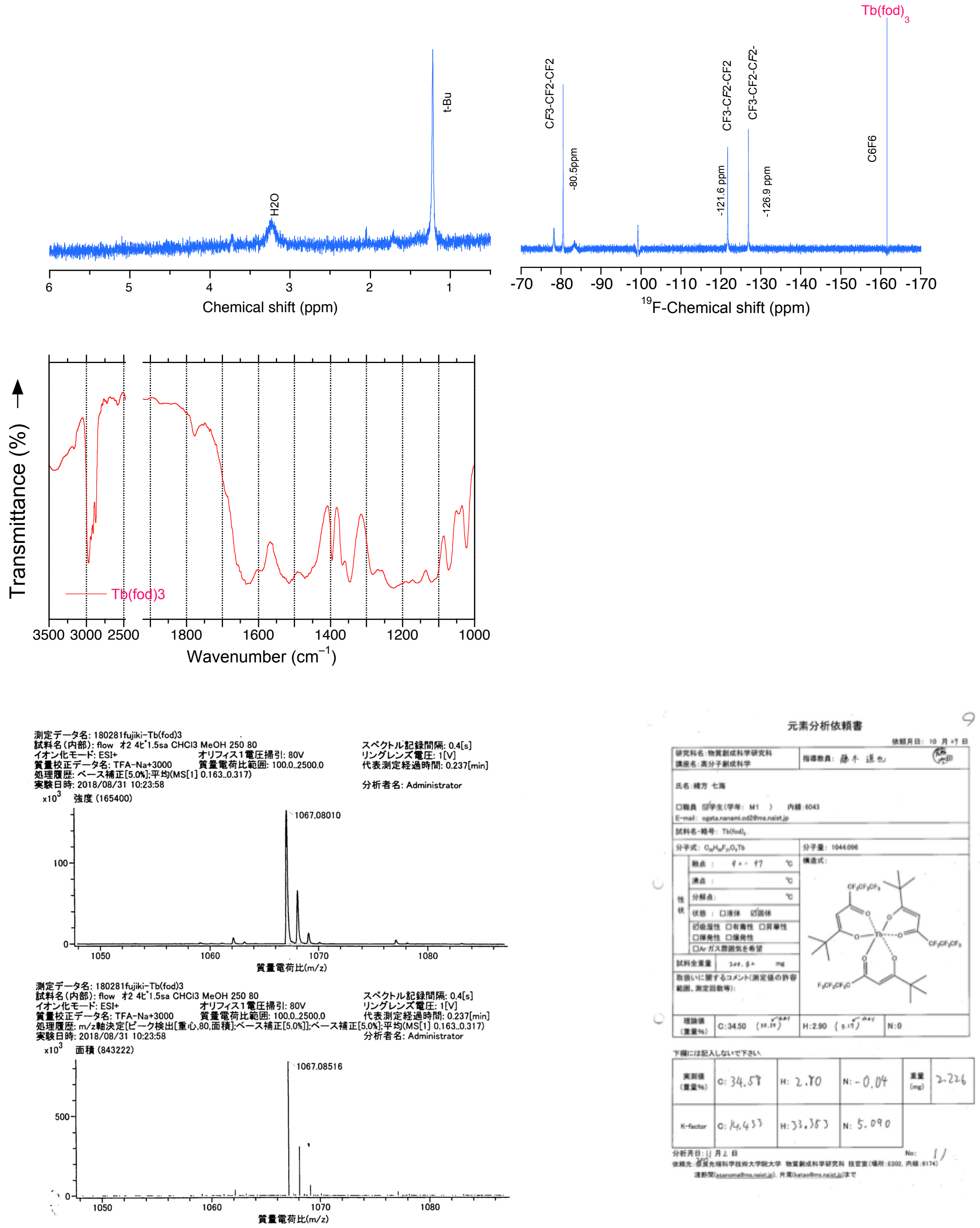

Figure S9. (Top, left) ^{1}H-NMR ($CDCl_3$, ref $(CH_3)_4Si$), (top, right) ^{19}F-NMR ($CDCl_3$, ref C_6F_6), (medium) FT-IR spectra (CaF_2) and (bottom, left) ESI-MS (positive mode) spectra, and (bottom, right) elemental analysis of Tb(fod)$_3$. ν(C=O, β-diketonate):1630 cm^{-1}.*m/z* calculated for $C_{30}H_{30}F_{21}TbO_6$ with Na^+ ([M and Na]$^+$), 1067.08516; found, 1067.08010.

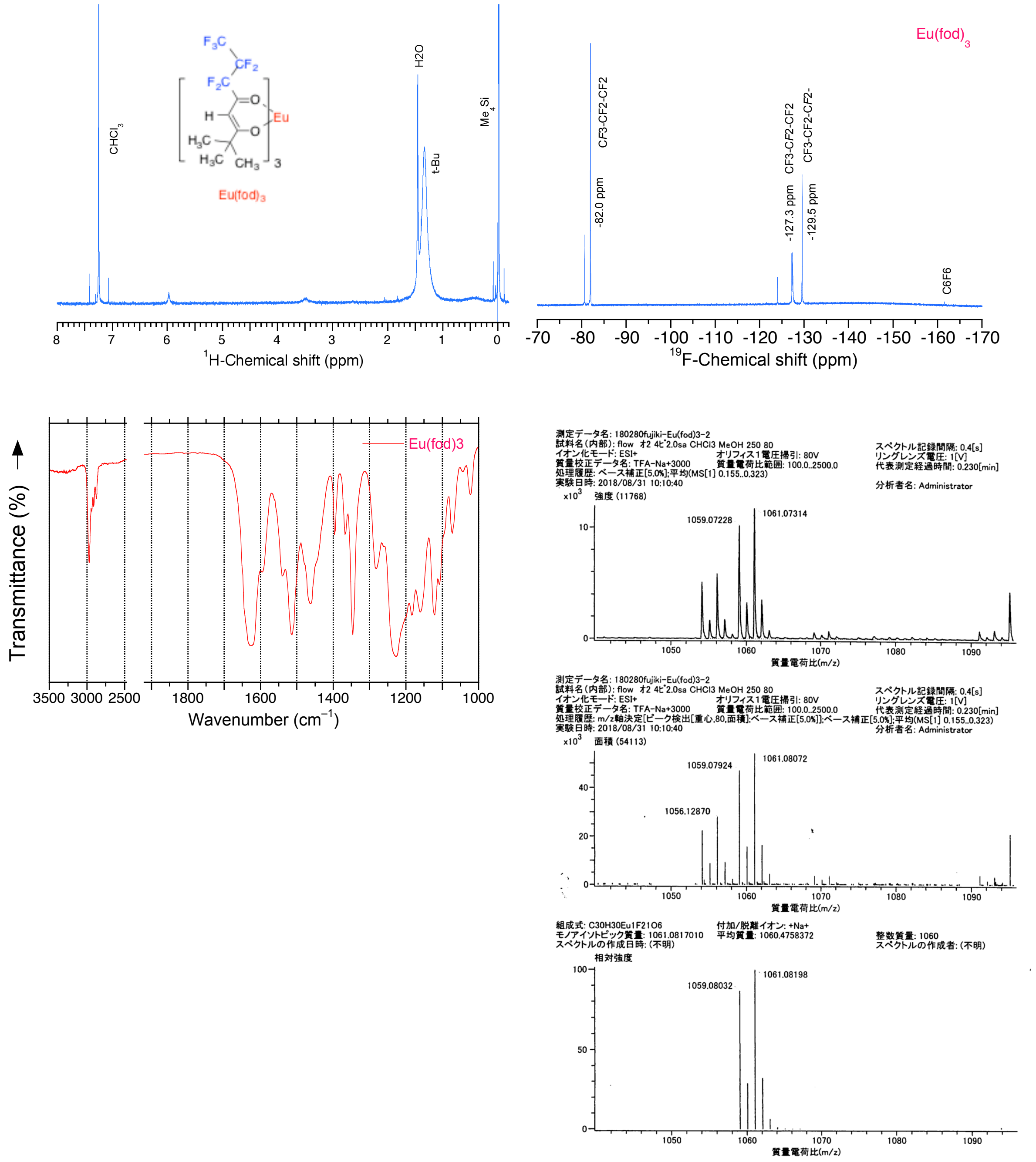

Figure S10. (Top, left) ^{1}H-NMR ($CDCl_3$, ref $(CH_3)_4Si$), (top, right) ^{19}F-NMR ($CDCl_3$, ref C_6F_6), (bottom, left) FT-IR (CaF_2) and (bottom, right) ESI-MS (positive mode) spectra of Eu(fod)$_3$ (Sigma-Aldrich). ν(C=O, β-diketonate):1620 cm^{-1}.*m*/*z* calculated for $C_{30}H_{30}EuF_{21}O_6$with Na^+ ([M and Na]$^+$), 1059.08032; found, 1059.07924.

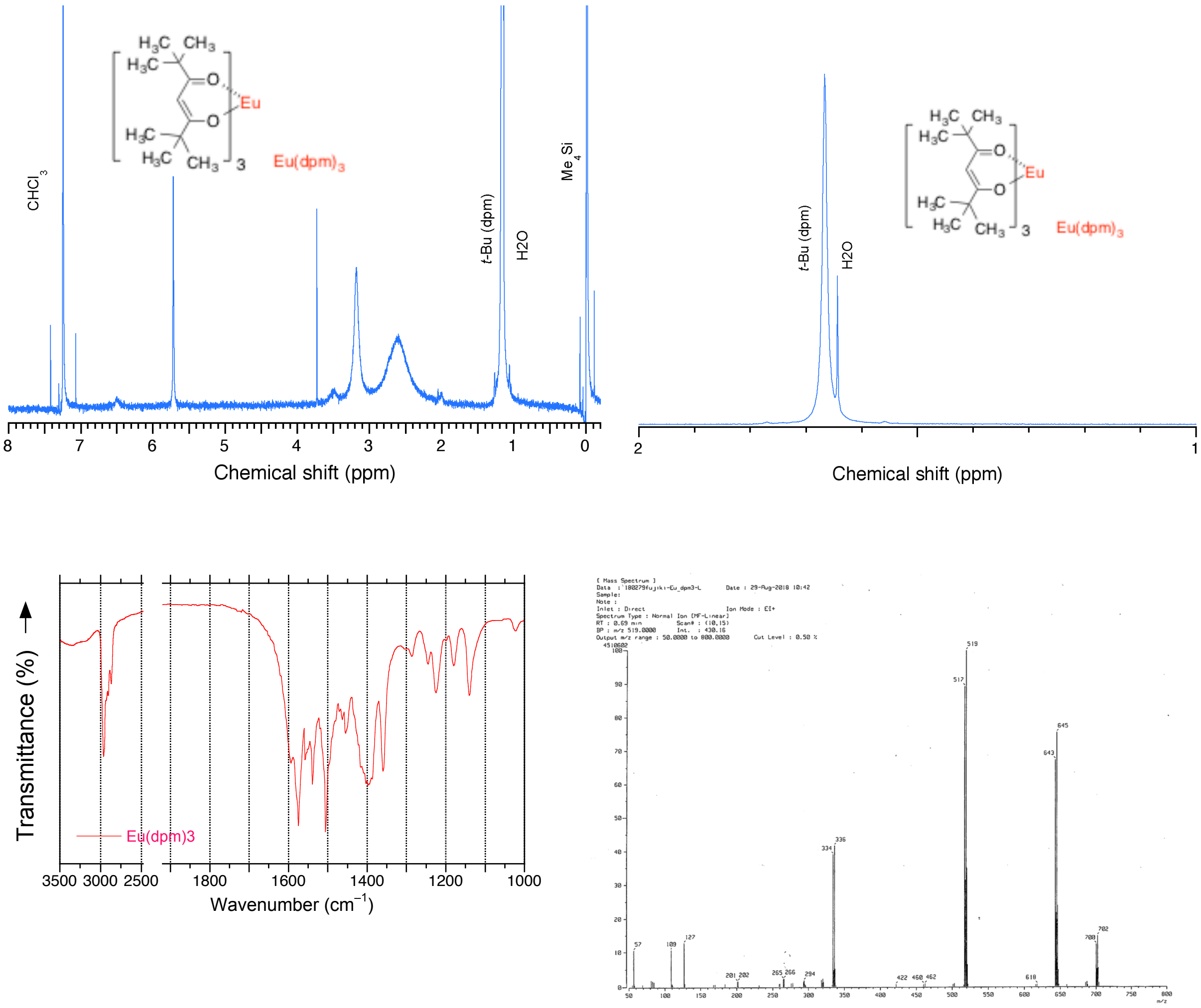

Figure S11. (Top, left) ^{1}H-NMR ($CDCl_3$, ref $(CH_3)_4Si$), (top, right) ^{19}F-NMR ($CDCl_3$, ref C_6F_6), (bottom, left) FT-IR (CaF_2) and (bottom, right) HR-EI-MS (positive mode) spectra of Eu(dpm)$_3$ (TCI). ν(C=O, β-diketonate):1574 cm^{-1}. *m*/*z* calculated for $C_{29}H_{49}EuO_6$ ($[M]^+$),700.3354; found, 700.3359.

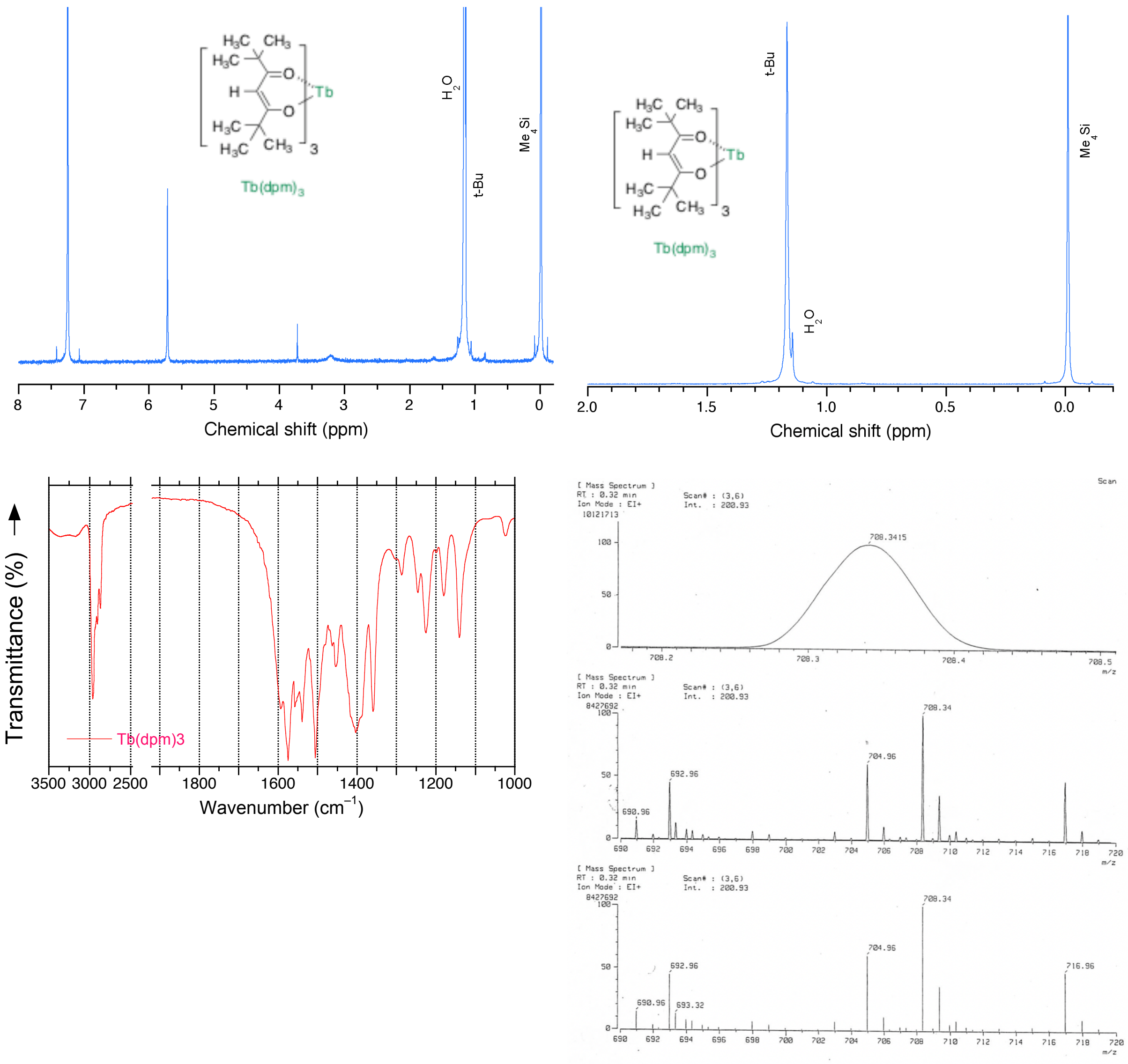

Figure S12. (Top, left) ^{1}H-NMR ($CDCl_3$, ref $(CH_3)_4Si$), (top, right) ^{19}F-NMR ($CDCl_3$, ref C_6F_6), (bottom, left) FT-IR (CaF_2) and (bottom, right) HR-EI-MS (positive mode) spectra of Tb(dpm)$_3$ (Sigma-Aldrich). ν (C=O, β-diketonate):1574 cm^{-1}.*m*/*z* calculated for $C_{33}H_{57}TbO_6$ ([M^+]), 708.3409; found, 708.3415.

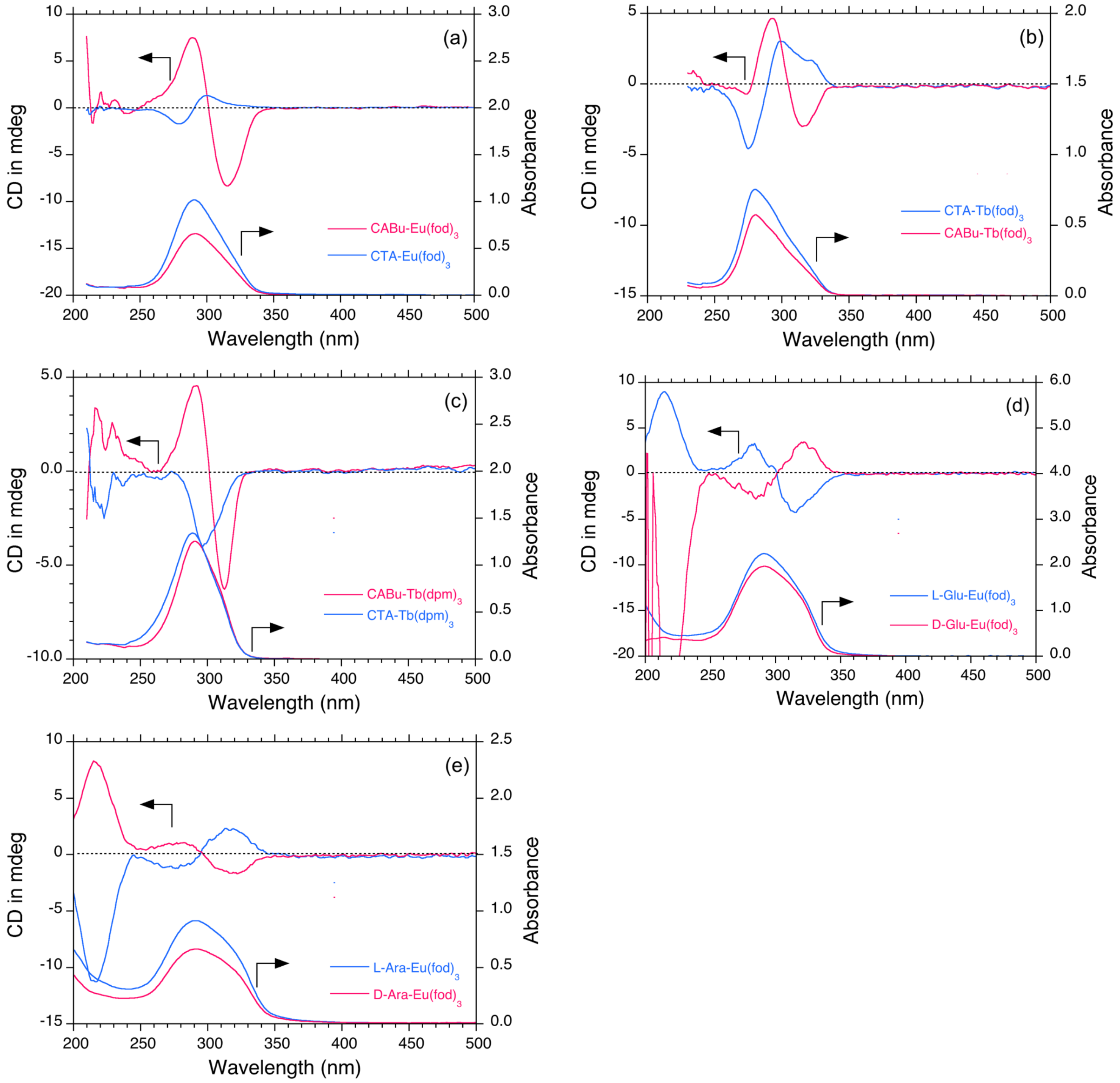

Figure S13. Raw CD and UV-visible spectra of (a) $Eu(fod)_3$ in **CTA** and **CABu** films, (b) $Tb(fod)_3$ in **CTA** and **CABu** films (c) $Tb(dpm)_3$ in **CTA** and **CABu** films, (d) $Eu(fod)_3$ in D-/L-**Glu** films, and (e) $Eu(fod)_3$ in D-/L-**Ara** films.

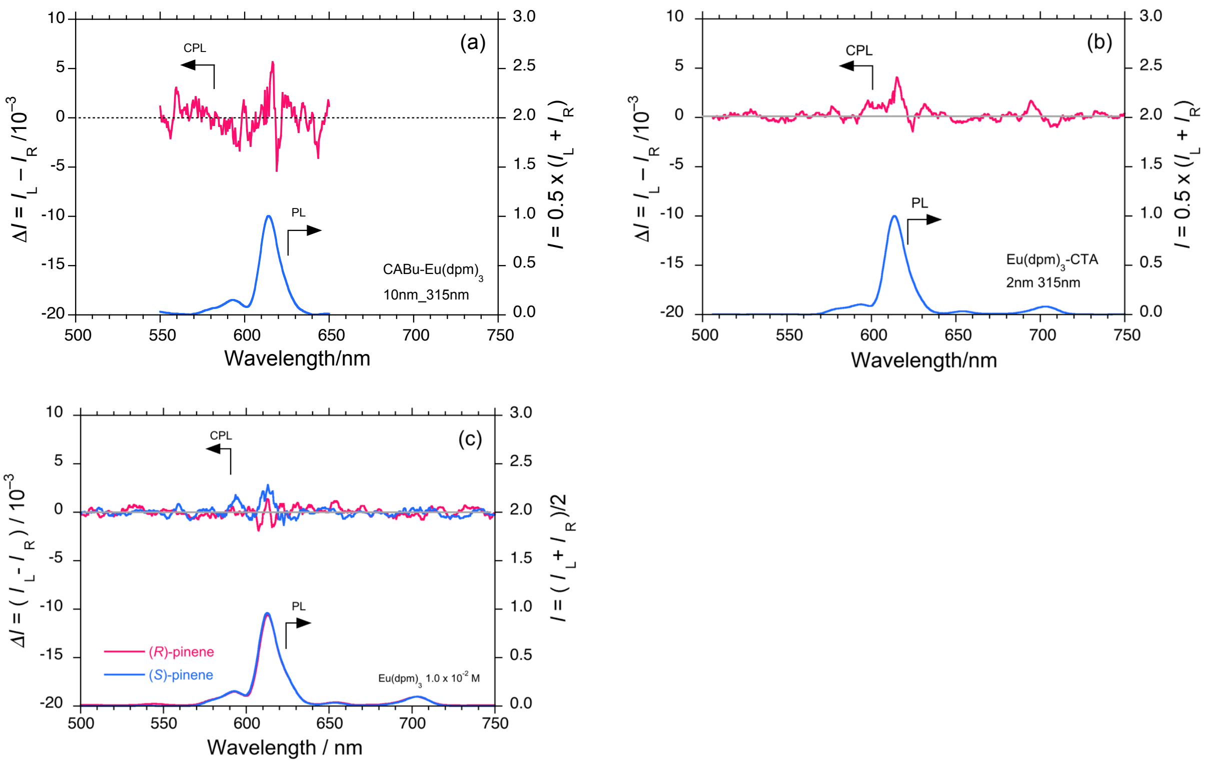

Figure S14. CPL and PL spectra of $Eu(dpm)_3$ in (a) **CABu** film and (b) **CTA** film excited at 315 nm. (c) CPL and PL spectra of $Eu(dpm)_3$ dissolved in (*R*)- and (*S*)-*α*-pinene excited at 320 nm. $Eu(dpm)_3$ does not reveal clear CPL signals associated with very weak PL signals. A red emission was faint by naked eyes.

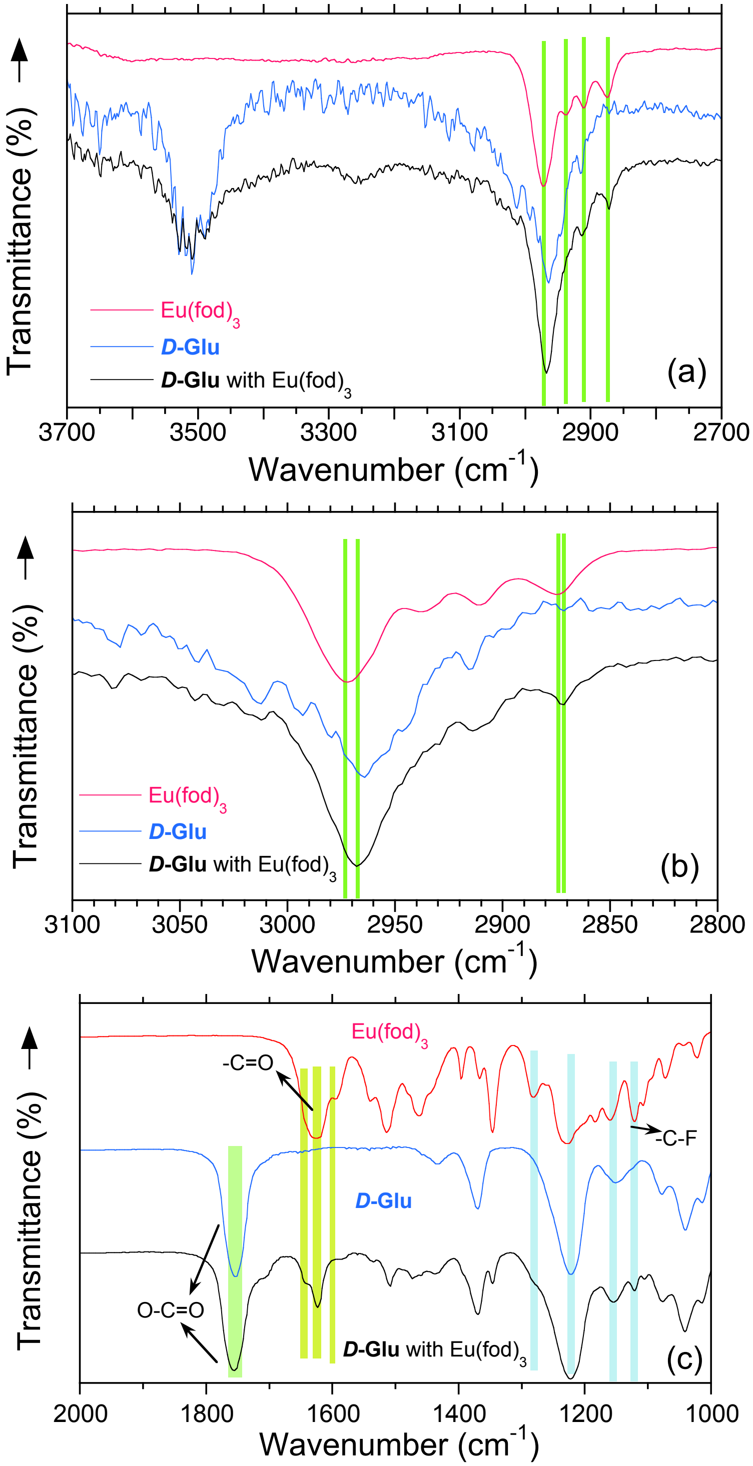

Figure S15. (a) Comparisons of FT-IR spectra between $Eu(fod)_3$, ***D*-Glu**, and $Eu(fod)_3$ mixed with ***D*-Glu** in the range of 2500 and 4000 cm^{-1}. (b) Their magnified FT-IR spectra in the range of 1000 and 2000 cm^{-1}.There is no clear evidence of ester group coordination of Eu(III) to $Eu(fod)_3$, and small frequency shifts due to the postulated (fod) C-*H*/*O*-C (glucose) interactions may be seen.

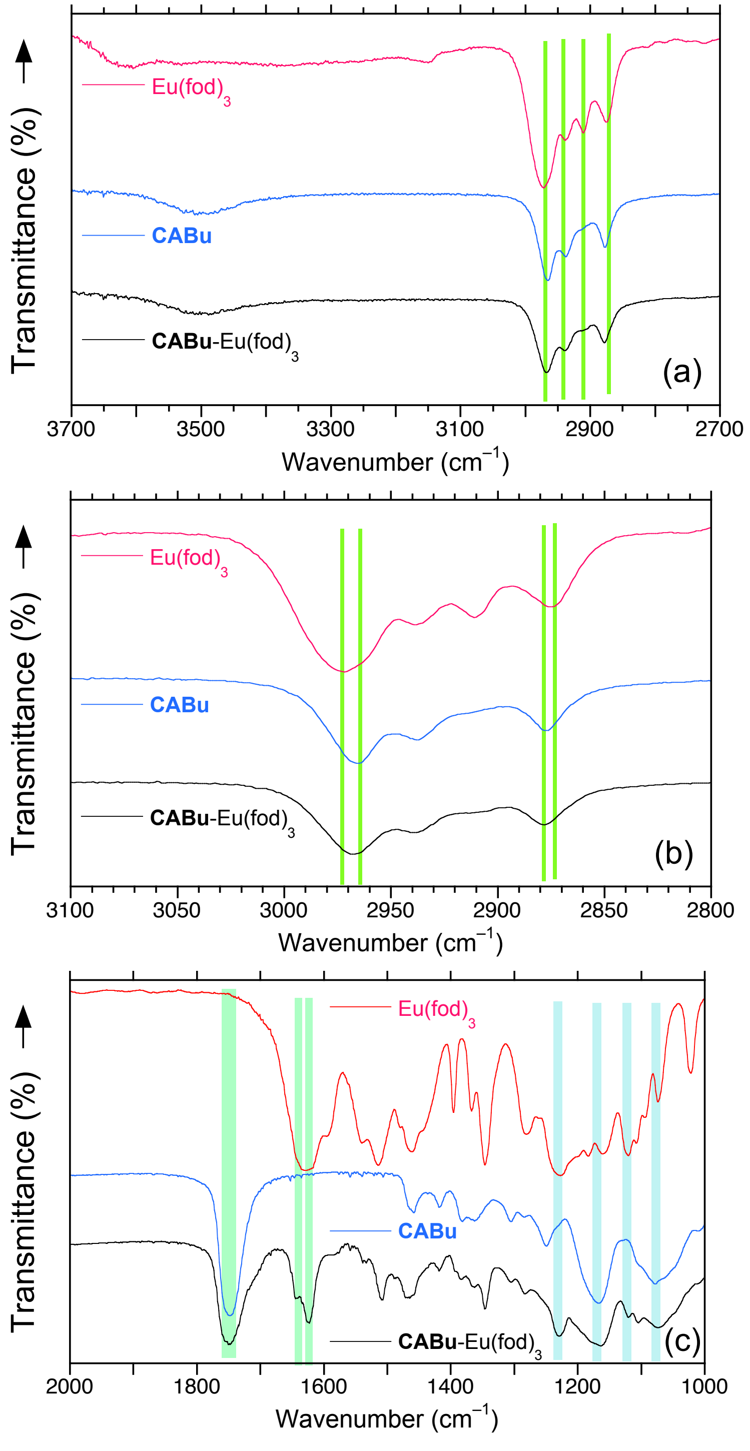

Figure S16. (a) Comparisons of FT-IR spectra between Eu(fod)$_3$, **CABu**, and Eu(fod)$_3$ mixed with **CABu** in the range of 2500 and 4000 cm^{-1}. (b) Their magnified FT-IR spectra are in the range of 1000 and 2000 cm^{-1}.There is no clear evidence of ester group coordination to Eu(fod)$_3$, and small frequency shifts due to the postulated (fod) C-*H*/*O*-C (**CABu**) interactions can be seen.[4]

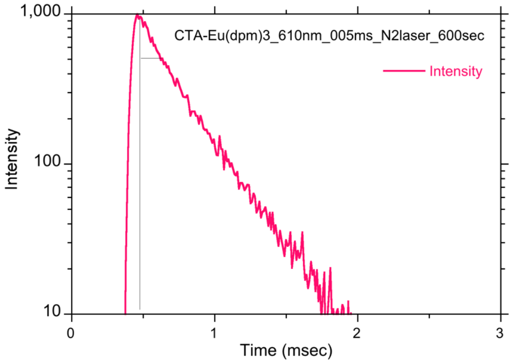

(a) Eu(dpm)$_3$ in **CTA@610nm**

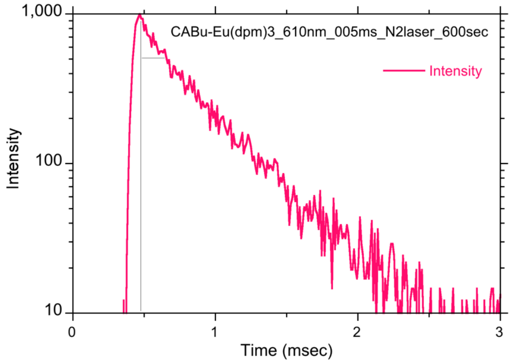

(b) Eu(dpm)$_3$ in **CABu@610nm**

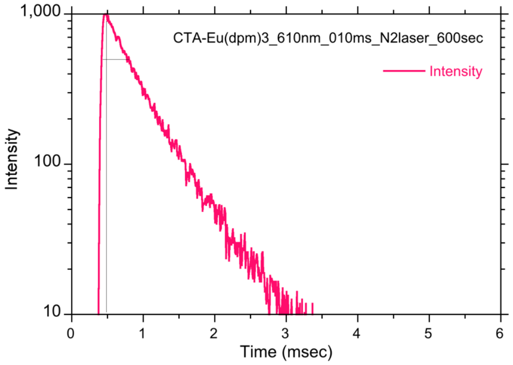

(c) Eu(fod)$_3$ in **CTA@610nm**

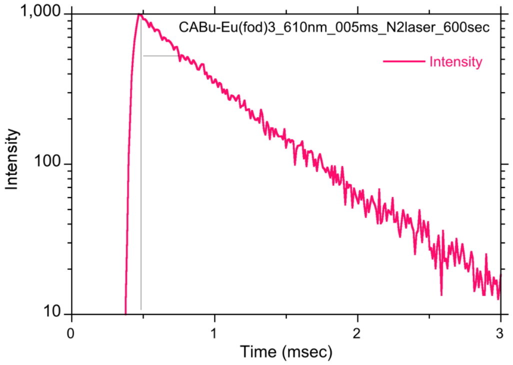

(d) Eu(fod)$_3$ in **CABu@610nm**

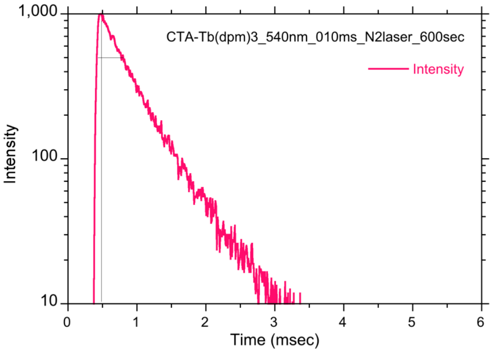

(e) Tb(dpm)$_3$ in **CTA@540nm**

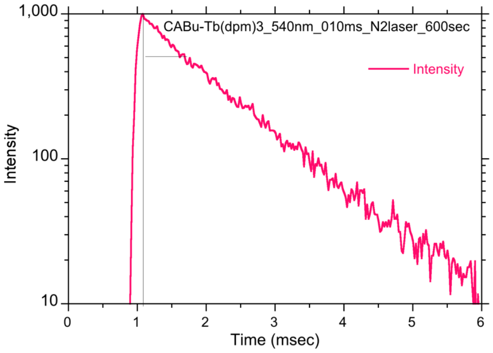

(f) Tb(dpm)$_3$ in **CABu@540nm**

Figure S17. Photodynamic decay curves in semilog plots of (a) Eu(dpm)$_3$ in **CTA** at 610nm, (b) Eu(dpm)$_3$ in **CABu** at 610nm, (c) Eu(fod)$_3$ in **CTA** at 610nm, (d) Eu(fod)$_3$ in **CABu** at610nm, (e) Tb(dpm)$_3$ in **CTA** at 540nm, and (f) Tb(dpm)$_3$ in **CABu** at 540nm.

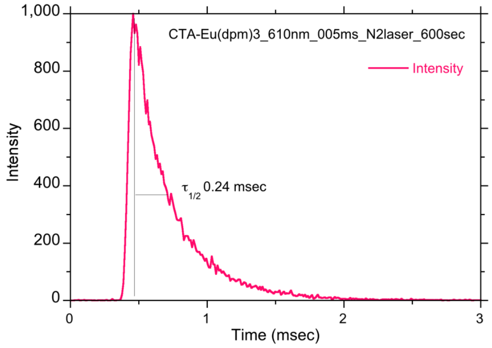

(f) $Eu(dpm)_3$ in **CTA@610nm**

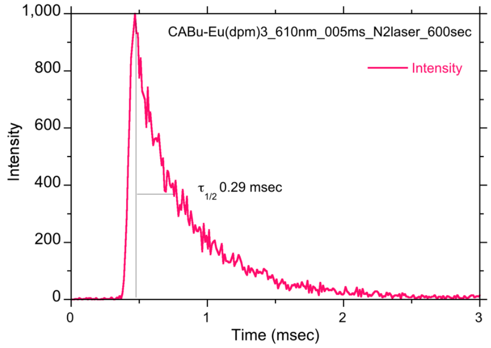

(g) $Eu(dpm)_3$ in **CABu@610nm**

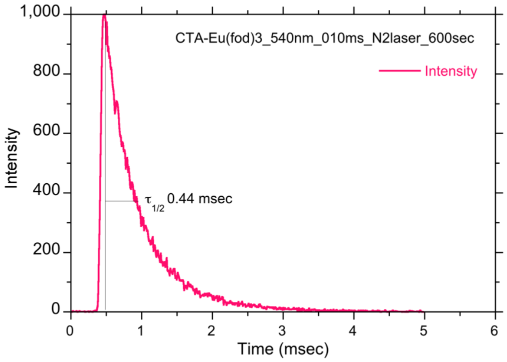

(h) $Eu(fod)_3$ in **CTA@610nm**

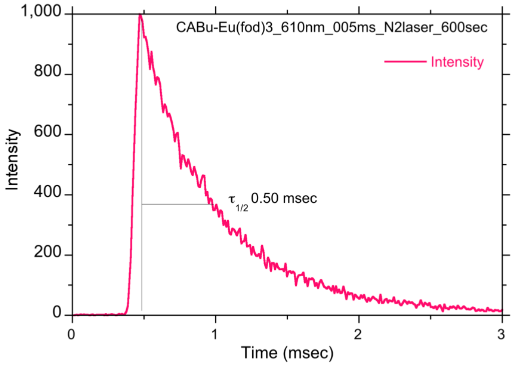

(i) $Eu(fod)_3$ in **CABu@610nm**

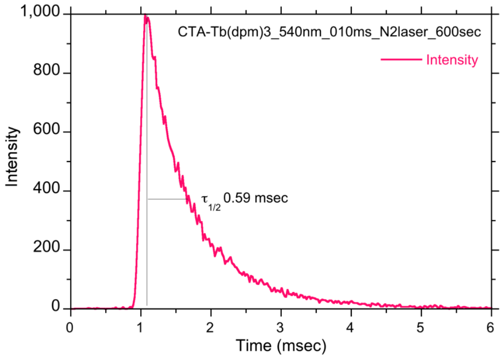

(j) $Tb(dpm)_3$ in **CTA@540nm**

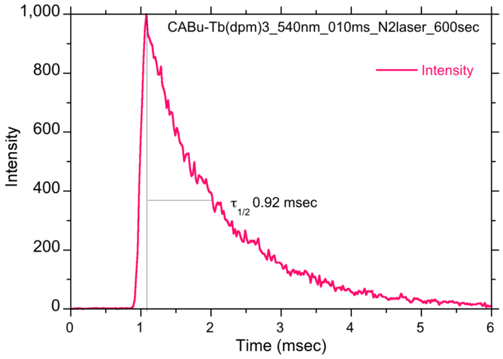

(k) $Tb(dpm)_3$ in **CABu@540nm**

Continued. Photodynamic decay curves in linear plots of (f) $Eu(dpm)_3$ in **CTA** at 610nm, (g) $Eu(dpm)_3$ in **CABu** at 610nm, (h) $Eu(fod)_3$ in **CTA** at 610nm, (i) $Eu(fod)_3$ in **CABu** at610nm, (j) $Tb(dpm)_3$ in **CTA** at 540nm, and (k) $Tb(dpm)_3$ in **CABu** at 540nm.

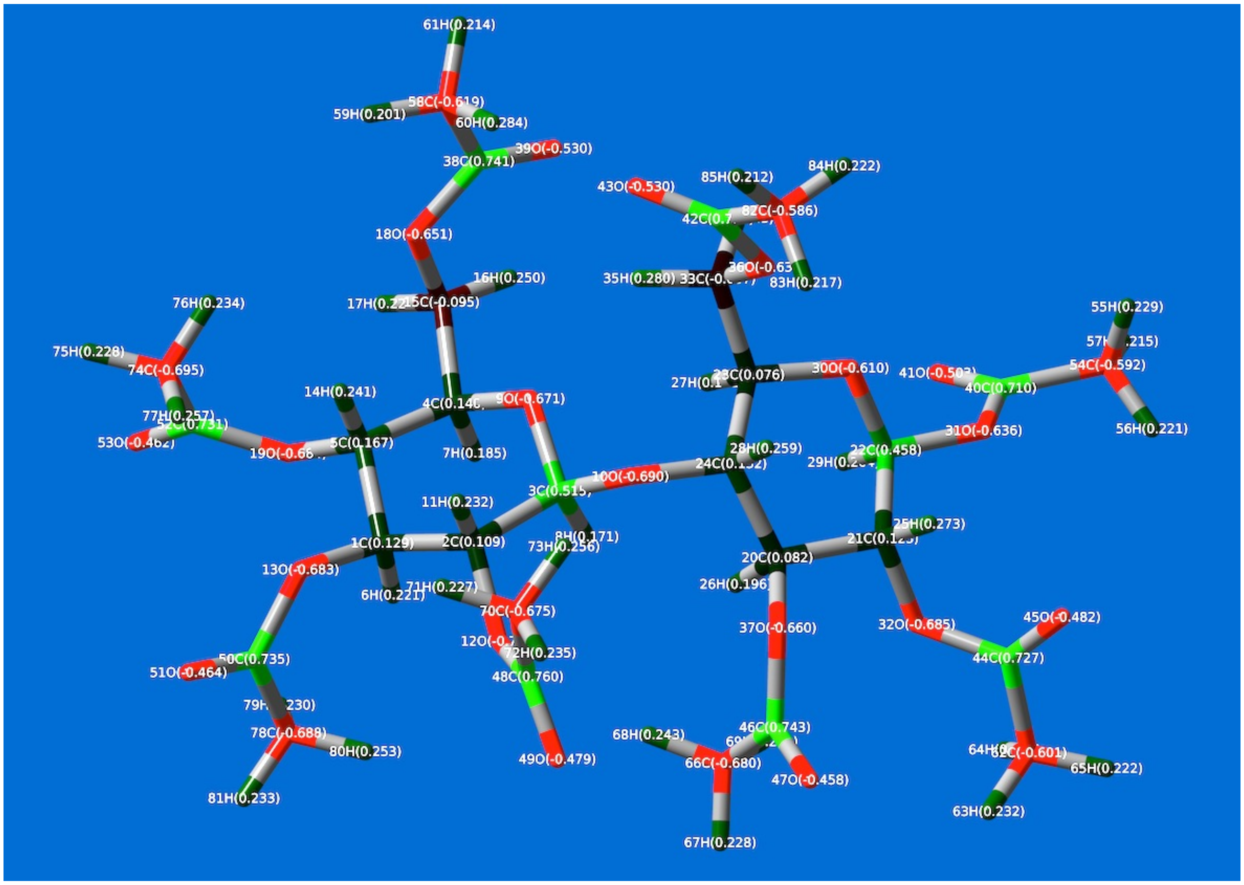

Figure S18. The Mulliken charges of ***D*-Glu dimer** as a model of **CTA** (MP2, 6-311G basis set).

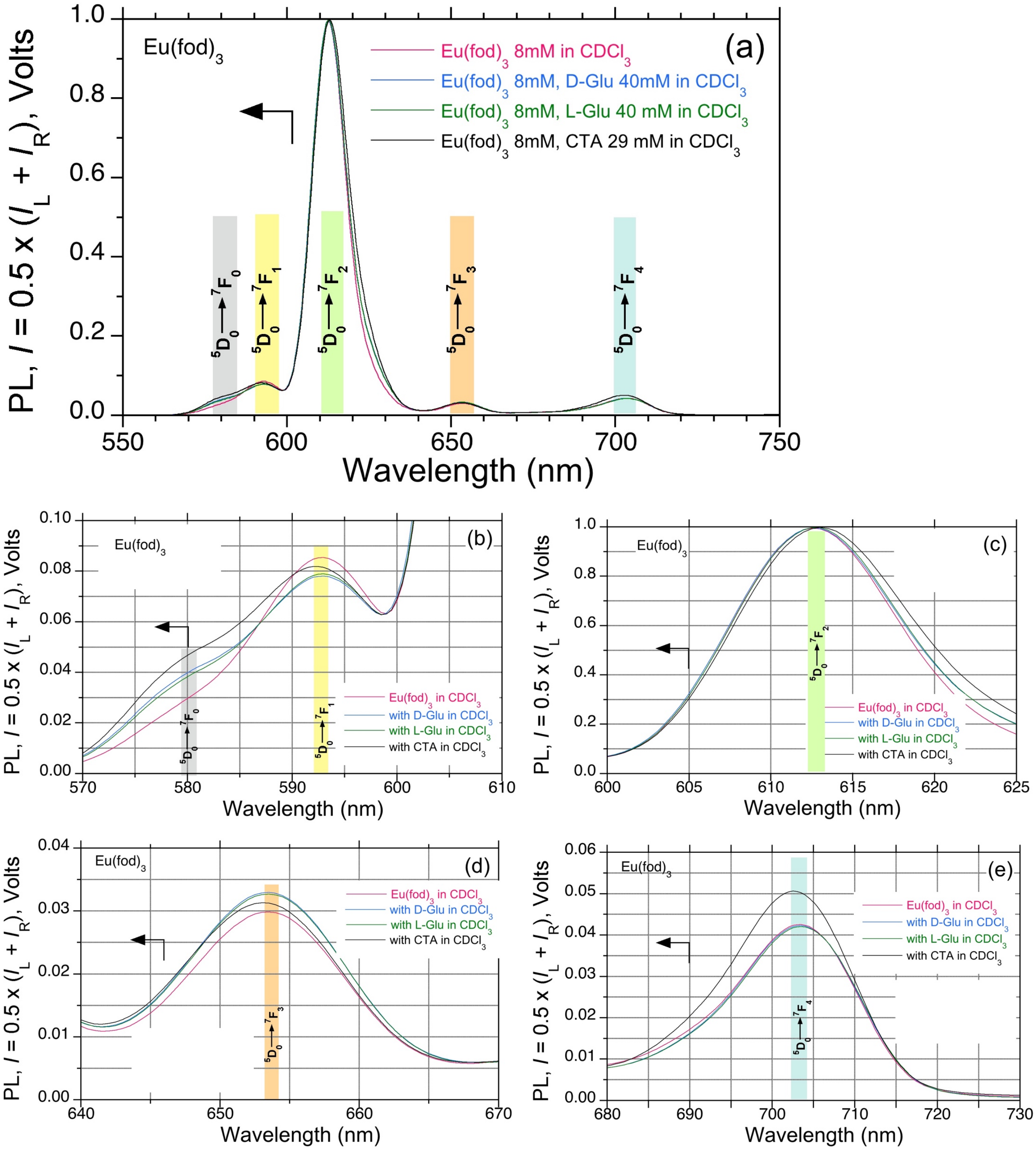

Figure S19. Normalized solution PL spectra of Eu(fod)$_3$ (10 mg, 0.8 x 10^{-2} M, red line) in the presence of ***D*-Glu** (20 mg, 4 x 10^{-2} M, blue line), ***L*-Glu** (20 mg, 4 x 10^{-2} M, green line), and **CTA** (10 mg, 3 x 10^{-2} M, black line) in 1.2 mL $CDCl_3$. Spectral regions are (a) 550 nm and 750 nm, (b) 570 nm and 610 nm, (c) 600 nm and 625 nm, (d) 640 nm and 670 nm, and (e) 680 nm and 750 nm. Excitation: 315 nm; bandwidths for excitation and emission: 10 nm and 10 nm; data interval: 0.5 nm; pathlength: 1.0 mm (see, instrumentation and Figure 4 caption in the main text).

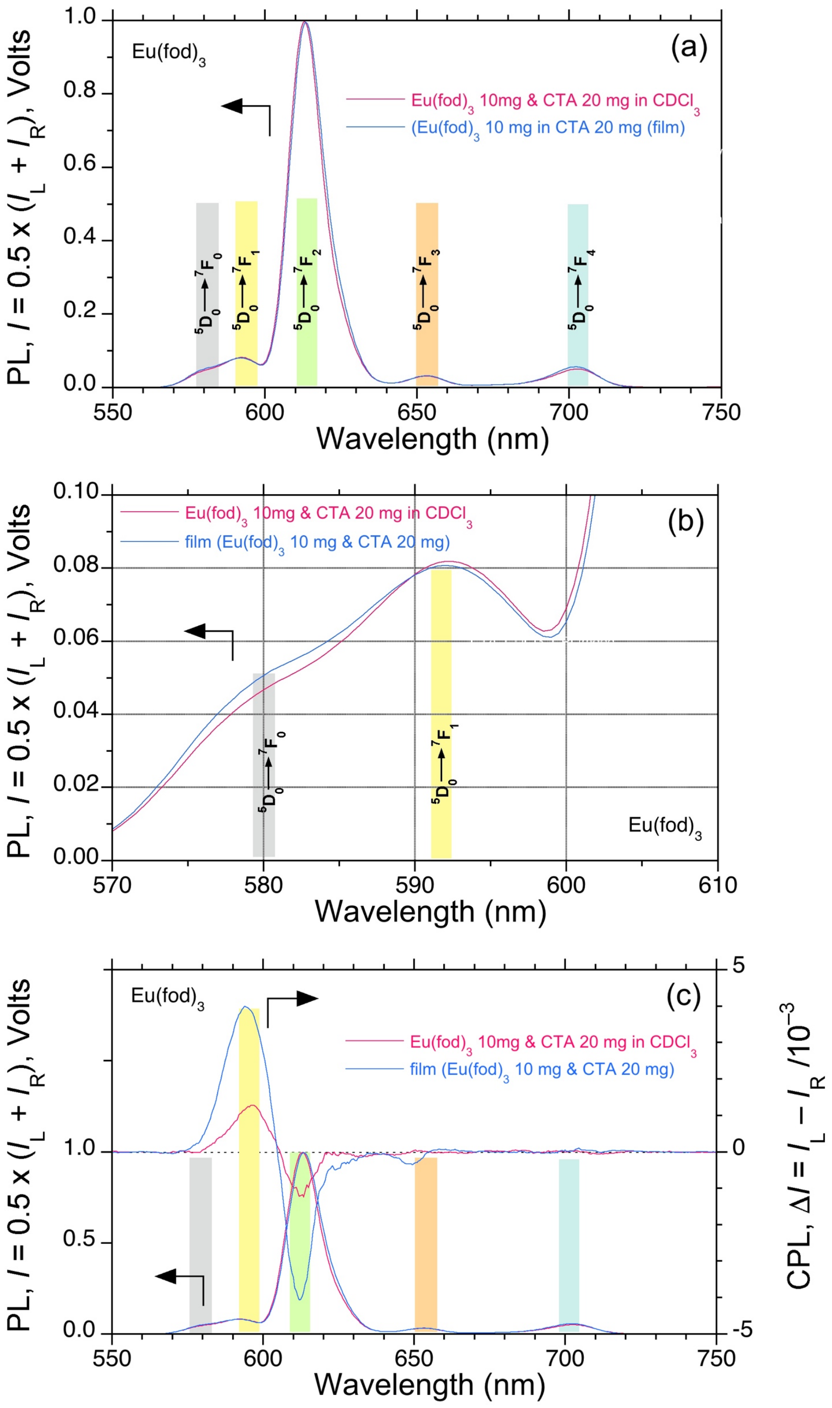

Figure S20. Comparison of PL and CPL spectra between a $CDCl_3$ solution (red line) containing Eu(fod)$_3$ (10 mg, 0.8 x 10^{-2} M) and **CTA** (20 mg, 4 x 10^{-2} M) and annealed double-side films onto Tempax substrate (~5 x 10^{-1} M in bulk CTA, 100 °C in a vacuum, overnight) by spin-coating a mixed $CHCl_3$ solution (2.0 mL) containing Eu(fod)$_3$ (10 mg) and **CTA** (20 mg). Spectral regions are (a) 550 nm and 750 nm (PL), (b) 570 nm and 610 nm (PL), (c) 550 nm and 750 nm (PL and CPL). Excitation: 315 nm (solution) and 335 nm (film); bandwidths for excitation and emission: 10 nm and 10 nm; data interval: 0.5 nm.

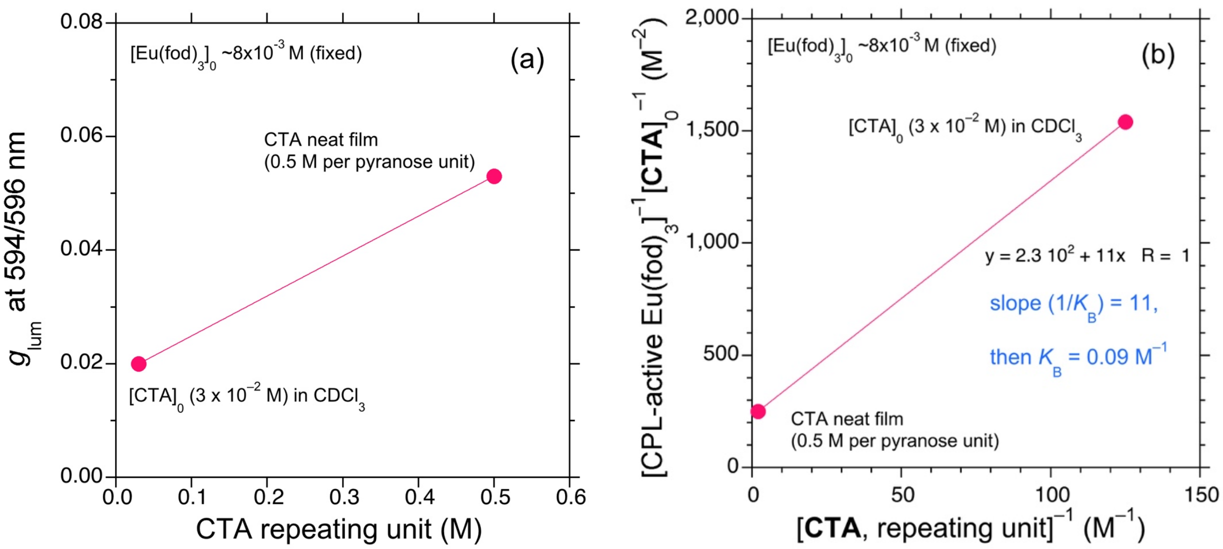

Figure S21. (a) The g_{em} value of Eu(fod)$_3$ at 594/596 nm *versus* [**CTA**, repeating unit] in M. (b) The fitting plots of [CPL-active Eu(fod)$_3$-CTA]$^{-1}$ in M^{-2} *versus* [**CTA**, repeating unit]$^{-1}$ in M^{-1}.

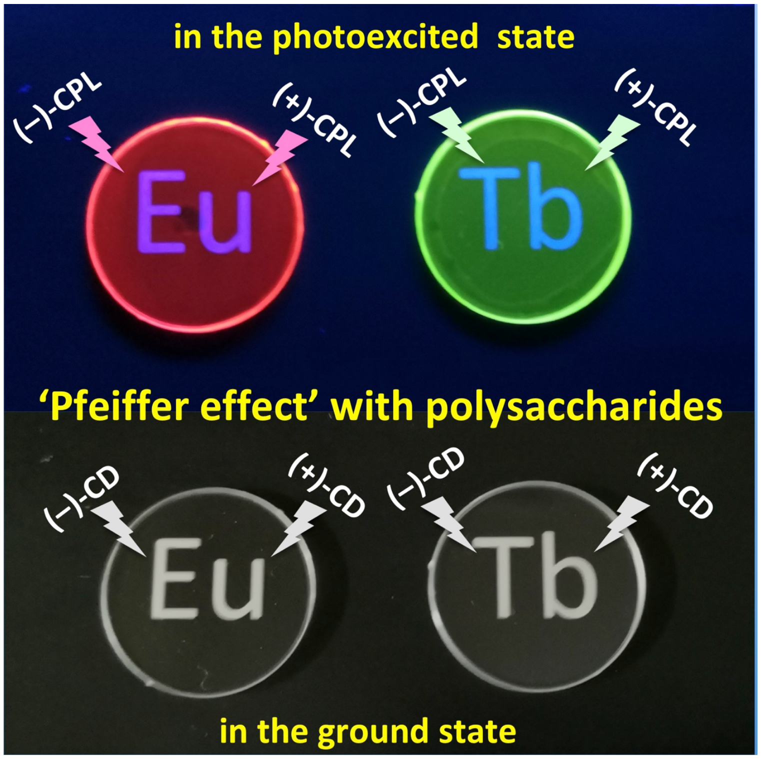

Figure S22. Four photographs of Eu(fod)$_3$ and Tb(dpm)$_3$ embedded into **CTA** films onto Tempax glass substrate placed on a black Kent paper (Daiso, Hiroshima, Japan) excited at 365 nm (high-pressure Hg light source) (top) and under room light (GaN-based LED with a sharp peak at ~450 nm and a broad peak at ~580 nm) in the absence of UV-light source (bottom).
